# Dietary *Angelica sinensis* Enhances Sow Lactation and Piglet Development Through Gut Microbiota and Metabolism

**DOI:** 10.3390/vetsci12040370

**Published:** 2025-04-15

**Authors:** Qian Chen, Yali Song, Qitian Wu, Yali Wu, Maocuo Zhou, Yifei Ren, Xiaohong Guo, Guoqing Cao, Bugao Li, Zhibian Duan, Pengfei Gao

**Affiliations:** 1 College of Animal Science, Shanxi Agricultural University, Jinzhong 030801, China; m18335220160@163.com (Q.C.); syl0542022@163.com (Y.S.); www13304056671@163.com (Q.W.); xhguo@sxau.edu.cn (X.G.); anniecao710502@aliyun.com (G.C.); bugaoli@sxau.edu.cn (B.L.); 2 Shanxi Key Laboratory of Animal Genetics Resource Utilization and Breeding, Jinzhong 030801, China; ylwu2024@163.com (Y.W.); 16609740129@163.com (M.Z.); 3 Tiankang Livestock Technology Company Limited, Zhumadian 463343, China; renyifei131@163.com; 4 College of Veterinary Medicine, Shanxi Agricultural University, Jinzhong 030801, China; dzb@sxau.edu.cn

**Keywords:** *Angelica sinensis*, lactation performance, metabolites, piglet development, gut health

## Abstract

While contemporary genetic selection strategies have successfully increased sow litter sizes, this achievement has elevated lactation demands without proportional improvements in mammary secretory capacities, ultimately constraining neonatal piglet growth and developmental outcomes. In this study, we aimed to promote piglet growth, development, and intestinal health by regulating the lactation performances of sows via the addition of *Angelica sinensis* extra (ASE) to their diets. Thirty-six third-parity Danish Landrace sows were randomly allocated to three dietary groups: 0% ASE (CON), 0.5% ASE, and 1% ASE groups, and various parameters were assessed. The addition of 0.5% ASE significantly increased sow milk production, milk-related hormone levels (*p* < 0.05), and IgA and IgG contents in the milk (*p* < 0.05) compared with 0% ASE (CON). Metabolomic analysis of sow blood revealed nine significantly upregulated metabolites specifically from AS, including coniferaldehyde, andrograpanin, and gemichalcone_C. In addition, AS influenced the abundance of beneficial gut microbiota in sow feces, such as *Lactobacillus* and the *Lachnospiraceae_AC2044_group*; promoted intestinal balance; enhanced piglet growth, immune function, and intestinal development; upregulated the expression of *Occludin*, *ZO-1*, and *Claudin-1* mRNA (*p* < 0.05); reduced serum DAO and D-LA levels (*p* < 0.05); and improved intestinal barrier function. These findings suggest that AS improves sow milk quality and piglet health by modulating serum metabolism and intestinal microbiota.

## Simple Summary

The breeding of sows with high lactation capacities is critical for improving piglet survival and growth; however, modern high-yielding sows often face challenges in milk production, leading to low-weight piglets and reduced farm profitability. In this study, we investigated the effects of adding *Angelica sinensis* (AS), a traditional Chinese herb, to the diets of lactating sows. The sows fed a diet supplemented with 0.5% AS produced significantly more milk and had higher milk-related hormone and immune protein contents. This improvement in milk quality supports improved growth, immunity, and intestinal health in piglets. Additionally, the active components of AS positively influence the gut bacteria of sows, which further enhances milk production and piglet development. Overall, in this study, we demonstrated that AS can be a valuable natural supplement for improving lactation performance in sows, ultimately benefiting piglet development and gut health.

## 1. Introduction

Piglets weaned per sow per year (PSY) is a crucial metric for assessing the reproductive performances of sows and directly affects both sow productivity and the economic profitability of pig farms [1]. The number of offspring and the survival rate of piglets during the lactation period are highly correlated with PSY [2]. Contemporary genetic selection strategies have significantly increased litter sizes; however, this advancement coincides with an increased incidence of low-birthweight piglets [3]. This phenomenon triggers a sharp surge in milk demand among piglets, and insufficient milk production potentially induces growth retardation and higher mortality rates, thereby compromising the economic efficiency of swine production chains [4]. During the lactation period, the energy and protein required by piglets are directly transmitted through sow milk, and the lactation performance is closely related to piglet growth, development, and immunity, directly impacting the preweaning growth rate and survival [5]. The weaning weight is significantly correlated with carcass traits, feed conversion efficiency, and the growth rate during the fattening period [6,7]. Therefore, in the context of modern high-producing sows, effectively enhancing their lactation capacities to improve their reproductive performances and the growth performances of piglets is a pressing issue that needs to be addressed.

*Angelica sinensis* (AS), one of the most commonly used traditional Chinese herbs in clinical practice [8], is a key component of various galactagogue formulations [9,10] and exerts multiple effects, including hormone regulation [11], the promotion of angiogenesis [12], and the inhibition of cell apoptosis [13]. The polysaccharides, terpenes, and phenolic compounds in AS demonstrate potent free radical-scavenging properties [14]. Wang [15] reported that feeding AS polysaccharides to mice with liver damage suppressed the release of the proinflammatory cytokines TNF-α, IFN-γ, IL-2, and IL-6; reduced the production of reactive oxygen species (ROS) and malondialdehyde (MDA); and exhibited strong antioxidant effects. AS polysaccharides alleviate mastitis and blood–milk barrier damage in mice by modulating their intestinal microbiota [16]. Supplementation with Bazhen powder containing AS significantly increased the fat and protein contents in milk [17], enhancing the antioxidant capacities of piglets [18]. However, research on the isolated use of AS during lactation is lacking.

In this study, we address the declining piglet survival rates resulting from the increased litter sizes but insufficient lactation performances of modern high-yielding sows. The research subject was AS, a traditional medicinal and edible Chinese herb, which we used to innovatively explore the application of monomeric formulations, breaking away from traditional compound compatibility patterns. The regulatory mechanisms of sow lactation performance were systematically evaluated by supplementing the diets of lactating sows with graded levels of AS powder (0%, 0.5%, and 1%). We analyzed the effects of maternal–neonatal integration on the growth performance and intestinal barrier function of suckling piglets. The findings provide empirical evidence to address the challenges of the high cost and ambiguous mechanisms of action associated with herbal feed additives, and they expand the theoretical dimensions of maternal–neonatal synergistic nutritional regulation in livestock.

## 2. Materials and Methods

### 2.1. Animal Husbandry and Experimental Design

**Figure 1:**
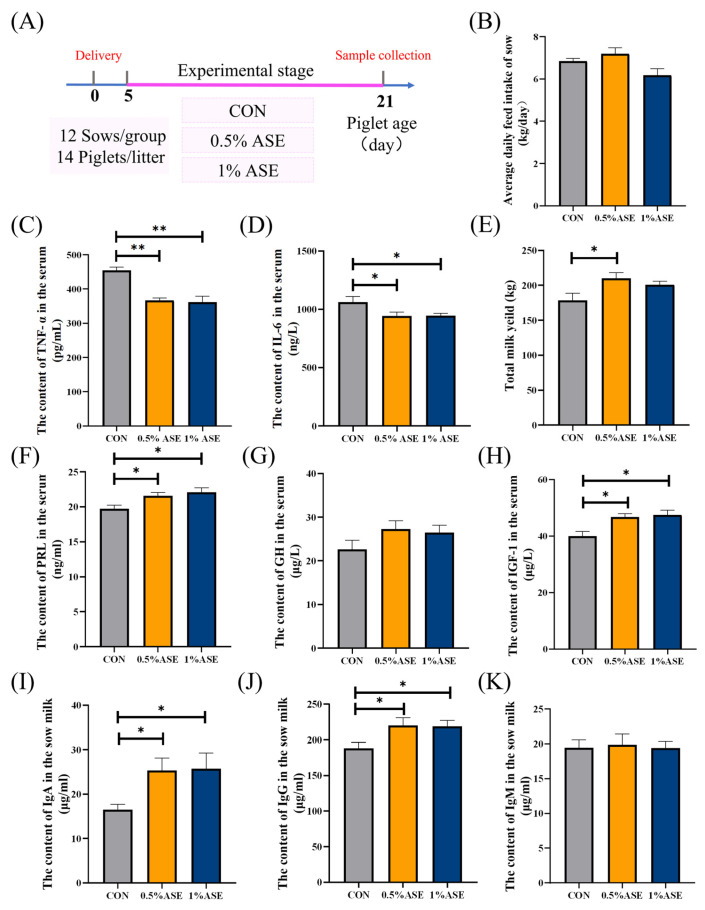
Effects of AS on sow milk yields and immunoglobulin contents: (**A**) experimental design; (**B**) analysis of feed intake in sows; analysis of (**C**) TNF-α, (**D**) IL-6, and (**E**) total milk yield in sows; analysis of (**F**) PRL, (**G**) GH, and (**H**) IGF-1 levels in sow sera; analysis of (**I**) IgA, (**J**) IgG, and (**K**) IgM levels in sow milk. TNF-α: Tumor necrosis factor-α; IL-6: Interleukin-6; PRL: Prolactin; GH: Growth Hormone; IGF-1: Insulin-like growth factor 1; IgA: Immunoglobulin A; IgG: Immunoglobulin G; IgM: Immunoglobulin M. * *p* < 0.05; ** *p* < 0.01.

Thirty-six third-parity Danish-bred Landrace sows were housed at Sanmenzha Farm of Ru’nan Tiankang Hongzhan Feed Technology Co., Ltd. (Zhumadian, China). The sows were randomly divided into three groups of 12 sows each: a control group fed a basal diet (CON), a group fed the basal diet supplemented with 0.5% AS extra (0.5% ASE), and a group fed a basal diet supplemented with 1% AS extra (1% ASE). The formulation and nutritional profile of the sow feed rations are shown in Table 1. Fresh AS was purchased from Gansu Province, washed, dried, ground using a crusher, sieved through a 60-mesh screen, and stored. Feeding commenced on the fifth day postpartum and continued until the piglets were weaned on the twenty-first day, as outlined in Figure 1A. Each sow was adjusted to nurse 14 piglets per litter, with an initial litter weight of 25.45 kg ± 1.09 kg. The sows were fed four times daily at 7:00, 11:00, 15:00, and 19:00, with 2 kg of feed per feeding. The feed intake was measured by weighing and recording the leftover feed after each feeding. The sows and piglets had ad libitum access to water throughout the trial.

### 2.2. Sample Collection

The feeding trial was conducted until the piglets were weaned at 21 days of age, and the biological samples were collected from both the sows and piglets during the terminal phase. Five sows were randomly selected from each group, and 2 mL of blood was collected from their ear veins. Additionally, 2 mL of milk from the front, middle, and rear parts of the sows’ udders was collected, mixed, and stored in liquid nitrogen. Fecal samples were collected from the rectums of five sows per experimental group and were immediately preserved in 2 mL cryogenic vials under liquid nitrogen storage. One piglet was selected from each group of five piglets born to the selected sows. Blood was collected from the anterior vena cava of each piglet, left to stand for 30 min, and centrifuged, after which the supernatant was stored at −80 °C. After the piglets were euthanized, tissue samples from the duodenum, jejunum, and middle segment of the ileum were fixed in 4% paraformaldehyde, and samples of tissue and contents from various segments of the small intestine were also collected and stored in liquid nitrogen. All samples were frozen in liquid nitrogen, except for the serum samples.

### 2.3. Calculation of Sow Lactation Capacity

During the experimental period, the weight gain of the piglets was assessed to evaluate the lactation capacities of the sows via the following formula [19]:

Total milk yield = piglet ADG × litter size × lactation days × 4

### 2.4. Average Daily Weight Gain of Piglets

The initial and weaning weights of the piglets were recorded at the start of the experiment to calculate the average daily weight gain.

### 2.5. Enzyme-Linked Immunosorbent Assay

ELISA kits were purchased from Jiangsu Meimian Industrial Co., Ltd. (Yancheng, China). Following the instructions, the IgG (MM-040301), IgA (MM-090501), and IgM (MM-040201) concentrations in the sow milk were measured, as well as the TNF-α (MM-038302), IL-6 (MM-041802), PRL (MM-090702), IGF-1 (MM-7807502), and GH (MM-039701) concentrations in the sow sera. Additionally, the IgG, IgA, IgM, T3 (MM-191802), T4 (MM-191902), GH, DAO (MM-043802), and D-LA (MM-3373201) levels in the piglet sera and the SIgA (MM-3623402) contents in the piglet intestinal tissues were assessed.

### 2.6. Intestinal Histomorphology Analysis

Following fixation in 4% paraformaldehyde for 24 h, the intestinal tissues were dehydrated, cleared, and embedded to produce tissue blocks. The subsequent steps included sectioning, deparaffinization, H&E staining, slide preparation for photography, and the use of ImageJ software (V 1.53k, Bethesda, MD, USA) for quantifying the villus length and crypt depth to determine the villus–crypt ratio.

### 2.7. Real-Time PCR for Gene Expression Analysis

Total RNA was extracted from the intestinal tissues following the protocol of the SevenFast^®^ Total RNA Extraction Kit (Seven, Beijing, China). The RNA concentration was measured via a spectrophotometer (Nanodrop, Waltham, MA, USA), and cDNA synthesis was then performed via the All-in-One RT EasyMix for the qPCR Kit (Tolobio, Shanghai, China). qRT-PCR was conducted on a fluorescence quantitative PCR instrument (Bio-Rad, Hercules, CA, USA) with 2 × Q3 SYBR qPCR Master Mix (Tolobio, Shanghai, China) under the following conditions: 95 °C for 30 s; 40 cycles of 95 °C for 10 s and 60 °C for 30 s; and a final step of 95 °C for 10 s and a melt curve from 65 °C to 95 °C with an increment of 0.5 °C for 5 s. The primers used in this experiment were supplied by Sangon Biotech Co., Ltd. (Shanghai, China), and their sequences are listed in Table 2.

### 2.8. Analysis of Serum and Angelica sinensis Metabolites

AS powder (50 mg ± 2 mg) was mixed with beads and 500 μL of an extraction solution composed of MeOH:ACN:H_2_O at a ratio of 2:2:1 (*v*/*v*/*v*) containing deuterated internal standards. The mixture was vortexed for 30 s, homogenized at 35 Hz for 240 s, and sonicated for 5 min in a 4 °C water bath. The homogenization and sonication processes were repeated three times. Subsequently, the mixture was incubated for 30 min at −40 °C to precipitate proteins, and the samples were centrifuged at 12,000 rpm (RCF = 13,800× *g*, R = 8.6 cm) for 15 min at 4 °C. The resulting supernatant was transferred to fresh vials, incubated for 10 min, and centrifuged again at 12,000 rpm (RCF = 13,800× *g*, R = 8.6 cm) for 15 min at 4 °C, and the new supernatant was then transferred to fresh glass vials for analysis. A quality control (QC) sample was prepared by combining equal aliquots of the supernatant from the samples.

The sera (200 μL) were mixed with 20 μL of a 2 mol/L hydrochloric acid solution. After vortexing for 30 s and sonication for 5 min in a 4 °C water bath, 780 μL of acetonitrile was added to the samples. The new mixtures were vortexed for 30 s, sonicated for 5 min in a 4 °C water bath, and then incubated at −40 °C for 30 min to precipitate proteins. Subsequently, the samples were centrifuged at 12,000 rpm (RCF = 13,800× *g*, R = 8.6 cm) for 15 min at 4 °C. The supernatant liquids (800 μL) were evaporated, and 80 μL of an extraction solution (MeOH:ACN:H_2_O, 2:2:1 *v*/*v*/*v*) containing deuterated internal standards was added to dissolve the metabolites. The final solutions were vortexed for 30 s, sonicated for 1 min in a 4 °C water bath, and then centrifuged at 12,000 rpm for 15 min at 4 °C. The resulting supernatant liquids were transferred to fresh glass vials for analysis. A quality control (QC) sample was prepared by combining equal aliquots of the supernatants from the samples.

LC-MS/MS analyses were conducted using a Vanquish UHPLC system (Thermo Fisher Scientific, Waltham, MA, USA) coupled to an Orbitrap Exploris 120 mass spectrometer (Thermo) with a Phenomenex Kinetex C18 column (2.1 mm × 100 mm, 2.6 μm). The mobile phases consisted of 0.01% acetic acid in water (A) and a mixture of IPA and ACN (1:1, *v*/*v*) (B). The auto-sampler temperature was maintained at 4 °C with an injection volume of 2 μL. The Orbitrap Exploris 120 mass spectrometer was chosen for its ability to perform MS/MS spectrum acquisition in information-dependent acquisition (IDA) mode using Xcalibur software (V 4.4). The ESI source conditions were set as follows: sheath gas flow rate: 50 Arb; aux gas flow rate: 15 Arb; capillary temperature: 320 °C; full MS resolution: 60,000; MS/MS resolution: 15,000; collision energies: 20/30/40; and spray voltages: 3.8 kV (positive) or −3.4 kV (negative).

The original data were compared with Biotree TCM (V 1.0) and BT-HERB (V 1.0) for metabolite identification, followed by visualization using the R package [20].

### 2.9. Analysis of 16S rRNA Gene Amplicons from Gut Microbiota

Fecal sample DNA was extracted via a fecal DNA extraction kit (TIANGEN, Beijing, China). The V3-V4 region was PCR-amplified with the primers 341F (CCTAYGGGRBGCASCAG) and 805R (GGACTACNNGGGTATCTAAT), followed by purification and quantification via the AxyPrep DNA Gel Extraction Kit (Axygen, Hangzhou, China). Sequencing was performed on the Illumina platform. The raw sequences were processed in QIIME 2 via the DADA2 plugin for quality control, denoising, merging, and chimera removal to generate amplicon sequence variants (ASVs). Differential analysis of the microbial communities was performed via the Kruskal-Wallis test, the diversity was assessed via the core-diversity plugin and visualized in R, and the functional predictions were made via PICRUSt2 software (V 2.5.2).

### 2.10. Data Statistics and Analysis

The data analysis was conducted via SPSS 26.0 (IBMCorp, Armonk, NY, USA). The Student’s *t*-test was used to compare the differences between the two groups, and one-way ANOVA was used to analyze the differences among multiple groups. Duncan’s post hoc test was applied for multiple comparisons. The results with *p* < 0.01 were considered highly significant, those with *p* < 0.05 were deemed statistically significant, and differences with *p* < 0.1 indicated a trend.

## 3. Results

### 3.1. Dietary AS Supplementation Enhances Lactation Performance and Milk Immunoglobulin Profiles in Sows

The analysis revealed that AS had no significant effect on the feed intake of the lactating sows (Figure 1B). The serum inflammatory cytokine analysis revealed that the TNF-α levels were significantly reduced in both the 0.5% ASE and 1% ASE groups (*p* < 0.01, Figure 1C) compared with those in the CON group, while the IL-6 levels were also significantly decreased in these two groups (*p* < 0.05, Figure 1D). Calculation of the sow milk production based on the daily weight gain of the piglets showed that compared with the control group, the 0.5% ASE group had significantly increased sow milk production (*p* < 0.05), with an average increase of 25.10 kg. Although the milk production in the 1% ASE group increased by approximately 16.01 kg, it did not reach a significant level (Figure 1E). The findings indicated a significant elevation in the PRL and IGF-1 levels in the sera of the sows in the 0.5% ASE and 1% ASE groups (*p* < 0.05; Figure 1F,G), with the GH displaying an upward trend (0.1 < *p* < 0.05; Figure 1H). An examination of the immunoglobulin content in the sow milk demonstrated that both 0.5% ASE and 1% ASE led to significant increases in the IgA and IgG levels (*p* < 0.05; Figure 1I,J), with no observed effect on the IgM concentrations in the sow milk (Figure 1K).

### 3.2. AS Metabolite Analysis

**Figure 2:**
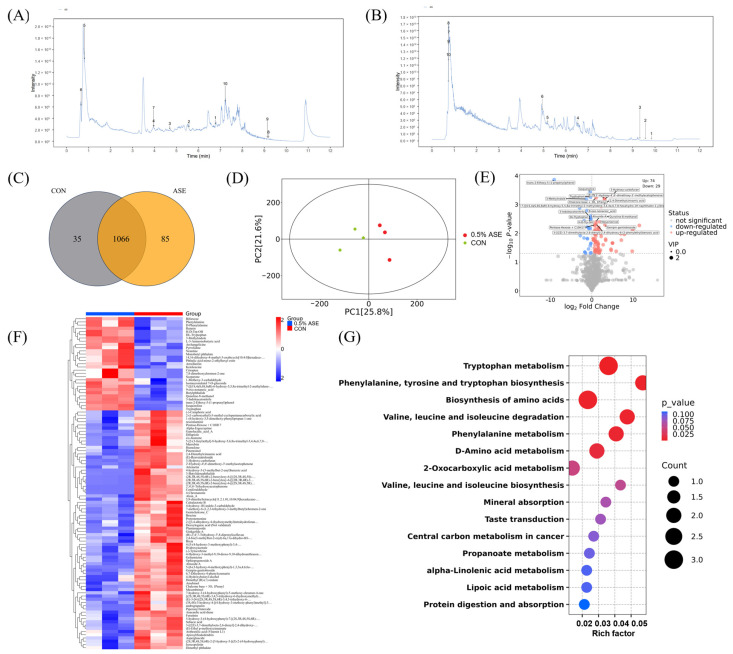
Metabolic analysis of AS and sera from sows. Top 10 metabolite TICs from AS samples in (**A**) positive and (**B**) negative ion modes. Analysis of AS metabolites, retention time (x-axis) and ion current intensity (y-axis) (**C**) Results of metabolite Venn diagram analysis; (**D**) score scatter plot of PCA model; (**E**) volcano plot of differential metabolites, where the x-axis represents fold changes, the y-axis represents *p*-values, and the sizes of the dots reflect VIP values. Red indicates significant upregulation, blue indicates significant downregulation, and gray represents non-significant differences; the x-axis represents experimental groups, the y-axis displays differential metabolites, and the color blocks indicate the relative expression levels of the metabolites, with red indicating high expression and blue indicating low expression. (**F**) Heatmaps of upregulated and downregulated metabolites. (**G**) Enrichment results of metabolite KEGG pathways, where the Rich Factor for each pathway is displayed on the x-axis and the KEGG metabolic pathway names are displayed on the y-axis. The sizes of the dots correspond to the numbers of differentially enriched metabolites within each pathway. The color gradient indicates the significance of the *p*-value, with a shift towards red hues signifying a higher enrichment significance level.

A total of 1417 metabolites were detected in both the positive and negative ion modes, with 698 and 719 metabolites identified in the positive and negative ion modes, respectively. Various substances were identified, including fatty acids and conjugates (8.26%), flavonoids (7.20%), small peptides (6.77%), and phenylpropanoids (5.86%). The top 10 substances in the total ion chromatograms (TICs) for the positive and negative ion modes are shown in Figure 2A and Figure 2B, respectively. Detailed information on the specific AS metabolic components is provided in Supplementary Table S1.

### 3.3. Impact of AS Supplementation on Serum Metabolism in Sows

We selected the CON and 0.5% ASE groups for the subsequent analyses of the lactation volumes in the sows. The control group yielded 1101 detected compounds (553 positive ion products and 548 negative ion products) and the 0.5% ASE group yielded 1151 detected compounds (585 positive ion products and 566 negative ion products), with 1066 compounds common to both groups (Figure 2C). The principal component analysis, based on the relative metabolite levels, clearly distinguished the control group from the 0.5% ASE group (Figure 2D). An analysis of the metabolite level changes revealed 74 significantly upregulated metabolites (31 positive ion products and 43 negative ion products) and 29 significantly downregulated metabolites (20 positive ion products and nine negative ion products) (Figure 2E). Compared to the control group, the 0.5% ASE group exhibited the upregulation of 13 substances by over 20-fold, including 2′,4′,6′-Trihydroxyacetophenone, Coniferaldehyde, and 4-hydroxy-3-(3-methylbut-2-enyl)benzoic acid, while 6 substances were upregulated by 5–20-fold, including Ganolucidic_acid_A, Gemichalcone_C, and Genipin-gentiobioside (Figure 2F and Supplementary Table S2). In addition, following the consumption of AS during lactation, the sows exhibited notable decreases of more than 5-fold in trans-2-Ethoxy-5-(1-propenyl)phenol, 7-[[(1S,4aS,6S,8aR)-6-hydroxy-5,5,8a-trimethyl-2-methylidene-3,4,4a,6,7,8-hexahydro-1H-naphthalen-1-yl]methoxy]chromen-2-one, and archangelicine, while 26 other compounds, such as armillarilin, showed significant reductions of 1–5-fold (Figure 2G and Supplementary Table S3). The KEGG pathway enrichment analysis of differential metabolites revealed significant enrichment in pathways such as tryptophan metabolism; phenylalanine, tyrosine, and tryptophan biosynthesis; the biosynthesis of amino acids; valine, leucine, and isoleucine degradation; and phenylalanine and D-amino acid metabolism (Figure 2G, *p* < 0.05).

### 3.4. Analysis of Important AS Metabolites in Blood

The 0.5% ASE group exhibited a significant upregulation of metabolic products compared with the control group, potentially influencing the lactation performances of the sows. A comparison between the significantly upregulated metabolites in the 0.5% ASE group and those in the AS group revealed the presence of nine metabolites that were not detected in the CON group, including six cationic and three anionic metabolites (Table 3). Among these, five metabolites in the 0.5% ASE group, including 2′,4′,6′-trihydroxyacetophenone, coniferaldehyde, and plantamajoside, showed concentrations exceeding 30 times those in the CON group.

### 3.5. AS Effects on Intestinal Microbiota in Sows

**Figure 3:**
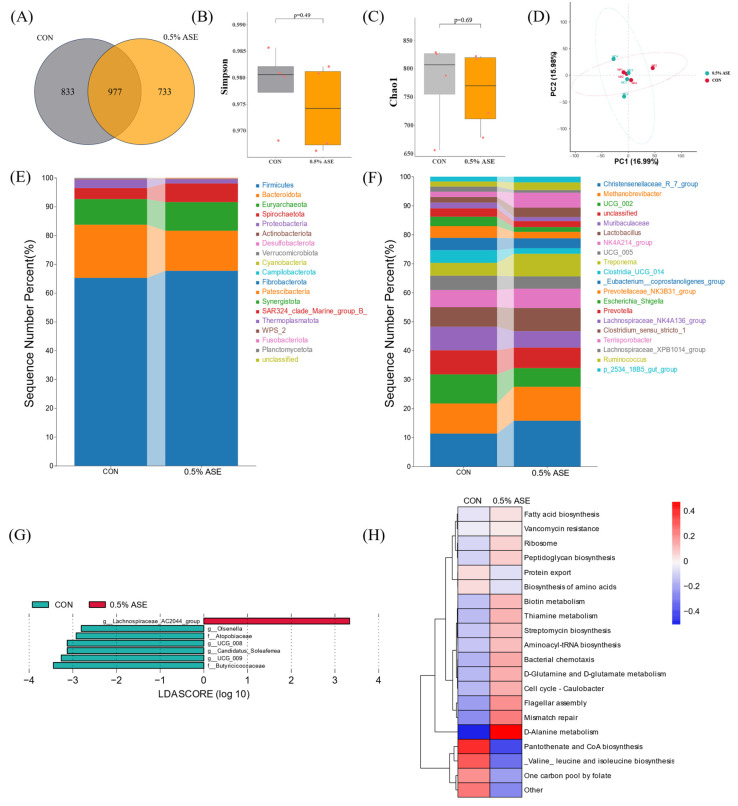
Microbial community analysis of sow feces: (**A**) analysis of OTU compositions in CON and 0.5% ASE groups; evaluation of alpha diversity of gut microbiota in sows using (**B**) Simpson and (**C**) Chao1 indices; (**D**) principal component analysis of gut microbiota; compositions of microbial abundances at the (**E**) phylum and (**F**) genus levels; (**G**) LEfSe analysis of gut microbiota, where the green LDA scores represent bacteria enriched in the CON group and the red LDA scores signify bacteria enriched in the 0.5% ASE group; (**H**) prediction of microbial functions, where red indicates a positive correlation and blue indicates a negative correlation.

To investigate whether the gut microbiota is involved in regulating the lactation performances of sows, 16S rRNA sequencing analysis was conducted on sow fecal samples. We analyzed the microbial diversity in the rectums of lactating sows, obtaining a total of 2583 OTUs, with 1810 in the CON group, 1750 in the 0.5% ASE group, and 977 shared between the two groups (Figure 3A). The diversity (Figure 3B) and species richness (Figure 3C) of the intestinal microbiota in the sows were not significantly affected by the AS treatment (*p* > 0.05). Principal component analysis revealed no distinct separation in the microbial community structure (Figure 3D). At the phylum level, the annotation results revealed that Firmicutes (65.24%, 67.71%) and Bacteroidota (18.46%, 13.92%) were the dominant phyla in the CON and 0.5% ASE groups. Compared with the CON group, Bacteroidota was significantly decreased in the 0.5% ASE group (Figure 3E, *p* < 0.05), and the Firmicutes/Bacteroidota ratio was also significantly enhanced (*p* < 0.05). The Euryarchaeota (8.91%, 9.93%), Spirochaetota (3.81%, 6.49%), and Proteobacteria (2.92%, 1.43%) abundances were relatively high. At the genus level, the AS impact led to increases in the abundances of the *Christensenellaceae_R_7_group* (9.71%, 13.10%), *Methanobrevibacter* (8.90%, 9.72%), *Lactobacillus* (5.76%, 6.65%), *Treponema* (3.78%, 6.47%), and *Terrisporobacter* (1.56%, 4.26%) compared with those of the CON group. Conversely, the abundances of *UCG_002* (8.56%, 5.36%), *Muribaculaceae* (6.94%, 4.71%), *Clostridia_UCG_014* (3.75%, 1.59%), the *Prevotellaeae_NK3B31_group* (3.46%, 1.85%), and *Escherichia_Shigella* (2.76%, 1.36%) decreased (Figure 3F). LEfSe analysis revealed that *Olsenella*, Atopobiaceae, *UCG_008*, *Candidatus_Soleaferrea*, *UCG_009*, and Butyricicoccaceae, among other genera, were specifically enriched in the CON group, and that the *Lachnospiraceae_AC2044_group* had the highest LDA value in the 0.5% ASE group (Figure 3G). The microbial function predictions indicated that the 0.5% ASE group could enhance intestinal D-alanine metabolism, mismatch repair, and flagellar assembly, while reducing pantothenate and CoA biosynthesis, valine, leucine, and isoleucine biosynthesis, and one carbon pool by folate functions (Figure 3H).

### 3.6. Maternal AS Intake During Lactation Enhances Piglet Growth and Immune Function

**Figure 4:**
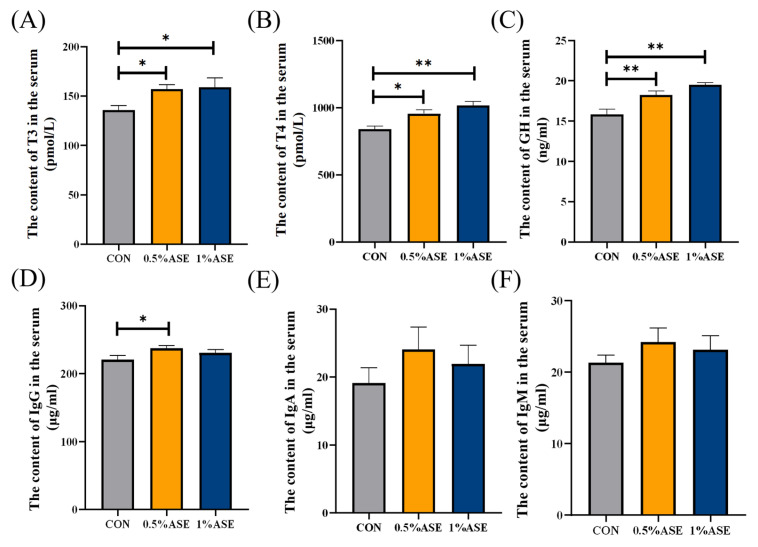
Maternal dietary AS supplementation influences piglet growth and immune response. Changes in the levels of serum (**A**) T3, (**B**) T4, (**C**) GH, (**D**) IgG, (**E**) IgA, and (**F**) IgM in piglets. T3: Triiodothyronine; T4: Thyroxine; GH: Growth Hormone; IgA: Immunoglobulin A; IgG: Immunoglobulin G; IgM: Immunoglobulin M. * *p* < 0.05; ** *p* < 0.01.

The growth performance of suckling piglets is a crucial indicator of their lactation capacity; thus, parameters, including body weights, blood hormone levels, and immune-related indicators, were measured during the suckling phase. As shown in Table 4, the piglets presented significant increases in their body weights starting from day 15, when the sows were fed 0.5% ASE (*p* < 0.05). The weaning weights (*p* < 0.05) and average daily gains (*p* < 0.05) of the piglets were significantly greater than those of the control piglets, while those of the 1% ASE group were not significantly different. Feeding the sows with AS significantly elevated the GH expression in the piglets. The 0.5% ASE treatment notably increased the T3 and T4 concentrations in the blood (*p* < 0.05; Figure 4A,B) and significantly elevated the GH levels (*p* < 0.01; Figure 4C), while the 1% ASE treatment significantly increased the serum T4 and GH levels (*p* < 0.01; Figure 4B,C) with the increasing AS concentrations. In terms of the immune indicators, 0.5% ASE significantly increased the IgG levels in the piglet sera (*p* < 0.05; Figure 4D), whereas the IgA and IgM levels increased but did not reach statistical significance (*p* > 0.05; Figure 4E,F).

### 3.7. Supplementing Sow Diets with AS Benefits the Intestinal Health of Piglets

**Figure 5:**
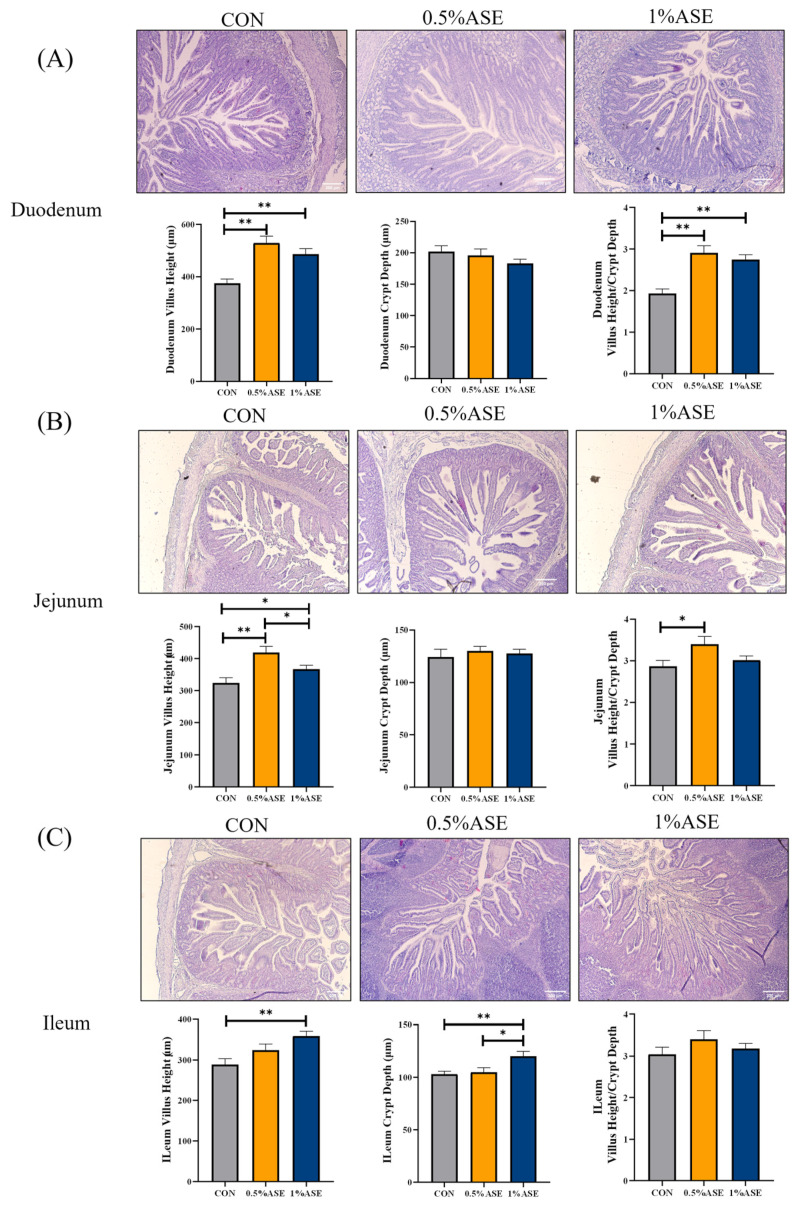
AS consumption by lactating sows enhances the intestinal development of piglets. (**A**) Duodenum, (**B**) jejunum, and (**C**) ileum tissue sections, along with villus length, crypt depth, and villus length–crypt depth statistical data. * *p* < 0.05; ** *p* < 0.01.

**Figure 6:**
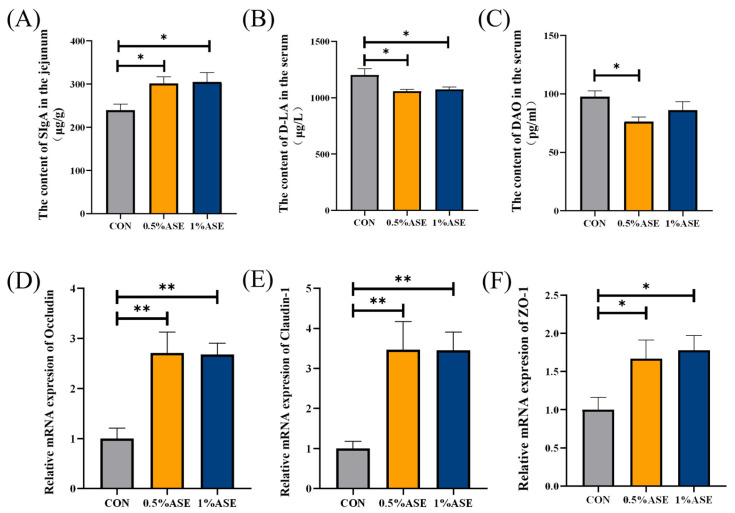
Consuming AS during lactation enhances the intestinal barriers of piglets. (**A**) Levels of SIgA in the jejuna of piglets; concentrations of (**B**) D-LA and (**C**) DAO in piglet sera; expression of (**D**) Occludin, (**E**) Claudin-1, and (**F**) ZO-1 mRNA in the jejuna of piglets. SIgA: Secretory Immunoglobulin A; D-LA: D-Lactate; DAO: Diamine Oxidase; ZO-1: Zonula Occludens Protein 1. * *p* < 0.05; ** *p* < 0.01.

The effect of the AS consumption by the lactating sows on the intestinal health of the piglets was evaluated by examining the morphological structure and barrier function of the small intestines of the latter. Morphological observations of the small intestine (Figure 5) showed that 0.5% ASE and 1% ASE significantly increased the villi heights and villus height–crypt depth ratios in the duodena (*p* < 0.01) and jejuna (*p* < 0.05) of the piglets, with no significant difference in the crypt depths (*p* > 0.05). Both 0.5% ASE and 1% ASE significantly increased the villi heights and crypt depths in the ilea of the piglets (*p* < 0.01), with no significant difference in the villus height–crypt depth ratios (*p* > 0.05). The ELISA results revealed a significant increase in the SIgA levels in the jejunal mucosae of the piglets (Figure 6A, *p* < 0.01) and a significant decrease in the serum DAO and D-LA levels (Figure 6B,C, *p* < 0.05). The qRT-PCR analysis of the intestinal tight junction protein expression in the jejuna of the piglets showed that the additional consumption of AS by the lactating sows significantly increased the expression of *Occludin* and *Claudin-1* mRNA in the intestines of their offspring (Figure 6D,E, *p* < 0.01), as well as the *ZO-1* mRNA expression (Figure 6F, *p* < 0.05). In conclusion, the results indicate that AS consumption by lactating sows can enhance the intestinal barrier function of piglets and promote intestinal maturation.

## 4. Discussion

Milk production by sows is a crucial element that impacts the growth and development of piglets [21]. Increasing milk intake can reduce piglet mortality from 12.2% to 9.8%, thereby enhancing the productivity of sows and improving the economic efficiency of farming [22]. During the late-gestation through lactation phases, maternal dietary supplementation with tapioca polysaccharide iron [23], yeast-derived nucleotides [24], and oregano essential oils [25] has been demonstrated to enhance sow lactation capacity, resulting in improved piglet growth trajectories and developmental outcomes. For postpartum recovery and offspring health, postpartum women benefit from traditional Chinese medicine formulas containing AS, such as Si-Wu-Tang and Sheng-Hua-Tang [26]. Research on the lactation effects of AS and its extracts when used alone is currently scarce. Owing to the blood circulation-promoting properties of AS in traditional Chinese medicine, in this study, we opted to commence the experimentation on the 5th day postpartum to prevent any unfavorable effects on the postpartum sows. AS significantly elevated the sow lactation capacity and associated hormone levels and prevented adverse effects of AS consumption on the maternal system, enhancing the immunoglobulin content in the lactating sow milk.

During the lactation period, sows require substantial energy and nutrients to maintain milk synthesis. Insufficient feed intake may trigger the mobilization of body fat reserves, leading to weight loss and reducing milk secretion and quality [27]. In this study, the sows fed with 0.5% ASE showed a slight increase in their feed intake, while the 1% ASE group exhibited a downward trend in their feed consumption, which may be attributed to the excessively high additive concentration, which could have resulted in a strong herbal odor in the feed, negatively affecting the sow feed intake and ultimately failing to significantly enhance the lactation performance.

Lactation is a hormonally regulated physiological process. PRL is the primary hormone responsible for inducing and sustaining lactation, promoting it through various pathways, such as by increasing the proliferation of mammary epithelial cells, stimulating protein synthesis and secretion, and regulating calcium metabolism [28,29]. Codonopsis lanceolata polysaccharides enhanced the postpartum lactation deficiency induced by a high-fat diet by increasing the PRL concentration in mouse serum, activating the JAK2/STAT5 signaling pathway mediated by PRL-PRLR [30]. GH can inhibit the apoptosis of mammary epithelial cells in the late lactation period and stimulate the production of IGF-1. The GH and IGF-1 systems regulate the proliferation and differentiation of mammary epithelial cells, promoting mammary gland development and milk synthesis [31,32]. Research has shown that feeding soybean isoflavone and astragalus polysaccharides to sows in late pregnancy increases their total milk production and significantly elevates their blood GH and IGF-1 levels [33]. Fenugreek modulates the GH/IGF-1 axis to prolong the lactation durations and milk yields of rats [34]. Our findings align with those of previous studies, revealing elevated PRL, GH, and IGF-1 levels in the sera of lactating sows and a significant increase in the weaning weights of piglets. These findings indicate that the addition of AS alone significantly enhances sow milk production and plays a crucial role in piglet growth.

The immune system of neonatal piglets is not fully developed, and maternal milk is one of the primary pathways through which they acquire immune competence [35]. Maternal milk immunoglobulins provide passive immune protection to offspring and regulate offspring growth, development, and intestinal health through various pathways [36]. The IgA levels in the intestine are closely associated with the SIgA levels, preventing intestinal diseases in offspring [37,38]. In this study, when sows were fed 0.5% ASE, the IgA and IgG levels in their milk increased, and the T3, T4, GH, and IgG levels in the piglet blood significantly increased. These findings indicate that lactating sows that consume AS have increased lactation and immunity, which play crucial roles in the growth, development, and immune levels of piglets.

Blood metabolomics can reflect an animal’s metabolic status after feeding and the effects of metabolites on the body. Postpartum sows may experience mastitis and metritis, affecting piglet growth [39]. The anti-inflammatory effects of the active compounds in AS may increase sow milk production. In this study, lactating sows fed AS had significantly increased concentrations of nine AS metabolites in their blood, including coniferaldehyde, andrographin, and plantamajoside. Studies have demonstrated that supplementing sow diets with Andrographis during late gestation and lactation significantly enhances their colostrum yields and the IgG concentration in their milk [40]. These findings align with our experimental results, which showed that AS dietary supplementation significantly increased the serum andrographin levels and elevated the IgG content in the sow milk. Coniferaldehyde exerts anti-inflammatory effects by inhibiting the activation of TAK1-mediated MAP kinase/NF-κB, upregulating the AMPK signaling pathway, and increasing antioxidant enzyme expression through the Nrf2 signaling pathway [41]. Plantamajoside exerts anti-inflammatory effects by inhibiting the production of IL-1β, IL-6, and TNF-α through the MAPK and NF-κB pathways [42]. This finding is consistent with the results of the present study, which demonstrated that AS significantly increased the serum PMS levels and reduced the IL-6 and TNF-α expression in the sows. Additionally, plantamajoside displays antiapoptotic effects by reducing caspase-3 protein expression [43]. During late lactation, glandular cells undergo apoptosis, leading to mammary tissue degeneration and a decreased lactation capacity [44]. The active compounds in AS may delay mammary tissue degeneration by inhibiting the apoptosis of mammary epithelial cells, thereby maintaining the lactation capacity in late lactation. Although we have discussed several potential mechanisms by which metabolites promote lactation, the limited sample size of our study restricts the scope of these interpretations. Further experimental studies are needed to elucidate the underlying mechanisms in more detail.

The intestinal tract is a crucial tissue for nutrient absorption in the body, with the intestinal microbiota playing a key role in regulating physiological parameters and maintaining overall health [45,46]. Herbal compound formulas or extracts can restore the intestinal ecological balance to some extent by altering the intestinal microbiota composition, promoting an increase in beneficial bacteria, decreasing harmful bacteria, and improving the host’s metabolism and immune function. The combined use of ingredients such as Astragalus and licorice can modulate the microbial community in the intestine through various mechanisms, increasing the abundance of beneficial bacteria, inhibiting potential pathogens, and thereby promoting intestinal health [47]. In this study, after consuming AS, the sows presented increased abundances of microbial groups such as the *Christensenellaceae_R_7_group*, *Lactobacillus*, and the *Lachnospiraceae_AC2044_group*. The *Christensenellaceae_R_7_group* is a microbial group that is associated with mammary health and may play a crucial role in maintaining it [48]. Furthermore, supplementation with Panax notoginseng increased the abundance of the *Christensenellaceae_R-7_group*, which is recognized as a microbial population of significant importance to host health, in the porcine cecum [49]. A study by Liu [50] demonstrated a negative linear correlation between the abundance of the *Lachnospiraceae_AC2044_group* in the porcine intestine and TNF-α levels. *Olsenella* is considered a potential biomarker of inflammatory disorders of the breast [51]. Dietary resveratrol supplementation in farrowing sows reduced the relative abundance of *UCG-008* in their feces, which was positively linearly correlated with the expression of IL-1β and IL-6 [52]. In this study, we found that dietary supplementation with AS increased the abundance of the *Lachnospiraceae_AC2044_group* and decreased the abundance of *UCG-008* in the sow intestines and significantly reduced the serum TNF-α and IL-6 levels. These findings are consistent with those of previous studies. In conclusion, AS may promote lactation in sows by modulating the gut microbiota, although the underlying mechanisms require further investigation.

Intestinal health plays a crucial role in the growth, development, and immunity of suckling piglets [53]. The villus height–crypt depth ratio in the small intestine is an important indicator of the digestive and absorptive capacity [54]. Feeding AS blood-tonifying soup to immunocompromised mice restored the villus-crypt ratio in the intestines, enhancing the intestinal barrier function [55]. Moreover, the consumption of resveratrol by sows increased the VH–crypt depth ratios in the jejuna and ilea of their offspring [56]. Our research revealed that the sow AS intake significantly increased the villus height-crypt depth ratios in the piglets’ small intestines, facilitating the better absorption and utilization of nutrients and thereby promoting their growth and development. The immature development of the intestinal mucosal system in suckling piglets is a significant factor leading to diarrhea and mortality [57]. SIgA is the primary immunoglobulin for mucosal secretion, acting as the first line of defense against harmful substances invading the intestinal epithelium [58,59]. Maternal antibiotic exposure can reduce milk IgA levels, subsequently decreasing intestinal SIgA in offspring [60]. In our study, the sow intake of AS led to a significant increase in the SIgA contents in the intestines of the piglets, possibly due to the elevated IgA levels in their milk, thereby promoting their healthy intestinal development. DAO and D-LA are common biomarkers for assessing intestinal permeability [61,62]. DAO is an enzyme in intestinal mucosal cells that is released into the bloodstream when the intestinal mucosa is damaged, leading to elevated serum DAO levels [63]. D-LA is a metabolic byproduct of intestinal bacteria; increased blood D-LA levels occur when the intestinal mucosal barrier is compromised [64]. In our study, piglets in the AS group presented significantly reduced serum DAO and D-LA levels and significantly increased mRNA levels of Occludin, Claudin-1, and ZO-1 in their intestinal tissues, indicating that the maternal intake of AS can enhance intestinal barrier function, reduce intestinal permeability, promote intestinal health, and increase disease resistance. We cannot determine whether certain bioactive AS metabolites or other components in milk contribute to enhanced growth and intestinal barrier functioning in piglets on the basis of this study. Therefore, further research is needed to elucidate the specific components and mechanisms involved in this process.

## 5. Conclusions

The supplementation of 0.5% AS promoted lactogenesis in sows by leveraging the synergistic effects of metabolites and intestinal flora, ultimately facilitating the holistic enhancement of their lactation performances and the growth, development, and immune health of their offspring. These findings support the use of Chinese herbal medicine in pig production, although further research is needed to clarify the underlying mechanisms and effects on milk composition.

## Figures and Tables

**Table 1:** Sow diet formula and nutritional composition.

Ingredient	Value
Corn (%)	51.12
Soybean meal (%)	24.61
Wheat bran (%)	4.00
Rapeseed meal (%)	2.50
Rice bran (%)	5.00
Beef tallow (%)	6.05
Molasses (%)	3.50
CaHPO_4_ (%)	1.64
Limestone (%)	0.76
NaCl (%)	0.50
Lysine salt (98%) (%)	0.12
Vitamin and mineral premix (%)	0.20
Nutrient Values (Calculated)	
Metabolizable energy (MJ/kg)	3.44
Crude protein (%)	17.10
Crude fat (%)	9.10
Lysine (%)	1.00
Ca (%)	0.85
P (%)	0.73

Note: The premix provided the following per kilogram of the diet: vitamin A, 20,000 IU; vitamin D, 4000 IU; vitamin E, 50 mg; vitamin K_3_, 0.3 mg; vitamin B_1_, 2 mg; vitamin B_2_, 8 mg; vitamin B_6_, 3 mg; vitamin B_12_, 0.03 mg; nicotinic acid, 20 mg; pantothenic acid, 15 mg; folic acid, 1.2 mg; biotin, 0.17 mg; Cu, 15 mg; Fe, 70 mg; Mn, 20 mg; Zn, 50 mg; I, 0.3 mg; and Se, 0.5 mg.

**Table 2:** Primer sequences used in this study.

Primer Name	Forward (5′-3’)	Reverse (5’-3’)	Product Length (bp)
18S	CATGCATGTCTAAGTACGCACGG	AGGCTGACCGGGTTGGTTTTGAT	200
Claudin-1	CTTCTGGGTTTCATCCTGGCTTCG	CCTGAGCAGTCACGATGTTGTCC	194
Occludin	CAACGGCAAAGTGAATGGCAAGAC	TCATCCACGGACAAGGTCAGAGG	188
ZO-1	GCCAAGCCAGTCCATTCTCAGAG	TCCATAGCATCAGTTTCGGGTTTCC	185

ZO-1 = Zonula Occludens Protein 1.

**Table 3:** Potential bioactive components of AS in sow sera.

MS2 Name	Formula	MZ	RT	Type	*p*-Value	LOG_FOLDCHANGE
2′,4′,6′-Trihydroxyacetophenone	C_8_H_8_O_4_	149.02	304.00	NEG	0.0053	11.51
Coniferaldehyde	C_10_H_10_O_3_	177.06	305.30	NEG	0.0075	9.91
Andrograpanin	C_20_H_30_O_3_	341.21	418.10	POS	0.0091	2.25
5-[6-(3-hydroxy-4-methoxyphenyl)-1,3,3a,4,6,6a-hexahydrofuro [3,4-c]furan-3-yl]-2-methoxyphenol	C_20_H_22_O_6_	357.13	404.30	NEG	0.0109	7.88
Gemichalcone_C	C_30_H_28_O_9_	533.18	345.00	POS	0.0159	3.35
Plantamajoside	C_29_H_36_O_16_	639.19	354.60	NEG	0.0173	7.15
4-Hydroxybenzyl alcohol	C_7_H_8_O_2_	123.05	248.30	NEG	0.0380	2.12
Dillapiole	C_12_H_14_O_4_	223.10	353.60	POS	0.0391	4.67
4-hydroxy-3-(3-methylbut-2-enyl)benzoic acid	C_12_H_14_O_3_	205.09	382.70	NEG	0.0421	9.78

MS2 Name: substances identified through qualitative matching analysis with secondary mass spectrometry; MZ: the mass-to-charge ratio of the ion is a material characteristic; RT: the retention time of the substance in chromatography.

**Table 4:** Impact of sow dietary inclusion of AS on piglet growth performance during the lactation period.

Item	CON	0.5% ASE	1% ASE	SEM	*p*-Value
Initial weight at day 5, kg	1.83	1.83	1.81	0.02	0.8883
Weight at day 10, kg	2.80	2.88	2.87	0.04	0.6694
Weight at day 15, kg	4.81 ^b^	5.33 ^a^	5.16 ^ab^	0.06	0.0675
Weaning weight at day 21, kg	5.02 ^b^	5.58 ^a^	5.39 ^ab^	0.10	0.0377
Average daily gain, g/d	199.41 ^b^	234.63 ^a^	224.15 ^ab^	5.97	0.0354

^a,b^ In the same row, values without letters or with the same letters in superscripts mean no significant differences (*p* > 0.05), while those with different small letters in superscripts mean significant differences (*p* < 0.05).

## Data Availability

The data presented in this study are available on request from the corresponding author.

## Supplementary Materials

The following supporting information can be downloaded at: https://www.mdpi.com/article/10.3390/vetsci12040370/s1, Table S1: AS Metabolites; Table S2: Differentially upregulated metabolites; Table S3: Differentially down-regulated metabolites.

## Abbreviations

The following abbreviations are used in this manuscript:

DAO	Diamine oxidase
D-LA	D-Lactate
GH	Growth Hormone
IgA	Immunoglobulin A
IGF-1	Insulin-like growth factor 1
IgG	Immunoglobulin G
IgM	Immunoglobulin M
IL-6	Interleukin-6
OTUs	Operational Taxonomic Units
PRL	Prolactin
SIgA	Secretory immunoglobulin A
T3	Triiodothyronine
T4	Thyroxine
TIC	Time–intensity curve
TNF-α	Tumor necrosis factor-α
ZO-1	Zonula Occludens Protein 1
