# Supplementary material for: Dietary Angelica sinensis Enhances Sow Lactation and Piglet Development Through Gut Microbiota and Metabolism
Source: Vet Sci. 2025 Apr 15;12(4):370. doi: 10.3390/vetsci12040370 (PMC12030784; doi:10.3390/vetsci12040370)
Supplement: Supplementary file 1 [file vetsci-12-00370-s001.zip › Supplementary Table 1.pdf]

Supplementary Table 1. AS Metabolites

Name	Formula	MZ	RT	Type
Isoscopoletin	C10H8O4	191.03	286.10	NEG
Sedanolide	C12H18O2	195.14	433.20	POS
5,9-dimethyltetracyclo[11.2.1.01,10.04,9]hexadecane-5,14-dicarboxylic acid	C20H30O4	333.21	459.20	NEG
2-hydroxy-5-[(2S,3R,4S,5S,6R)-3,4,5-trihydroxy-6-(hydroxymethyl)tetrahydropyran-2-yl]oxy-benzoic acid	C13H16O9	315.07	114.40	NEG
cis-Jasmone	C11H16O	165.13	409.80	POS
Hydroxyacetone	C3H6O2	73.03	61.20	NEG
(2R,3R,4S,5S,6R)-2-benzyloxy-6-[[(2R,3R,4R)-3,4-dihydroxy-4-(hydroxymethyl)tetrahydrofuran-2-yl]oxymethyl]tetrahydropyran-3,4,5-triol	C18H26O10	401.14	239.70	NEG
2',4',6'-Trihydroxyacetophenone	C8H8O4	149.02	304.00	NEG
3,4-Dihydroxyphenylglycol	C8H10O4	151.04	74.40	NEG
4-hydroxy-3-(3-methylbut-2-enyl)benzoic acid	C12H14O3	205.09	382.70	NEG
D-Malate	C4H6O5	133.01	435.60	NEG
Dimethyl phthalate	C10H10O4	177.05	330.20	POS
(2R,3R,4S,5S,6R)-2-benzyloxy-6-[[(2S,3R,4S,5R)-3,4,5-trihydroxytetrahydropyran-2-yl]oxymethyl]tetrahydropyran-3,4,5-triol	C18H26O10	401.14	239.70	NEG
Coniferaldehyde	C10H10O3	177.06	305.30	NEG
(3S,4S)-3-hydroxy-4-[(4-hydroxy-3-methoxy-phenyl)methyl]-3-[[3-methoxy-4-[(2S,3R,4S,5S,6R)-3,4,5-trihydroxy-6-(hydroxymethyl)tetrahydropyran-2-yl]oxy-phenyl]methyl]tetrahydrofuran-2-one	C26H32O12	535.18	275.80	NEG
Pimpinellin	C13H10O5	247.06	363.00	POS
trans-Ferulic acid	C10H10O4	193.05	323.40	NEG
[(1S,6R,10S,13S,14R,15R)-1,6-dihydroxy-8-(hydroxymethyl)-4,12,12,15-tetramethyl-14-[(E)-2-methylbut-2-enoyl]oxy-5-oxo-13-tetracyclo[8.5.0.02,6.011,13]pentadeca-3,8-dienyl] 2-methylbutanoate	C30H42O8	529.28	456.20	NEG
4-hydroxy-1H-indole-3-carbaldehyde	C9H7NO2	162.05	278.70	POS
5-[2-(3-furyl)ethyl]-8-hydroxy-5,6,8a-trimethyl-3,4,4a,6,7,8-hexahydronaphthalene-1-carboxylic acid	C20H28O4	331.19	460.70	NEG
Isoferulic acid	C10H10O4	193.05	323.40	NEG
(2R,3R,4S,5S,6R)-2-benzyloxy-6-[[(2S,3R,4S,5S)-3,4,5-trihydroxytetrahydropyran-2-yl]oxymethyl]tetrahydropyran-3,4,5-triol	C18H26O10	401.14	239.70	NEG
(2S,3R,4S,5S,6R)-2-[4-[(2R,3R)-4-hydroxy-3-[(4-hydroxy-3-methoxy-phenyl)methyl]-2-(hydroxymethyl)butyl]-2-methoxy-phenoxy]-6-(hydroxymethyl)tetrahydropyran-3,4,5-triol	C26H36O11	523.22	272.00	NEG
Anthranilic acid (Vitamin L1)	C7H7NO2	136.04	285.60	NEG
Gramine	C11H14N2	175.12	213.30	POS
(-)-Camphoric acid	C10H16O4	199.10	295.70	NEG
7-hydroxy-3-(4-hydroxyphenyl)-5-methoxy-chromen-4-one	C16H12O5	285.08	335.70	POS
(2Z,4E)-5-[(1R,3R,5S,8S)-3,8-dihydroxy-1,5-dimethyl-6-oxabicyclo[3.2.1]octan-8-yl]-3-methyl-penta-2,4-dienoic acid	C15H22O5	281.14	267.20	NEG

7,8-dihydroxy-4-phenyl-chromen-2-one	C15H10O4	253.05	341.80	NEG
Sebacic acid	C10H18O4	183.10	357.40	NEG
3-Hydroxycinnamic acid	C9H8O3	163.04	298.50	NEG
2-(1-carboxyethyl)-5-methyl-cyclopentanecarboxylic acid	C10H16O4	199.10	295.70	NEG
1-(2,6-dihydroxyphenyl)-9-phenyl-nonan-1-one	C21H26O3	325.18	553.30	NEG
[(2S,3R,4S,5S,6R)-3,4,5-trihydroxy-6-(hydroxymethyl)oxan-2-yl] 4-prop-1-en-2-ylcyclohexene-1-carboxylate	C16H24O7	309.13	371.30	NEG
1-(4-hydroxy-3,5-dimethoxy-phenyl)propan-1-one	C11H14O4	193.09	344.80	POS
4-Chromanone	C9H8O2	149.06	303.00	POS
6,7-dimethoxy-3-(4-methoxyphenyl)chromen-4-one	C18H16O5	313.11	322.10	POS
Methyl caffeate	C10H10O4	193.05	323.40	NEG
Nicotinate	C6H5NO2	124.04	65.30	POS
Isonicotinic acid	C6H5NO2	124.04	65.30	POS
Pinoresinol	C20H22O6	357.13	333.20	NEG
Pyridoxal (Vitamin B6)	C8H9NO3	150.06	65.50	POS
4-hydroxy-5-[(2S,3R,4S,5S,6R)-3,4,5-trihydroxy-6-(hydroxymethyl)tetrahydropyran-2-yl]oxy-1H-benzo[f]isobenzofuran-3-one	C18H18O9	377.09	290.10	NEG
4-Hydroxybenzyl alcohol	C7H8O2	123.05	248.30	NEG
2,4-bis(3-methylbut-2-enyl)-6a,11a-dihydro-6H-benzofuro[3,2-c]chromene-3,9-diol	C25H28O4	391.19	422.50	NEG
Marrubiin	C20H28O4	331.19	460.70	NEG
1-Methyltryptamine	C11H14N2	175.12	213.30	POS
(2S)-6-Prenylnaringenin	C20H20O5	341.14	403.30	POS
2-[[3,4-dihydroxy-4-(hydroxymethyl)tetrahydrofuran-2-yl]oxymethyl]-6-(5-hydroxy-1,7,7-trimethyl-norbornan-2-yl)oxy-tetrahydropyran-3,4,5-triol	C21H36O11	463.22	311.20	NEG
(E)-Ethyl p-methoxycinnamate	C12H14O3	189.09	454.70	POS
2-methyl-3-[(2S,3R,4S,5S,6R)-3,4,5-trihydroxy-6-(hydroxymethyl)tetrahydropyran-2-yl]oxy-pyran-4-one	C12H16O8	289.09	187.50	POS
Glucovanillin	C14H18O8	313.09	215.90	NEG
methyl (E)-2-[(2S,3R,12bR)-3-vinyl-1,2,3,4,6,7,12,12b-octahydroindolo[2,3-a]quinolizin-2-yl]-3-methoxy-prop-2-enoate	C22H26N2O3	367.20	249.20	POS
2,4,5-Trimethoxybenzaldehyde	C10H12O4	195.07	299.30	NEG
6,7-Dihydroxy-4-phenylcoumarin	C15H10O4	253.05	288.80	NEG
Gastrodin	C13H18O7	267.09	317.50	NEG
Irisflorentin	C20H18O8	387.11	285.10	POS
(-)-Tylocrebrine	C24H27NO4	394.20	437.30	POS
APIOLE	C12H14O4	223.10	329.90	POS
Cuminaldehyde	C10H12O	149.10	240.70	POS
Ethanone,_1-[4-[(6-O-beta-D-xylopyranosyl-beta-D-glucopyranosyl)oxy]phenyl]-	C19H26O11	431.15	272.40	POS

ORSELLINIC ACID, ETHYL ESTER	C10H12O4	197.08	258.10	POS
2'-Hydroxy-4',6'-dimethoxy-3'-methylacetophenone	C11H14O4	211.10	310.30	POS
Artemetin	C20H20O8	387.11	312.00	NEG
Demethoxycurcumin	C20H18O5	339.12	290.30	POS
Ethyl caffeate	C11H12O4	207.07	363.60	NEG
(E)-3-[4-[(2S,3R,4S,5S,6R)-3,4,5-trihydroxy-6-(hydroxymethyl)oxan-2-yl]oxyphenyl]prop-2-enoic acid	C15H18O8	344.13	236.00	POS
3-Butylidenephthalide	C12H12O2	189.09	312.60	POS
3,4,5-Trimethoxycinnamic acid	C12H14O5	239.09	280.80	POS
5-[6-(3-hydroxy-4-methoxyphenyl)-1,3,3a,4,6,6a-hexahydrofuro[3,4-c]furan-3-yl]-2-methoxyphenol	C20H22O6	357.13	404.30	NEG
andrograpanin	C20H30O3	341.21	418.10	POS
Calealactone B	C21H26O9	421.15	359.30	NEG
Dillapiole	C12H14O4	223.10	353.60	POS
Eupatoriochromene	C13H14O3	219.10	365.60	POS
lycorine	C16H17NO4	310.11	329.00	POS
arborinine	C16H15NO4	308.09	269.40	POS
Ferutinin	C22H30O4	357.21	422.60	NEG
Toxol	C13H14O3	219.10	418.10	POS
4-Hydroxyphenethyl alcohol	C8H10O2	137.06	332.90	NEG
2,4-Dimethylcinnamic acid	C11H12O2	175.08	308.00	NEG
Flavone base + 4O, 1Prenyl	C20H18O6	353.10	321.60	NEG
Pentose-Hexose + C10H17	C21H36O10	447.22	400.20	NEG
Pristimerin	C30H40O4	463.28	570.90	NEG
Senkyunolide A	C12H16O2	193.12	413.60	POS
[6]-Gingerol	C17H26O4	295.19	415.80	POS
Cafestol	C20H28O3	339.19	232.10	POS
gossypol	C30H30O8	519.20	386.40	POS
Pinoresinol 4-O-glucoside	C26H32O11	538.23	276.70	POS
Piperonyl butoxide	C19H30O5	337.20	442.90	NEG
xi-2,2,6-Trimethyl-1,4-cyclohexanedione	C9H14O2	155.11	415.10	POS
Chalcone base + 3O, 1Prenyl	C20H20O4	323.13	394.00	NEG
Gallocatechin	C15H14O7	305.07	268.40	NEG
Brucine	C23H26N2O4	395.20	441.80	POS
2,5-Octadien-1-ol	C8H14O	127.11	29.40	POS
Licarin A	C20H22O4	327.16	309.10	POS

Celastrol	C29H38O4	449.27	556.50	NEG
Mesembrinol	C17H25NO3	292.19	371.60	POS
Bergenin	C14H16O9	327.07	229.30	NEG
7-methoxy-6-(1,2,3-trihydroxy-3-methylbutyl)chromen-2-one	C15H18O6	317.09	363.40	POS
Bixin	C25H30O4	393.20	441.20	NEG
Phytolaccagenin	C31H48O7	531.33	473.40	NEG
ginkgolide B	C20H24O10	423.13	357.50	NEG
(2S,3R,4S,5S,6R)-2-[4-[[(3S,4R,5S)-3-hydroxy-5-(4-hydroxy-3-methoxyphenyl)-4-(hydroxymethyl)oxolan-3-yl]methyl]-2-methoxyphenoxy]-6-(hydroxymethyl)oxane-3,4,5-triol	C26H34O12	556.24	264.50	POS
3-Hydroxy-carbofuran	C12H15NO4	238.11	307.40	POS
(5E,9E)-Farnesylacetone	C18H30O	263.24	528.40	POS
Erianin	C18H22O5	341.13	292.70	POS
(2S,3R,4S,5S,6R)-2-[3-hydroxy-5-[(Z)-2-(4-hydroxyphenyl)ethenyl]phenoxy]-6-(hydroxymethyl)oxane-3,4,5-triol	C20H22O8	389.12	330.20	NEG
(E)-Resveratroloside	C20H22O9	424.16	330.10	POS
Humulone	C21H30O5	361.20	469.80	NEG
Strychnine_N_Oxide	C21H22N2O3	351.17	231.10	POS
Dimethyl (R)-(+)-malate	C6H10O5	163.06	79.80	POS
Plantamajoside	C29H36O16	639.19	354.60	NEG
(R)-2',4',7-Trihydroxy-3',8-diprenylisoflavan	C25H30O4	395.22	447.10	POS
Anacardic acid diene	C22H32O3	343.23	439.20	NEG
.beta.-D-Glucopyranoside, 2-methoxy-4-[[rel-(1R,3aS,4R,6aS)]-tetrahydro-4-(4-hydroxy-3-methoxyphenyl)-1H,3H-furo[3,4-c]furan-1-yl]phenyl	C26H32O11	519.19	287.90	NEG
Deoxyloganic acid (Not validated)	C16H24O9	359.14	290.90	NEG
Arnebinol	C16H20O2	245.15	432.80	POS
Ginkgolide A	C20H24O9	426.18	327.20	POS
Phenethyl rutinoside	C20H30O10	429.18	260.00	NEG
Gemichalcone_C	C30H28O9	533.18	345.00	POS
Tulipinolide	C17H22O4	308.19	421.90	POS
[1a-(hydroxymethyl)-2-[3,4,5-trihydroxy-6-(hydroxymethyl)oxan-2-yl]oxy-2,5a,6,6a-tetrahydro-1bH-oxireno[5,6]cyclopenta[1,3-c]pyran-6-yl] 3,4-dimethoxybenzoate	C24H30O13	544.20	233.40	POS
Alnuside A	C24H30O9	461.18	409.20	NEG
Corchoionoside_B	C19H28O9	401.18	332.00	POS
Apiosylrhododendrin	C21H32O11	459.19	362.40	NEG
4-Hydroxy-3-methyl-9,10-dioxo-9,10-dihydroanthracen-2-yl 6-O-.beta.-D-xylopyranosyl-.beta.-D-glucopyranoside	C26H28O13	547.15	287.30	NEG

6-[2-(1,3-benzodioxol-5-yl)ethyl]-4-methoxypyran-2-one	C15H14O5	292.12	400.10	POS
Gelsemicine	C20H26N2O4	359.20	457.70	POS
rescinnamine	C35H42N2O9	633.28	308.30	NEG
Alpha-Ergocryptine	C32H41N5O5	496.34	486.90	POS
Lappaconitine	C32H44N2O8	583.30	381.50	NEG
Launobine	C18H17NO4	312.13	330.60	POS
Protostemonine	C23H31NO6	418.22	352.70	POS
Asperglaucide	C27H28N2O4	445.22	234.10	POS
4-[5-(4-hydroxy-3-methoxyphenyl)-3,4-dimethyloxolan-2-yl]-2-methoxyphenol	C20H24O5	383.13	296.10	POS
Genipin-gentiobioside	C23H34O15	549.18	389.40	NEG
Cyclomorusin	C25H22O6	419.14	186.10	POS
[(2R,3S,4S,5R,6R)-6-[(2S,3S,4S,5R)-3,4-dihydroxy-2,5-bis(hydroxymethyl)oxolan-2-yl]oxy-3,4,5-trihydroxyoxan-2-yl]methyl (E)-3-(4-hydroxy-3,5-dimethoxyphenyl)prop-2-enoate	C23H32O15	549.17	370.30	POS
5-[2-(3,4-dihydroxy-2,5-dimethoxyoxolan-3-yl)ethyl]-1,4a-dimethyl-6-methylidene-3,4,5,7,8,8a-hexahydro-2H-naphthalene-1-carboxylic acid	C22H36O7	411.24	392.60	NEG
Alanine	C3H7NO2	88.04	44.60	NEG
cis-11.14-Eicosadienoic acid	C20H36O2	307.26	574.10	NEG
cis-8,11,14-Eicosatrienoic acid	C20H34O2	305.25	559.20	NEG
Citrulline	C6H13N3O3	174.09	45.30	NEG
DL-Threonine	C4H9NO3	118.05	44.40	NEG
Indole-3-carboxylic acid	C9H7NO2	160.04	312.20	NEG
L-Histidine	C6H9N3O2	154.06	43.60	NEG
Sarcosine	C3H7NO2	88.04	44.60	NEG
Succinic Acid	C4H6O4	117.02	81.70	NEG
Taurine	C2H7NO3S	124.01	43.60	NEG
trans-11-Eicosenoic acid	C20H38O2	309.28	590.30	NEG
Valine	C5H11NO2	116.07	49.10	NEG
16-Hydroxypalmitic acid	C16H32O3	271.23	478.30	NEG
2-Amino-5-ureidopentanoic acid	C6H13N3O3	174.09	45.30	NEG
2-Aminobutyric acid	C4H9NO2	102.06	44.90	NEG
2-Hydroxydecanoic-acid	C10H20O3	187.13	432.60	NEG
2-Hydroxyisobutyric acid	C4H8O3	85.03	82.60	NEG
3-(4-Methoxyphenyl)propanoic acid	C10H12O3	179.07	336.80	NEG
alpha-Linolenic acid	C18H30O2	277.22	535.80	NEG

beta-Alanine	C3H7NO2	88.04	44.60	NEG
Chlorogenic acid	C16H18O9	355.10	237.70	POS
cis-9-Palmitoleic acid	C16H30O2	253.22	548.40	NEG
Continentalic acid	C20H30O2	301.22	546.80	NEG
D-Proline	C5H9NO2	114.06	45.30	NEG
Dimethylglycine	C4H9NO2	102.06	44.90	NEG
Fraxinol	C11H10O5	223.06	281.80	POS
Indigo	C16H10N2O2	263.08	406.30	POS
Myristic acid	C14H28O2	227.20	544.80	NEG
Oleic acid	C18H34O2	281.25	571.50	NEG
Ornithine	C5H12N2O2	133.10	39.50	POS
Pelargonic acid	C9H18O2	157.12	456.00	NEG
Proline	C5H9NO2	114.06	45.30	NEG
Propionic acid	C3H6O2	73.03	117.30	NEG
Royal jelly acid	C10H18O3	185.12	346.20	NEG
trans-Vaccenic acid	C18H34O2	281.25	571.50	NEG
Undecanoic acid	C11H22O2	185.15	496.50	NEG
Vanillin	C8H8O3	151.04	274.90	NEG
(1S,2S,6Z,10S,11S,16Z)-6,16-di(butylidene)-5,15-dioxapentacyclo[9.5.2.01,13.02,10.03,7]octadeca-3(7),12-diene-4,14-dione	C24H28O4	381.21	468.70	POS
(5R,9S)-5,9-dimethyl-14-methylene-tetracyclo[11.2.1.01,10.04,9]hexadecane-5-carboxylic acid	C20H30O2	301.22	546.80	NEG
1,4a,7-trimethyl-7-vinyl-3,4,6,8,8a,9,10,10a-octahydro-2H-phenanthrene-1-carboxylic acid	C20H30O2	301.22	546.80	NEG
10-Undecenoic acid	C11H20O2	183.14	473.90	NEG
3-methoxy-4-[3,4,5-trihydroxy-6-(hydroxymethyl)tetrahydropyran-2-yl]oxy-benzoic acid	C14H18O9	329.09	204.10	NEG
4-Guanidinobutyric acid	C5H11N3O2	146.09	48.60	POS
4,5-Dicaffeoylquinic acid	C25H24O12	517.13	300.20	POS
5-Hydroxymethylfurfural	C6H6O3	127.04	101.80	POS
7-Ketolithocholic acid	C24H38O4	389.27	431.00	NEG
Capric acid	C10H20O2	171.14	477.20	NEG
Creatine	C4H9N3O2	132.08	45.80	POS
Cryptochlorogenic acid	C16H18O9	355.10	237.70	POS
D-Pantothenic Acid	C9H17NO5	220.12	189.90	POS
Dihydroxyacetone	C3H6O3	89.02	52.10	NEG
Docosapentaenoic acid (DPA)	C22H34O2	329.25	549.00	NEG
Dodecanedioic acid	C12H22O4	229.14	400.80	NEG

ethyl octanoate	C10H20O2	171.14	477.20	NEG
Galactose	C6H12O6	203.05	44.00	POS
gamma-Glutamylphenylalanine	C14H18N2O5	293.11	225.30	NEG
Glutamine	C5H10N2O3	147.08	46.00	POS
Guanosine	C10H13N5O5	284.10	105.50	POS
H-D-Trp-OH	C11H12N2O2	203.08	207.60	NEG
Hippuric acid	C9H9NO3	162.05	240.50	POS
Hydroxyphenylacetylglycine	C10H11NO4	208.06	147.40	NEG
Hydroxytyrosol	C8H10O3	135.05	215.30	NEG
Indoleacetic acid	C10H9NO2	174.06	311.70	NEG
Inosine	C10H12N4O5	269.09	100.20	POS
Kaurenoic acid	C20H30O2	301.22	546.80	NEG
Kojic acid	C6H6O4	143.03	101.10	POS
L-Arginine	C6H14N4O2	175.12	44.30	POS
Linoleic acid	C18H32O2	279.23	553.30	NEG
Naringenin chalcone	C15H12O5	271.06	352.60	NEG
Nonadecanoic acid	C19H38O2	297.28	600.90	NEG
Norleucine	C6H13NO2	130.09	71.10	NEG
Oleamide	C18H35NO	282.28	551.60	POS
p-Synephrine	C9H13NO2	150.09	52.60	POS
Palmitamide	C16H33NO	256.26	548.50	POS
Rutaecarpine	C18H13N3O	270.10	423.00	POS
Stearidonic acid	C18H28O2	275.20	520.80	NEG
Threonine	C4H9NO3	120.07	46.00	POS
(E)-5-(1,2,4a,5-tetramethyl-2,3,4,7,8,8a-hexahydronaphthalen-1-yl)-3-methyl-pent-2-enoic acid	C20H32O2	303.23	547.40	NEG
(Z)-9,10,11-trihydroxyoctadec-12-enoic acid	C18H34O5	329.23	409.80	NEG
13(S)-HODE	C18H32O3	295.23	486.60	NEG
2,4-Dihydroxybutanoic acid	C4H8O4	119.03	48.50	NEG
3-hydroxybenzaldehyde	C7H6O2	121.03	273.60	NEG
3-Hydroxybenzoic acid	C7H6O3	137.02	253.90	NEG
4-Aminobutyric acid (GABA)	C4H9NO2	102.06	44.90	NEG
9-Oxooctadecanoic acid	C18H34O3	297.24	517.50	NEG
Acetylcarnitine (Car(2:0))	C9H17NO4	204.12	48.40	POS
Adenine	C5H5N5	134.05	78.40	NEG

Adipic acid	C6H10O4	145.05	215.70	NEG
Allose	C6H12O6	203.05	44.00	POS
Angeolide	C24H28O4	381.21	443.40	POS
Butein	C15H12O5	271.06	352.60	NEG
Carnitine	C7H15NO3	162.11	45.40	POS
Catechin	C15H14O6	289.07	243.10	NEG
cis-11-Eicosenoic acid	C20H38O2	309.28	590.30	NEG
D-Glutamine	C5H10N2O3	147.08	46.00	POS
Dihydroferulic acid	C10H12O4	195.07	283.70	NEG
Dodecanoic acid	C12H24O2	199.17	514.20	NEG
Eicosapentaenoic acid	C20H30O2	301.22	531.00	NEG
Fructose	C6H12O6	203.05	44.00	POS
gamma-Linolenic acid	C18H30O2	277.22	535.80	NEG
Glyceraldehyde	C3H6O3	89.02	52.10	NEG
Heptadecanoic acid	C17H34O2	269.25	580.40	NEG
hexyl hexanoate	C12H24O2	199.17	514.20	NEG
Homoserine	C4H9NO3	118.05	44.40	NEG
Hydroquinone	C6H6O2	109.03	229.40	NEG
Indole-3-propionic acid	C11H11NO2	190.09	348.10	POS
Kynurenine	C10H12N2O3	209.09	105.50	POS
L-allo-isoleucine	C6H13NO2	130.09	283.70	NEG
Leucine	C6H13NO2	132.10	73.20	POS
Levulinic acid	C5H8O3	115.04	96.30	NEG
LPC(16:0)	C24H50NO7P	496.34	515.80	POS
Lysine	C6H14N2O2	147.11	39.50	POS
myo-Inositol	C6H12O6	179.06	43.20	NEG
N-Acetyltryptophan	C13H14N2O3	247.11	291.90	POS
N2-Acetylornithine	C7H14N2O3	173.09	47.60	NEG
Pentadecanoic acid	C15H30O2	241.22	557.80	NEG
Pimelic acid	C7H12O4	159.07	256.20	NEG
Pyrocatechol	C6H6O2	109.03	229.40	NEG
Pyrrolidine	C4H9N	72.08	48.70	POS
Synephrine	C9H13NO2	150.09	52.60	POS
Tagatose	C6H12O6	203.05	44.00	POS

Xanthine	C5H4N4O2	151.03	88.20	NEG
(1S,3R,4R,5R)-1,3,4-trihydroxy-5-[(E)-3-(3-hydroxy-4-methoxy-phenyl)prop-2-enoyl]oxy-cyclohexanecarboxylic acid	C17H20O9	367.10	260.10	NEG
1-phenylbutane-1,3-dione	C10H10O2	163.08	461.50	POS
1,4-Dicaffeoylquinic acid	C25H24O12	499.12	295.50	POS
2-Aminoisobutyric acid	C4H9NO2	102.06	44.90	NEG
2-Hydroxy-3-(4-hydroxyphenyl)propanoic acid	C9H10O4	181.05	214.70	NEG
3,7-Di-O-methylquercetin	C17H14O7	329.07	383.70	NEG
3beta-Hydroxy-5-cholestenoic acid (3-HCOA)	C27H44O3	415.32	533.90	NEG
5,6,7-Trimethoxycoumarin	C12H12O5	237.08	320.80	POS
Arachidonic acid (AA)	C20H32O2	303.23	547.40	NEG
Betaine	C5H11NO2	118.09	48.40	POS
Biliverdin	C33H34N4O6	581.24	428.50	NEG
Butanoic acid	C4H8O2	87.05	235.80	NEG
Caprylic acid	C8H16O2	143.11	431.20	NEG
DL-Glutamic acid	C5H9NO4	146.05	41.50	NEG
Docosahexaenoic acid (DHA)	C22H32O2	327.23	540.80	NEG
Erucic acid	C22H42O2	337.31	610.30	NEG
gamma-Glutamylleucine	C11H20N2O5	261.14	215.40	POS
Glycine	C2H5NO2	74.02	48.90	NEG
Hydrocortisone	C21H30O5	363.22	344.30	POS
Hygric acid	C6H11NO2	130.09	48.60	POS
Methionine	C5H11NO2S	148.04	55.00	NEG
Methyl pentadecanoate	C16H32O2	255.23	569.30	NEG
Palmitic acid	C16H32O2	255.23	569.30	NEG
Paullinic acid	C20H38O2	309.28	590.30	NEG
Stearic acid	C18H36O2	283.26	590.90	NEG
Suberic acid	C8H14O4	173.08	296.60	NEG
Vidarabine	C10H13N5O4	268.10	195.30	POS
(1S,4aR,10aR)-1,4a,7-trimethyl-7-vinyl-3,4,4b,5,6,9,10,10a-octahydro-2H-phenanthrene-1-carboxylic acid	C20H30O2	301.22	546.80	NEG
(R)-5-Oxopyrrolidine-2-carboxylic acid	C5H7NO3	130.05	62.80	POS
12-Methyltridecanoic acid	C14H28O2	227.20	544.80	NEG
3-Hydorxy-3-methylglutaric acid	C6H10O5	161.05	94.80	NEG
3-Phosphoglyceric acid	C3H7O7P	184.99	75.10	NEG
4-[(3R)-3-hydroxybutyl]phenol	C10H14O2	165.09	288.50	NEG

4-Hydroxyhippuric acid	C9H9NO4	196.06	214.10	POS
4-Hydroxyphenylacetaldehyde	C8H8O2	135.05	231.50	NEG
9-Oxo-10(E),12(E)-octadecadienoic acid	C18H30O3	293.21	486.10	NEG
Asparagine	C4H8N2O3	133.06	49.50	POS
Citraconic acid	C5H6O4	129.02	124.30	NEG
Creatinine	C4H7N3O	114.07	45.50	POS
D(+)-Pipecolinic acid	C6H11NO2	130.09	48.60	POS
DL-Tryptophan	C11H12N2O2	203.08	207.60	NEG
Formylanthranilic acid	C8H7NO3	164.04	282.40	NEG
Guanine	C5H5N5O	152.06	76.50	POS
Hexadecanedioic acid	C16H30O4	285.21	474.00	NEG
Homoarginine	C7H16N4O2	189.13	45.30	POS
Hypoxanthine	C5H4N4O	137.05	81.00	POS
L-Methionine	C5H11NO2S	150.06	51.80	POS
methyl (E)-3-[4-[(2S,3R,4S,5S,6R)-3,4,5-trihydroxy-6-(hydroxymethyl)tetrahydropyran-2-yl]oxyphenyl]prop-2-enoate	C16H20O8	339.11	264.90	NEG
N1-Methyl-2-pyridone-5-carboxamide	C7H8N2O2	153.07	99.60	POS
Narcissin	C28H32O16	625.18	305.40	POS
Nervonic acid	C24H46O2	365.34	631.80	NEG
Nicotinamide	C6H6N2O	123.06	100.00	POS
Phenylephrine	C9H13NO2	150.09	52.60	POS
Salicylic acid	C7H6O3	137.02	253.90	NEG
Sorbose	C6H12O6	203.05	44.00	POS
Trehalose	C12H22O11	341.11	47.10	NEG
(2R,3S,4S,5R,6R)-2-(hydroxymethyl)-6-[(2S,3R,4S,5S,6R)-3,4,5-trihydroxy-6-(hydroxymethyl)tetrahydropyran-2-yl]oxy-tetrahydropyran-3,4,5-triol	C12H22O11	341.11	47.10	NEG
(2R,3S,4S,5R,6S)-2-(hydroxymethyl)-6-(2-hydroxyphenoxy)tetrahydropyran-3,4,5-triol	C12H16O7	271.08	219.20	NEG
2-Hydroxyhexanoic acid	C6H12O3	131.07	266.50	NEG
2-phenylethyl 3-(4-hydroxy-3-methoxy-phenyl)prop-2-enoate	C18H18O4	297.11	426.80	NEG
3-Hydroxylauric acid	C12H24O3	215.17	462.30	NEG
4-(4-methoxyphenyl)butan-2-one	C11H14O2	177.09	431.60	NEG
4-Hydroxycoumarin	C9H6O3	161.02	301.00	NEG
Azelaic acid	C9H16O4	187.10	329.50	NEG
Chiro-Inositol	C6H12O6	179.06	43.20	NEG
Cortisone	C21H28O5	361.20	339.60	POS

Costunolide	C15H20O2	233.15	413.00	POS
D-(+)-Cellobiose	C12H22O11	325.11	50.40	POS
D-Methionine	C5H11NO2S	150.06	51.80	POS
Dopamine	C8H11NO2	136.08	52.90	POS
Emodin	C15H10O5	269.05	457.50	NEG
ethyl 2,2-dimethyl-3-(2-methylprop-1-enyl)cyclopropanecarboxylate	C12H20O2	195.14	428.10	NEG
Fumaric acid	C4H4O4	115.00	83.70	NEG
Glucose	C6H12O6	203.05	44.00	POS
Itaconic acid	C5H6O4	129.02	124.30	NEG
L-Pipecolic acid	C6H11NO2	130.09	48.60	POS
Methionine sulfoxide	C5H11NO3S	166.05	53.40	POS
Methoxsalen	C12H8O4	217.05	350.60	POS
methyl 2-(2,4-dihydroxyphenyl)acetate	C9H10O4	181.05	256.20	NEG
Methylpyroglutamate	C6H9NO3	144.07	117.60	POS
N,N-Dimethylarginine (ADMA)	C8H18N4O2	203.15	45.70	POS
N6,N6,N6-Trimethyllysine	C9H20N2O2	189.16	42.20	POS
Octadecanedioic acid	C18H34O4	313.24	504.30	NEG
Octopamine	C8H11NO2	136.08	52.90	POS
Phenylalanine	C9H11NO2	166.09	108.10	POS
Quercetin	C15H10O7	301.04	350.10	NEG
Sucrose	C12H22O11	365.11	44.30	POS
Traumatic acid	C12H20O4	227.13	388.90	NEG
Tyrosine	C9H11NO3	182.08	75.90	POS
Valproic acid	C8H16O2	143.11	431.20	NEG
(E)-3-(2,5-dihydroxyphenyl)prop-2-enoic acid	C9H8O4	179.03	236.10	NEG
1-Methylhistidine	C7H11N3O2	170.09	45.40	POS
14,16-dihydroxy-4-methyl-3-oxabicyclo[10.4.0]hexadeca-1(12),13,15-trien-2-one	C16H22O4	277.14	468.70	NEG
2-Piperidone	C5H9NO	100.08	140.00	POS
2,6-Dihydroxybenzoic acid	C7H6O4	153.02	297.60	NEG
4-O-p-Coumaroylquinic acid	C16H18O8	339.11	259.50	POS
7-(1-hydroxy-1-methyl-ethyl)-1,4a-dimethyl-2,3,4,9,10,10a-hexahydrophenanthrene-1-carboxylic acid	C20H28O3	315.20	439.90	NEG
Adenosine	C10H13N5O4	268.10	195.30	POS
Arabinose	C5H10O5	149.05	44.80	NEG
Caffeic acid	C9H8O4	179.03	268.60	NEG

Chlorpheniramine (maleate)	C16H19ClN2	275.13	295.70	POS
Choline	C5H14NO	104.11	43.90	POS
Diglycine	C4H8N2O3	131.05	48.10	NEG
Fagomine	C6H13NO3	148.10	49.20	POS
Galactitol	C6H14O6	181.07	44.10	NEG
gamma-Glutamylvaline	C10H18N2O5	245.11	109.30	NEG
gamma-L-Glutamyl-L-phenylalanine	C14H18N2O5	295.13	226.30	POS
Glucitol	C6H14O6	181.07	44.10	NEG
Glutamate	C5H9NO4	146.05	41.50	NEG
Glutaric acid	C5H8O4	131.03	120.30	NEG
Glyceric acid	C3H6O4	105.02	47.80	NEG
Homoveratric acid	C10H12O4	195.07	283.70	NEG
Indole-3-carboxaldehyde	C9H7NO	144.05	295.20	NEG
Isoleucine	C6H13NO2	130.09	71.10	NEG
Isopimaric acid	C20H30O2	301.22	531.00	NEG
Ligustilide	C12H14O2	173.10	434.00	POS
Melibiose	C12H22O11	365.11	44.30	POS
Myristoleic acid	C14H26O2	225.19	524.10	NEG
Orcinol	C7H8O2	123.05	277.80	NEG
Palatinose (hydrate)	C12H22O11	341.11	47.10	NEG
Phe-Leu	C15H22N2O3	277.16	257.60	NEG
(4S,5Z,6S)-5-[2-[(E)-3-(4-hydroxyphenyl)prop-2-enoyl]oxyethylidene]-4-(2-methoxy-2-oxo-ethyl)-6-[(2S,3R,4S,5S,6R)-3,4,5-trihydroxy-6-(hydroxymethyl)tetrahydropyran-2-yl]oxy-4H-pyran-3-carboxylic acid	C26H30O14	549.16	285.60	POS
(E)-3-(4-hydroxy-3-methoxy-phenyl)prop-2-enamide	C10H11NO3	176.07	236.10	POS
(E)-8-hydroxy-2,6-dimethyl-oct-2-enoic acid	C10H18O3	185.12	346.20	NEG
(E)-Methyl 4-coumarate	C10H10O3	179.07	328.10	POS
2-(3,4-dihydroxyphenyl)-5-hydroxy-7-methoxy-3-[(2S,3R,4S,5S,6R)-3,4,5-trihydroxy-6-[[(2R,3R,4R,5R,6S)-3,4,5-trihydroxy-6-methyl-tetrahydropyran-2-yl]oxymethyl]tetrahydropyran-2-yl]oxy-chromen-4-one	C28H32O16	625.18	305.40	POS
2-[(1R,2R)-3-oxo-2-[(Z)-5-[3,4,5-trihydroxy-6-(hydroxymethyl)tetrahydropyran-2-yl]oxypent-2-enyl]cyclopentyl]acetic acid	C18H28O9	387.17	248.00	NEG
3-Hydroxyoctanoic acid	C8H16O3	159.10	355.20	NEG
3,4-Dihydroxyphenylacetic acid	C8H8O4	167.03	222.80	NEG
4-Allylcatechol	C9H10O2	149.06	357.60	NEG
5-methyl-4-[(2S,3R,4S,5S,6R)-3,4,5-trihydroxy-6-(hydroxymethyl)tetrahydropyran-2-yl]oxy-chromen-2-one	C16H18O8	339.11	259.50	POS
7,8-dimethoxychromen-2-one	C11H10O4	207.07	304.10	POS

Arachidic acid	C20H40O2	311.30	611.70	NEG
Butylphthalide	C12H14O2	173.10	401.50	POS
cis-4-Hydroxy-D-proline	C5H9NO3	130.05	55.50	NEG
D-Kynurenine	C10H12N2O3	209.09	105.50	POS
Ethyl 4-hydroxybenzoate	C9H10O3	165.06	342.90	NEG
Hydroxyisocaproic acid	C6H12O3	131.07	266.50	NEG
Iditol	C6H14O6	181.07	44.10	NEG
Isoquinoline	C9H7N	130.06	209.60	POS
Ketoleucine	C6H10O3	129.06	248.10	NEG
L-Allothreonine	C4H9NO3	120.07	46.00	POS
Maltose	C12H22O11	365.11	44.30	POS
N-Acetylmethionine	C7H13NO3S	190.05	207.70	NEG
Oleuropein	C25H32O13	539.18	310.40	NEG
Phthalic acid	C8H6O4	165.02	240.90	NEG
Tropolone	C7H6O2	121.03	300.50	NEG
Tryptophan	C11H12N2O2	205.10	209.20	POS
Vicenin 2	C27H30O15	593.15	262.80	NEG
(2S,3R,4S,5S,6R)-2-[4-[(3R,3aR,6R,6aR)-6-[3,5-dimethoxy-4-[(2S,3R,4S,5S,6R)-3,4,5-trihydroxy-6-(hydroxymethyl)tetrahydropyran-2-yl]oxy-phenyl]-1,3,3a,4,6,6a-hexahydrofuro[3,4-c]furan-3-yl]-2,6-dimethoxy-phenoxy]-6-(hydroxymethyl)tetrahydropyran-3,4,5-triol	C34H46O18	741.26	252.20	NEG
(2S,3R,4S,5S,6R)-2-[4-[(3S,3aR,6S,6aR)-6-[3,5-dimethoxy-4-[(2S,3R,4S,5S,6R)-3,4,5-trihydroxy-6-(hydroxymethyl)tetrahydropyran-2-yl]oxy-phenyl]-1,3,3a,4,6,6a-hexahydrofuro[3,4-c]furan-3-yl]-2,6-dimethoxy-phenoxy]-6-(hydroxymethyl)tetrahydropyran-3,4,5-triol	C34H46O18	741.26	252.20	NEG
1-Methylpyrrolidine	C5H11N	86.10	55.00	POS
12-Hydroxystearic acid	C18H36O3	299.26	510.40	NEG
2,3-Dihydroxy-4-methoxyacetophenone	C9H10O4	181.05	256.20	NEG
3-Phenylpropanoic acid	C9H10O2	149.06	357.60	NEG
4-Hydroxybenzaldehyde	C7H6O2	121.03	273.60	NEG
Allantoin	C4H6N4O3	157.04	41.10	NEG
Benzoic acid	C7H6O2	121.03	325.30	NEG
D-(+)-Melibiose	C12H22O11	341.11	47.10	NEG
Isomaltose	C12H22O11	325.11	50.40	POS
L-Uridine	C9H12N2O6	243.06	86.90	NEG
Maleic acid	C4H4O4	115.00	104.50	NEG

N-Acetylphenylalanine	C11H13NO3	206.08	276.70	NEG
N5-acetyl-l-ornithine	C7H14N2O3	175.11	50.80	POS
Sphingosine	C18H37NO2	300.29	434.40	POS
Valeric acid	C5H10O2	101.06	316.80	NEG
1-(1H-indol-3-yl)ethanone	C10H9NO	158.06	295.20	NEG
2-(4-hydroxyphenyl)ethyl (E)-3-(4-hydroxy-3-methoxy-phenyl)prop-2-enoate	C18H18O5	313.11	380.00	NEG
2-oxindole-3-acetate	C10H9NO3	190.05	271.20	NEG
3-Indoleacrylic acid	C11H9NO2	186.06	346.70	NEG
4-Hydroxyproline	C5H9NO3	130.05	55.50	NEG
4'-Hydroxyacetophenone	C8H8O2	135.04	191.10	NEG
5'-Methylthioadenosine	C11H15N5O3S	298.10	246.30	POS
Caproic acid	C6H12O2	115.08	368.70	NEG
cis-4-Hydroxy-L-proline	C5H9NO3	130.05	55.50	NEG
Gluconic acid	C6H12O7	195.05	42.60	NEG
Hesperetin	C16H14O6	301.07	359.30	NEG
Indole-3-methyl acetate	C11H11NO2	188.07	350.00	NEG
Isocitric acid	C6H8O7	191.02	64.10	NEG
Kynurenic Acid	C10H7NO3	190.05	241.90	POS
Phenyl glucuronide	C12H14O7	269.07	209.80	NEG
Phloroglucinol	C6H6O3	127.04	101.80	POS
Phthalic acid mono-2-ethylhexyl ester	C16H22O4	277.14	468.70	NEG
Rutin	C27H30O16	609.15	293.40	NEG
Thymidine	C10H14N2O5	241.08	160.50	NEG
(10E,12E)-9-hydroxyoctadeca-10,12-dienoic acid	C18H32O3	295.23	486.60	NEG
(E)-9,12,13-trihydroxyoctadec-10-enoic acid	C18H34O5	329.23	395.40	NEG
(Rac)-Hesperetin	C16H14O6	301.07	359.30	NEG
(Z)-6,9,10-trihydroxyoctadec-7-enoic acid	C18H34O5	329.23	409.80	NEG
[3-acetoxy-2-[3,4,5-trihydroxy-6-(hydroxymethyl)tetrahydropyran-2-yl]oxy-propyl] 3-(4-hydroxyphenyl)prop-2-enoate	C20H26O11	441.14	291.60	NEG
1-[3-hydroxy-4-[(2S,3R,4S,5S,6R)-3,4,5-trihydroxy-6-(hydroxymethyl)tetrahydropyran-2-yl]oxy-phenyl]ethanone	C14H18O8	313.09	206.30	NEG
1-methyl-2,3,4,9-tetrahydro-1H-pyrido[3,4-b]indole-3-carboxylic acid	C13H14N2O2	231.11	248.60	POS
2-Hydroxy-6-methoxybenzoic acid	C8H8O4	167.03	291.10	NEG
2-Hydroxycinnamic acid	C9H8O3	163.04	215.20	NEG
7-hydroxy-2-(4-methoxyphenyl)chromen-4-one	C16H12O4	267.07	382.50	NEG
Cellobiose	C12H22O11	325.11	50.40	POS

Desaminotyrosine	C9H10O3	165.06	277.60	NEG
Indole	C8H7N	116.05	349.90	NEG
Isorhamnetin-3-O-neohespeidoside	C28H32O16	625.18	305.40	POS
Pyroglutamic acid	C5H7NO3	130.05	62.80	POS
Salicyluric acid	C9H9NO4	194.05	273.10	NEG
Scopolin	C16H18O9	353.09	233.40	NEG
Succinyladenosine	C14H17N5O8	382.10	206.80	NEG
Theanine	C7H14N2O3	173.09	47.60	NEG
Umbelliferone	C9H6O3	161.02	301.00	NEG
Xylose	C5H10O5	131.03	44.10	NEG
(2R,3R)-3-(4-hydroxy-3-methoxy-phenyl)-2-(hydroxymethyl)-5-methoxy-2,3-dihydropyrano[3,2-h][1,4]benzodioxin-9-one	C20H18O8	387.11	322.10	POS
2-Isopropylmalic acid	C7H12O5	175.06	218.80	NEG
2-Methylcaproic acid	C7H14O2	129.09	403.70	NEG
3-[1-hydroxy-2-(methylamino)ethyl]phenol	C9H13NO2	150.09	52.60	POS
4-Hydroxybenzoic acid	C7H6O3	137.02	253.90	NEG
4-Hydroxycinnamic acid	C9H8O3	163.04	398.90	NEG
5,7-dihydroxy-2-(4-hydroxy-3-methoxy-phenyl)-3-[3,4,5-trihydroxy-6-[[(2R,3R,4R,5R,6S)-3,4,5-trihydroxy-6-methyl-tetrahydropyran-2-yl]oxymethyl]tetrahydropyran-2-yl]oxy-chromen-4-one	C28H32O16	625.18	305.40	POS
5,7-Dihydroxychromone	C9H6O4	177.02	296.30	NEG
Adenosine monophosphate (AMP)	C10H14N5O7P	346.05	78.20	NEG
Fucose	C6H12O5	187.06	46.80	POS
Isatin	C8H5NO2	146.02	263.40	NEG
Malonic acid	C3H4O4	103.00	62.30	NEG
Mandelic acid	C8H8O3	151.04	219.30	NEG
Paeonol	C9H10O3	165.06	163.70	NEG
Pinobanksin	C15H12O5	271.06	352.60	NEG
Riboflavin	C17H20N4O6	377.15	253.80	POS
Uridine	C9H12N2O6	243.06	86.90	NEG
(1S,3R,4R,5R)-1,3,4-trihydroxy-5-[(E)-3-(4-hydroxy-3-methoxy-phenyl)prop-2-enoyl]oxy-cyclohexanecarboxylic acid	C17H20O9	367.10	260.10	NEG
(2R,3S,4S,5R,6R)-2-[[(2R,3R,4R)-3,4-dihydroxy-4-(hydroxymethyl)tetrahydrofuran-2-yl]oxymethyl]-6-[2-(4-hydroxyphenyl)ethoxy]tetrahydropyran-3,4,5-triol	C19H28O11	431.16	231.20	NEG
(2R)-2-phenyl-2-[(2S,3R,4S,5S,6R)-3,4,5-trihydroxy-6-(hydroxymethyl)tetrahydropyran-2-yl]oxy-acetic acid	C14H18O8	313.09	176.50	NEG
(E,6R)-2,6-dimethyl-8-[(2R,3R,4S,5S,6R)-3,4,5-trihydroxy-6-(hydroxymethyl)tetrahydropyran-2-yl]oxy-oct-2-enoic acid	C16H28O8	347.17	302.90	NEG
17-ODYA	C18H32O2	279.23	553.30	NEG

3-Methylcatechol	C7H8O2	123.05	277.80	NEG
3,4-Dicaffeoylquinic acid	C25H24O12	515.12	301.60	NEG
4-Vinylphenol	C8H8O	121.06	378.00	POS
5-Aminolevulinic acid	C5H9NO3	130.05	55.50	NEG
Lactate	C3H6O3	89.02	52.10	NEG
Uracil	C4H4N2O2	113.03	51.90	POS
(2E,4E)-5-[8-hydroxy-1,5-dimethyl-3-[3,4,5-trihydroxy-6-(hydroxymethyl)tetrahydropyran-2-yl]oxy-6-oxabicyclo[3.2.1]octan-8-yl]-3-methyl-penta-2,4-dienoic acid	C21H32O10	443.19	231.20	NEG
(E,9S,12S,13S)-9,12,13-trihydroxyoctadec-10-enoic acid	C18H34O5	329.23	395.40	NEG
6-(hydroxymethyl)pyridin-3-ol	C6H7NO2	124.04	74.80	NEG
Citric acid	C6H8O7	191.02	64.10	NEG
Cyclo(L-Phe-L-Pro)	C14H16N2O2	245.13	292.90	POS
Malic acid	C4H6O5	133.01	51.10	NEG
Pyrogallol	C6H6O3	127.04	133.70	POS
Ribitol	C5H12O5	151.06	40.30	NEG
Terephthalic-Acid	C8H6O4	165.02	240.90	NEG
(3Z)-3-butylidene-5-hydroxy-isobenzofuran-1-one	C12H12O3	203.07	414.70	NEG
2-Aminopimelate	C7H13NO4	174.08	69.30	NEG
2,2'-Iminodiacetic acid	C4H7NO4	132.03	41.10	NEG
3,5,7-trihydroxy-8-methoxy-2-(4-methoxyphenyl)chromen-4-one	C17H14O7	329.07	383.70	NEG
4-Acetamidobutyric acid	C6H11NO3	144.07	99.50	NEG
7-Hydroxy-4H-chromen-4-one	C9H6O3	161.02	301.00	NEG
alpha-Ketoglutaric acid (alpha-KG)	C5H6O5	145.01	64.00	NEG
Diacetyl	C4H6O2	85.03	121.80	NEG
Hydroxytyrosol acetate	C10H12O4	195.07	283.70	NEG
Isocaproic acid	C6H12O2	115.08	368.70	NEG
Neantine	C12H14O4	205.09	399.70	POS
Verbenalin	C17H24O10	369.12	245.10	NEG
1-beta-D-Arabinofuranosyluracil	C9H12N2O6	243.06	86.90	NEG
3-Hydroxydodecanoic acid	C12H24O3	215.17	462.30	NEG
3-Methoxytyramine	C9H13NO2	150.09	52.60	POS
3',5'-Dimethoxy-4'-hydroxyacetophenone	C10H12O4	195.07	265.30	NEG
6-Hydroxycoumarin	C9H6O3	161.02	301.00	NEG
7-methoxy-8-(3-methyl-5-oxo-2H-furan-4-yl)chromen-2-one	C15H12O5	273.08	286.10	POS

Coniferol	C10H12O3	179.07	278.40	NEG
Crotonoside	C10H13N5O5	284.10	75.90	POS
Homoeriodictyol	C16H14O6	301.07	359.30	NEG
Isoformononetin	C16H12O4	267.07	382.50	NEG
N-Methylglutamic acid	C6H11NO4	142.05	62.20	NEG
Scoparone	C11H10O4	207.07	304.10	POS
(10E,15Z)-9,12,13-trihydroxyoctadeca-10,15-dienoic acid	C18H32O5	327.22	385.10	NEG
2-Aminoadipic acid	C6H11NO4	160.06	66.60	NEG
2-Hydroxypalmitic acid	C16H32O3	271.23	498.20	NEG
2-Hydroxystearic acid	C18H36O3	299.26	567.70	NEG
2-Ketobutyric acid	C4H6O3	101.02	102.80	NEG
3-[(2S,3R,4S,5R,6R)-3,5-dihydroxy-6-(hydroxymethyl)-4-[(2S,3R,4R,5R,6S)-3,4,5-trihydroxy-6-methyl-tetrahydropyran-2-yl]oxy-tetrahydropyran-2-yl]oxy-5,7-dihydroxy-2-(4-hydroxyphenyl)chromen-4-one	C27H30O15	593.15	305.90	NEG
3,5-Di-tert-butylphenol	C14H22O	205.16	500.10	NEG
6-Hydroxyluteolin 7-glucoside	C21H20O12	465.10	300.80	POS
Indirubin	C16H10N2O2	263.08	406.30	POS
Licoricone	C22H22O6	381.13	416.50	NEG
Monomethyl fumarate	C5H6O4	129.02	146.00	NEG
NAE(18:2)	C20H37NO2	324.29	523.80	POS
Propyl paraben	C10H12O3	179.07	400.30	NEG
(1S,4aS,7R,7aR)-7-methyl-1-[(2S,3R,4S,5S,6R)-3,4,5-trihydroxy-6-(hydroxymethyl)tetrahydropyran-2-yl]oxy-1,4a,5,6,7,7a-hexahydrocyclopenta[c]pyran-4-carboxylic acid	C16H24O9	359.13	264.60	NEG
(2R,3R,4S,5S,6R)-2-[2-(4-methoxyphenyl)ethoxy]-6-[[(2S,3R,4S,5S)-3,4,5-trihydroxytetrahydropyran-2-yl]oxymethyl]tetrahydropyran-3,4,5-triol	C20H30O11	445.17	243.90	NEG
2-hydroxy-3-methoxy-benzaldehyde	C8H8O3	151.04	274.90	NEG
2-Phenyllactic acid	C9H10O3	165.06	277.60	NEG
2,3-bis[(4-hydroxy-3-methoxy-phenyl)methyl]butane-1,4-diol	C20H26O6	361.17	310.40	NEG
3-benzyl-2,3,6,7,8,8a-hexahydropyrrolo[1,2-a]pyrazine-1,4-dione	C14H16N2O2	245.13	284.40	POS
Isovaleric acid	C5H10O2	101.06	316.80	NEG
N-Acetylglutamic acid	C7H11NO5	188.06	70.60	NEG
(1S,3R,4R,5R)-1,3-bis[[(E)-3-(3,4-dihydroxyphenyl)prop-2-enoyl]oxy]-4,5-dihydroxy-cyclohexanecarboxylic acid	C25H24O12	499.12	295.50	POS
(2R,3S,4S,5R,6R)-2-[[(2S,3R,4R)-3,4-dihydroxy-4-(hydroxymethyl)tetrahydrofuran-2-yl]oxymethyl]-6-(2-phenylethoxy)tetrahydropyran-3,4,5-triol	C19H28O10	415.16	280.20	NEG
2-[4-[4-[hydroxy-(4-hydroxy-3-methoxy-phenyl)methyl]-3-(hydroxymethyl)tetrahydrofuran-2-yl]-2-methoxy-phenoxy]-6-(hydroxymethyl)tetrahydropyran-3,4,5-triol	C26H34O12	537.20	255.80	NEG

2-Ketogluconic acid	C6H10O7	193.04	42.30	NEG
2-methylcitrate	C7H10O7	205.04	64.50	NEG
3-Hydroxybutyric acid	C4H8O3	87.04	76.60	POS
3,4-Dimethylbenzoic acid	C9H10O2	149.06	357.60	NEG
4-Feruloylquinic acid	C17H20O9	349.09	273.90	NEG
Monobutyl phthalate	C12H14O4	205.09	399.70	POS
phenethanolamine	C8H11NO	120.08	108.10	POS
[(1S,4aS,5R,7S,7aS)-4a,5-dihydroxy-7-methyl-1-[(2S,3R,4S,5S,6R)-3,4,5-trihydroxy-6-(hydroxymethyl)tetrahydropyran-2-yl]oxy-1,5,6,7a-tetrahydrocyclopenta[c]pyran-7-yl] acetate	C17H26O11	387.13	251.70	NEG
2-[(4-hydroxy-3-methoxy-phenyl)-[5-(4-hydroxy-3-methoxy-phenyl)-4-(hydroxymethyl)tetrahydrofuran-3-yl]methoxy]-6-(hydroxymethyl)tetrahydropyran-3,4,5-triol	C26H34O12	537.20	255.80	NEG
2-indolecarboxylic-acid	C9H7NO2	160.04	312.20	NEG
2'-Hydroxy-5'-methylacetophenone	C9H10O2	149.06	357.60	NEG
3-Hydroxyacetophenone	C8H8O2	135.05	32.30	NEG
3-methylolphenol	C7H8O2	125.06	237.40	POS
5,7-dihydroxy-2-(4-hydroxyphenyl)-6,8-bis[3,4,5-trihydroxy-6-(hydroxymethyl)tetrahydropyran-2-yl]chromen-4-one	C27H30O15	593.15	262.80	NEG
7,10-bis(1,1-dimethylallyl)-5-hydroxy-2,2-dimethyl-pyrano[3,2-g]chromen-8-one	C24H28O4	381.21	528.90	POS
D-2-Amino-4-methylpentanoic acid	C6H13NO2	130.09	656.60	NEG
TBHQ	C10H14O2	165.09	361.30	NEG
(E)-Osmundacetone	C10H10O3	179.07	304.50	POS
1-Deoxyxylulose 5-phosphate	C5H11O7P	213.02	46.50	NEG
1,9b-dihydroxy-6,6,9a-trimethyl-1,5,5a,7,8,9-hexahydrobenzo[e]isobenzofuran-3-one	C15H22O4	265.14	417.10	NEG
3,7-Dimethyluric acid	C7H8N4O3	195.05	128.80	NEG
Cytosine	C4H5N3O	112.05	66.20	POS
(1R,7R,10R)-4,10,11,11-tetramethyltricyclo[5.3.1.01,5]undec-4-en-3-one	C15H22O	219.17	431.80	POS
(1S,4aS,7S,7aR)-7-methyl-1-[(2S,3R,4S,5S,6R)-3,4,5-trihydroxy-6-(hydroxymethyl)tetrahydropyran-2-yl]oxy-1,4a,5,6,7,7a-hexahydrocyclopenta[c]pyran-4-carboxylic acid	C16H24O9	359.13	264.60	NEG
(2R,3S,4S,5R,6R)-2-(hydroxymethyl)-6-[[(2R,3S,4S,5R,6S)-3,4,5-trihydroxy-6-(3-hydroxy-5-methyl-phenoxy)tetrahydropyran-2-yl]methoxy]tetrahydropyran-3,4,5-triol	C19H28O12	447.15	198.20	NEG
14-hydroxy-16-methoxy-4-methyl-3-oxabicyclo[10.4.0]hexadeca-1(12),13,15-trien-2-one	C17H24O4	291.16	464.30	NEG
2,5-Dihydroxyacetophenone	C8H8O3	151.04	274.90	NEG
3-Methoxyphenylacetic acid	C9H10O3	165.06	342.90	NEG
9-hydroxy-7-isopropyl-1,4a-dimethyl-2,3,4,9,10,10a-hexahydrophenanthrene-1-carboxylic acid	C20H28O3	315.20	439.90	NEG
Aldosterone	C21H28O5	361.20	339.60	POS
Cassiaside C	C27H32O15	595.17	284.30	NEG

Complanatuside	C28H32O16	625.18	278.60	POS
Deoxyadenosine	C10H13N5O3	252.11	203.40	POS
Pyruvate	C3H4O3	87.01	51.80	NEG
(1R,2R,4aR,8aS)-1-[2-(3-furyl)ethyl]-2,4a,5-trimethyl-2,3,4,7,8,8a-hexahydronaphthalene-1-carboxylic acid	C20H28O3	315.20	439.90	NEG
(4S,5Z,6S)-4-(2-methoxy-2-oxo-ethyl)-5-[2-[(E)-3-phenylprop-2-enoyl]oxyethylidene]-6-[(2S,3R,4S,5S,6R)-3,4,5-trihydroxy-6-(hydroxymethyl)tetrahydropyran-2-yl]oxy-4H-pyran-3-carboxylic acid	C26H30O13	533.16	310.70	POS
12-Hydroxydodecanoic acid	C12H24O3	215.17	407.90	NEG
2-Hydroxyadenine	C5H5N5O	152.06	76.50	POS
3,5-dihydroxy-2-(3-hydroxy-4-methoxy-phenyl)-7-methoxy-chromen-4-one	C17H14O7	329.07	383.70	NEG
5-Hydroxyindole	C8H7NO	134.06	271.20	POS
Isochlorogenic acid A	C25H24O12	517.13	300.20	POS
Rubrofusarin gentiobioside	C27H32O15	595.17	284.30	NEG
(2S)-3-(4-hydroxyphenyl)-2-[[(E)-3-(4-hydroxyphenyl)prop-2-enoyl]amino]propanoic acid	C18H17NO5	328.12	296.80	POS
3-Hydroxyisovaleric acid	C5H10O3	117.06	87.00	NEG
4-methoxy-6-methyl-7,8-dihydro-5H-[1,3]dioxolo[4,5-g]isoquinoline	C12H15NO3	222.11	234.30	POS
4'-Hydroxy-3'-methylacetophenone	C9H10O2	149.06	357.60	NEG
4′-Hydroxy-2′-methylacetophenone	C9H10O2	149.06	357.60	NEG
D-Mannoheptulose	C7H14O7	209.07	47.60	NEG
Guanidinosuccinic acid	C5H9N3O4	176.07	55.50	POS
HTMF	C19H18O7	357.10	340.40	NEG
Praeruptorin C	C24H28O7	411.18	446.00	POS
(8R)-1,5,8-trimethyl-7,8-dihydro-6H-azuleno[6,5-b]furan-2-one	C15H16O2	229.12	481.60	POS
1,4-dihydroxy-6,6,9a-trimethyl-4,5,5a,7,8,9-hexahydro-1H-benzo[e]isobenzofuran-3-one	C15H22O4	265.14	417.10	NEG
2-(2-phenylethoxy)-6-[(3,4,5-trihydroxytetrahydropyran-2-yl)oxymethyl]tetrahydropyran-3,4,5-triol	C19H28O10	415.16	280.20	NEG
2-[(2S,4aR,8aS)-2-hydroxy-4a-methyl-8-methylene-decalin-2-yl]prop-2-enoic acid	C15H22O3	249.15	414.90	NEG
2-Furoic acid	C5H4O3	111.01	100.90	NEG
2-Hydroxyhexanedioic acid	C6H10O5	143.03	72.70	NEG
Ellagic acid	C14H6O8	301.00	467.70	NEG
Lonicerin	C27H30O15	593.15	305.90	NEG
methyl 2-hydroxy-3-[(2S,3R,4S,5S,6R)-3,4,5-trihydroxy-6-(hydroxymethyl)tetrahydropyran-2-yl]oxy-benzoate	C14H18O9	329.09	204.10	NEG
Methyl isovanillate	C9H10O4	181.05	256.20	NEG
Peperomin F	C24H26O10	473.15	316.50	NEG
Piperonylic acid	C8H6O4	165.02	332.90	NEG
Pyruvaldehyde	C3H4O2	71.01	48.20	NEG

Threonic acid	C4H8O5	135.03	100.30	NEG
(4aS)-6,7-dihydroxy-1,1,4a-trimethyl-3,4,10,10a-tetrahydro-2H-phenanthren-9-one	C17H22O3	273.15	472.00	NEG
1-[2,4-dihydroxy-6-[(2S,3R,4S,5S,6R)-3,4,5-trihydroxy-6-(hydroxymethyl)tetrahydropyran-2-yl]oxy-phenyl]ethanone	C14H18O9	329.09	204.10	NEG
1-[4-hydroxy-3-[(2S,3R,4S,5S,6R)-3,4,5-trihydroxy-6-(hydroxymethyl)tetrahydropyran-2-yl]oxy-phenyl]ethanone	C14H18O8	313.09	176.50	NEG
10-methoxy-2,2-dimethyl-pyrano[3,2-g]chromen-8-one	C15H14O4	259.10	328.90	POS
2-Acetonaphthone	C12H10O	171.08	424.00	POS
3-(3,4,5-Trimethoxyphenyl)propanoic acid	C12H16O5	241.11	350.60	POS
3,5,7-trihydroxy-2-[2-hydroxy-6-[(2S,3R,4S,5S,6R)-3,4,5-trihydroxy-6-(hydroxymethyl)tetrahydropyran-2-yl]oxy-phenyl]chromen-4-one	C21H20O12	465.10	300.80	POS
5-Hydroxyindole-3-acetic acid	C10H9NO3	190.05	271.20	NEG
Azaleatin	C16H12O7	317.07	368.60	POS
Butin	C15H12O5	271.06	352.60	NEG
Citropten	C11H10O4	207.07	304.10	POS
D-(+)-Mannose	C6H12O6	179.06	284.70	NEG
DL-Glutamine	C5H10N2O3	147.08	63.10	POS
Ethyl Vanillate	C10H12O4	195.07	283.70	NEG
Mannitol	C6H14O6	183.09	37.60	POS
Phenol	C6H6O	93.03	248.50	NEG
Tyramine	C8H11NO	120.08	108.10	POS
(2R,3R,4S,5S,6R)-2-[(2E,6R)-6-hydroxy-2,6-dimethyl-octa-2,7-dienoxy]-6-(hydroxymethyl)tetrahydropyran-3,4,5-triol	C16H28O7	331.18	296.90	NEG
1-Caffeoylquinic acid	C16H18O9	353.09	233.40	NEG
2-Hydroxy-4-methylbenzaldehyde	C8H8O2	137.06	408.20	POS
2-Methylphenol	C7H8O	107.05	303.60	NEG
2,6-Dimethylphenol	C8H10O	121.07	348.60	NEG
3-Cresol	C7H8O	107.05	303.60	NEG
4-[1-hydroxy-2-(methylamino)ethyl]benzene-1,2-diol	C9H13NO3	164.07	103.90	NEG
7-methoxy-9,10-dihydrophenanthrene-2,5-diol	C15H14O3	243.10	304.50	POS
Torachrysone-8-O-b-D-glucoside	C20H24O9	389.12	382.70	NEG
(2S,3R,4S,5S,6R)-2-[4-(2,3-dihydroxypropyl)-3-methoxy-phenoxy]-6-(hydroxymethyl)tetrahydropyran-3,4,5-triol	C16H24O9	359.13	264.60	NEG
(3aR,5S,5aS,8R,8aS,9aR)-8-hydroxy-5,8a-dimethyl-1-methylene-3a,4,5,5a,6,8,9,9a-octahydroazuleno[6,5-b]furan-2,7-dione	C15H20O4	263.13	322.90	NEG
[(7R,8R,10R,13S,17R)-17-(3-furyl)-4,4,8,10,13-pentamethyl-3,16-dioxo-6,7,9,11,12,17-hexahydro-5H-cyclopenta[a]phenanthren-7-yl] acetate	C28H34O5	431.22	431.20	NEG
1,3,6-trihydroxy-8-(3-hydroxy-3-methyl-butyl)-7-methoxy-2-(3-methylbut-2-enyl)xanthen-9-one	C24H28O7	427.18	450.00	NEG
2,3-Dimethylphenol	C8H10O	121.07	348.60	NEG

5-Hydroxyhexanoic acid	C6H12O3	131.07	266.50	NEG
D-Phenylalanine	C9H11NO2	166.09	108.10	POS
Enterolactone	C18H18O4	299.13	315.40	POS
Leucylphenylalanine	C15H22N2O3	277.16	257.60	NEG
sn-Glycerol 1-phosphate	C3H9O6P	171.01	52.50	NEG
sn-Glycerol 3-phosphate	C3H9O6P	171.01	52.50	NEG
Tropone	C7H6O	107.05	186.20	POS
Vanillic acid	C8H8O4	149.02	274.40	NEG
2,5-Dimethylphenol	C8H10O	121.07	348.60	NEG
4-Ethylphenol	C8H10O	121.07	348.60	NEG
Andrographolide	C20H30O5	349.20	377.30	NEG
Homogentisic acid	C8H8O4	167.03	222.80	NEG
methyl (1S,4aS,5S,7aS)-7-(acetoxymethyl)-5-hydroxy-1-[(2S,3R,4S,5S,6R)-3,4,5-trihydroxy-6-(hydroxymethyl)tetrahydropyran-2-yl]oxy-1,4a,5,7a-tetrahydrocyclopenta[c]pyran-4-carboxylate	C19H26O12	427.12	280.00	NEG
Pyridoxine	C8H11NO3	170.08	68.60	POS
(2Z,4E)-5-[8-hydroxy-1,5-dimethyl-3-[(2R,3R,4S,5S,6R)-3,4,5-trihydroxy-6-(hydroxymethyl)oxan-2-yl]oxy-6-oxabicyclo[3.2.1]octan-8-yl]-3-methylpenta-2,4-dienoic acid	C21H32O10	427.20	232.10	POS
(3E,4R)-3-[2-[(1R,4aS,5R,6R,8aS)-6-hydroxy-5-(hydroxymethyl)-5,8a-dimethyl-2-methylene-decalin-1-yl]ethylidene]-4-hydroxy-tetrahydrofuran-2-one	C20H30O5	349.20	377.30	NEG
2-Methylbenzoic acid	C8H8O2	135.05	318.60	NEG
3-Ethylphenol	C8H10O	121.07	348.60	NEG
Allitol	C6H14O6	181.07	44.10	NEG
Podocarpic acid	C17H22O3	273.15	461.30	NEG
Corydaldine (tautomeric structure 1)	C11H13NO3	208.10	295.60	POS
Syringin	C17H24O9	371.13	269.30	NEG
(2S,3R,4R,5R,6S)-2-[(2R,3R,4S,5R,6R)-2-[2-(3,4-dihydroxyphenyl)ethoxy]-3,5-dihydroxy-6-(hydroxymethyl)tetrahydropyran-4-yl]oxy-6-methyl-tetrahydropyran-3,4,5-triol	C20H30O12	443.16	259.90	NEG
3-Furoic acid	C5H4O3	111.01	195.60	NEG
Brefeldin A	C16H24O4	279.16	410.30	NEG
Magnolioside	C16H18O9	353.09	233.40	NEG
2-(hydroxymethyl)-6-[[(1S,4R)-1,3,3-trimethyl-2-oxabicyclo[2.2.2]octan-6-yl]oxy]tetrahydropyran-3,4,5-triol	C16H28O7	331.18	296.90	NEG
3,4-Dihydroxyphenylalanine (DOPA)	C9H11NO4	196.06	103.90	NEG
5-Oxooctanoic acid	C8H14O3	157.09	344.30	NEG
6,8-dihydroxy-9-isopropyl-2,2,4,4-tetramethyl-5-(2-methylpropanoyl)-9H-xanthene-1,3-dione	C24H30O6	413.20	441.40	NEG
Hamaudol	C15H16O5	259.10	328.90	POS

Matairesinol	C20H22O6	341.14	382.70	POS
Rosarin	C20H28O10	409.15	324.30	NEG
(2R,3R,4S,5S,6R)-2-[(E)-cinnamyl]oxy-6-[[(2R,3S,4S,5R)-3,4-dihydroxy-5-(hydroxymethyl)tetrahydrofuran-2-yl]oxymethyl]tetrahydropyran-3,4,5-triol	C20H28O10	409.15	324.30	NEG
(E)-3-[5-(1,1-dimethylallyl)-4-hydroxy-2-methoxy-phenyl]-1-(4-hydroxyphenyl)prop-2-en-1-one	C21H22O4	337.14	399.90	NEG
2-(3,4-dihydroxyphenyl)-5-hydroxy-7-[(2S,3R,4S,5S,6R)-3,4,5-trihydroxy-6-[[(2R,3R,4R,5R,6S)-3,4,5-trihydroxy-6-methyl-tetrahydropyran-2-yl]oxymethyl]tetrahydropyran-2-yl]oxy-chromen-4-one	C27H30O15	593.15	305.90	NEG
6-[(2R)-2,3-dihydroxy-3-methyl-butyl]-7-hydroxy-chromen-2-one	C14H16O5	247.10	341.60	POS
(E)-5-(1,3-benzodioxol-5-yl)-N-isobutyl-pent-2-enamide	C16H21NO3	276.16	440.10	POS
4-(3-hydroxybutyl)phenol	C10H14O2	165.09	332.40	NEG
6-[4-hydroxy-2-methyl-6-[(2S,3R,4S,5S,6R)-3,4,5-trihydroxy-6-(hydroxymethyl)tetrahydropyran-2-yl]oxy-phenyl]-4-methoxy-pyran-2-one	C19H22O10	409.12	311.20	NEG
3-(3,4-dihydroxyphenyl)-5,7-dihydroxy-6,8-bis(3-methylbut-2-enyl)chromone	C25H26O6	421.16	440.30	NEG
Theophylline	C7H8N4O2	179.06	284.70	NEG
6-[(2E)-3,7-dimethylocta-2,6-dienyl]-7-hydroxy-chromen-2-one	C19H22O3	297.15	513.40	NEG
Aurantiamide acetate	C27H28N2O4	425.18	395.40	NEG
[(2R,3S,4S,5R,6S)-3,4,5-trihydroxy-6-[2-(hydroxymethyl)phenoxy]tetrahydropyran-2-yl]methyl benzoate	C20H22O8	389.13	397.60	NEG
5-Hydroxy-7,8-dimethoxyflavone,Moslosooflavone	C17H14O5	299.09	365.60	POS
Purine	C5H4N4	119.03	48.50	NEG
Norepinephrine	C8H11NO3	170.08	68.60	POS
1,7-Dimethylxanthine	C7H8N4O2	179.06	284.70	NEG
(2S,3S,4S,5R,6S)-3,4,5-trihydroxy-6-(5-hydroxy-4-oxo-2-phenylchromen-7-yl)oxyoxane-2-carboxylic acid	C21H18O10	431.10	264.00	POS
[(2R,3S,4S,5R,6S)-3,4,5-trihydroxy-6-(4-hydroxyphenoxy)oxan-2-yl]methyl (E)-3-(3,4-dihydroxyphenyl)prop-2-enoate	C21H22O10	433.11	306.80	NEG
12-Oxo-phytodienoic acid	C18H28O3	291.20	466.50	NEG
13-OxoODE	C18H30O3	293.21	467.00	NEG
18 beta-Glycyrrhetintic Acid	C30H46O4	469.33	558.10	NEG
2-tert-Butyl-4-methoxyphenol	C11H16O2	179.11	380.60	NEG
2,5-dihydroxy-3-undecylcyclohexa-2,5-diene-1,4-dione	C17H26O4	293.18	452.80	NEG
3-Isomangostin	C24H26O6	409.17	454.70	NEG
4-Ethoxy-4-oxobut-2-enoic acid	C6H8O4	143.04	670.60	NEG
4-Hydroxy-2-methoxybenzaldehyde	C8H8O3	151.04	337.80	NEG
4-Nitrophenol	C6H5NO3	138.02	334.60	NEG
5-[2-(furan-3-yl)ethyl]-8a-(hydroxymethyl)-5,6-dimethyl-3,4,4a,6,7,8-hexahydronaphthalene-1-carboxylic acid	C20H28O4	331.19	437.60	NEG
5-O-Demethylnobiletin	C20H20O8	389.12	277.10	POS

5,7-Dihydroxy-2,6,8-trimethylchromone	C12H12O4	219.07	493.10	NEG
Apigenin-7-O-beta-D-glucoside	C21H20O10	431.10	280.70	NEG
Chrysanthemic Acid	C10H16O2	167.11	409.80	NEG
cis-3-Hydroxy-DL-proline	C5H9NO3	132.07	76.70	POS
Demethyl medicarpin	C15H12O4	255.07	335.70	NEG
Di(2-ethylhexyl)phthalate (DEHP)	C24H38O4	391.28	79.20	POS
Fraxetin	C10H8O5	209.04	244.20	POS
gamma-Glutamyltyrosine	C14H18N2O6	311.12	111.10	POS
Hydroxyvalerenic acid	C15H22O3	249.15	383.00	NEG
Indole-3-carbinol	C9H9NO	146.06	323.40	NEG
Jasmonic acid	C12H18O3	209.12	344.00	NEG
Methylacetate	C3H6O2	73.03	183.80	NEG
methylcinnamate	C10H10O2	163.08	224.70	POS
n-Propyl gallate	C10H12O5	211.06	213.50	NEG
Pinolenic acid	C18H30O2	277.22	652.00	NEG
ponasterone_A	C27H44O6	463.31	450.90	NEG
Prim-O-glucosylcimifugin	C22H28O11	469.17	315.40	POS
Sinensetin	C20H20O7	373.13	394.20	POS
trans-2-Ethoxy-5-(1-propenyl)phenol	C11H14O2	177.09	328.30	NEG
(-)-Jasmonic_acid	C12H18O3	209.12	369.70	NEG
(.+/-.)-2-Phenylpropanoic acid	C9H10O2	149.06	385.20	NEG
(12Z,15Z)-9,10,11-Trihydroxyoctadeca-12,15-dienoic acid	C18H32O5	327.22	405.40	NEG
(2R,3S,4S,5R,6R)-5-[(2S,3R,4R)-3,4-dihydroxy-4-(hydroxymethyl)oxolan-2-yl]oxy-2-(hydroxymethyl)-6-(2-phenylethoxy)oxane-3,4-diol	C19H28O10	434.20	282.60	POS
(3,6,9-trimethylidene-2-oxo-3a,4,5,6a,7,8,9a,9b-octahydroazuleno[4,5-b]furan-8-yl) acetate	C17H20O4	311.13	370.10	POS
(5S,9R)-14-(hydroxymethyl)-5,9-dimethyl-tetracyclo[11.2.1.01,10.04,9]hexadec-14-ene-5-carboxylic acid	C20H30O3	317.21	465.90	NEG
(9Z,12R)-12-Hydroxyoctadec-9-enoic acid	C18H34O3	297.24	495.50	NEG
1-[4-hydroxy-3-(3-methylbut-2-enyl)phenyl]ethanone	C13H16O2	205.12	370.70	POS
2,4,6-Trimethylphenol	C9H12O	137.10	299.80	POS
2'-Hydroxyacetophenone	C8H8O2	135.05	715.10	NEG
3-Indoleacetonitrile	C10H8N2	157.08	209.60	POS
4-HYDROXYCYCLOHEXANECARBOXYLIC ACID	C7H12O3	143.07	307.50	NEG
5-hexyltetrahydrofuran-2-one	C10H18O2	169.12	467.60	NEG
6-[3-[(3,4-dimethoxyphenyl)methyl]-4-methoxy-2-(methoxymethyl)butyl]-4-methoxy-1,3-benzodioxole	C24H32O7	415.21	449.80	POS

8-Prenylnaringenin	C20H20O5	341.14	283.30	POS
9-HODE	C18H32O3	295.23	517.30	NEG
Adynerin	C30H44O7	515.30	555.30	NEG
Bakuchiol	C18H24O	279.17	257.70	POS
Caffeic acid hexoside	C15H18O9	341.09	245.60	NEG
Castanospermine	C8H15NO4	188.09	232.30	NEG
cis-2-Decenoic acid	C10H18O2	169.12	451.10	NEG
cis-7-Hexadecenoic acid	C16H30O2	253.22	563.00	NEG
cis-Melilotoside	C15H18O8	325.09	227.20	NEG
CROTONIC ACID	C4H6O2	85.03	63.30	NEG
Cyclohexanamine	C6H13N	100.11	483.20	POS
Dattelic acid	C16H16O8	335.08	269.70	NEG
Dehydro-1,8-cineole	C10H16O	153.13	238.40	POS
Dehydroabietic_acid	C20H28O2	299.20	520.70	NEG
Diffractaic acid	C20H22O7	375.14	304.50	POS
Dihydrolycorine	C16H19NO4	290.14	375.00	POS
Dipicolinic acid	C7H5NO4	166.01	651.00	NEG
Eupatorin	C18H16O7	345.10	258.20	POS
FA 18:1+3O	C18H34O5	329.23	425.20	NEG
Foliosidine	C16H21NO5	308.15	339.10	POS
Gardenin	C21H22O9	419.13	304.80	POS
Genistein	C15H10O5	271.06	309.60	POS
Gomisin J	C22H28O6	411.18	398.90	POS
Harmalol	C12H12N2O	201.10	291.40	POS
Heneicosanoic_acid	C21H42O2	325.31	622.80	NEG
Loureirin A	C17H18O4	285.11	322.40	NEG
methyl chlorogenate	C17H20O9	369.12	275.70	POS
METHYL GALACTOSIDE	C7H14O6	217.07	43.60	POS
Methyl hexadecanoate	C17H34O2	315.25	472.00	NEG
Methylparaben	C8H8O3	151.04	191.70	NEG
Oxalic acid	C2H2O4	88.99	651.50	NEG
p-Coumaraldehyde	C9H8O2	147.05	104.30	NEG
Phenylacetaldehyde	C8H8O	119.05	277.50	NEG
Pinocembrin	C15H12O4	257.08	288.40	POS

Rhoifolin	C27H30O14	577.16	216.10	NEG
Sinapoylcholine	C16H24NO5	310.16	286.90	POS
Tangeretin	C20H20O7	373.13	357.70	POS
Tetradecanedioic acid	C14H26O4	257.18	439.90	NEG
Tiglic_acid	C5H8O2	99.05	213.20	NEG
Vasicinone	C11H10N2O2	203.08	209.20	POS
.gamma.-Hydroxybutyric acid	C4H8O3	103.04	94.80	NEG
(+)-Costunolide	C15H20O2	233.15	389.30	POS
(S)-11,12,13-Trinor-7-calamenone	C12H14O	175.11	408.20	POS
[6]-Gingerdiol_3,5-diacetate	C21H32O6	379.21	441.30	NEG
1-Aminocyclohexanecarboxylic_acid	C7H13NO2	144.10	99.10	POS
1-O-((3.beta.,5.Xi.,9.Xi.,18.Xi.)-3-((3-O-Hexopyranosylhexopyranuronosyl)oxy)-28-oxoolean-12-en-28-yl)hexopyranose	C48H76O19	955.49	439.20	NEG
2-Methoxy-4-propylphenol	C10H14O2	167.11	239.50	POS
2,6-Dimethoxy-4-(1-propenyl)phenol	C11H14O3	195.10	347.20	POS
2,6-Dimethyl-1,4-benzenediol	C8H10O2	139.08	271.60	POS
3-O-p-Coumaroylquinic acid	C16H18O8	337.09	260.00	NEG
Abyssinone_IV	C25H28O4	393.21	458.50	POS
Albafuran_B	C24H26O4	379.19	464.30	POS
alpha-licanic_acid	C18H28O3	293.21	403.70	POS
Betonicine	C7H13NO3	160.10	214.80	POS
Bilobalide	C15H18O8	325.09	282.80	NEG
Capillone	C12H12O	173.10	422.80	POS
Daidzin	C21H20O9	451.08	269.40	NEG
Di-n-butyl phthalate	C16H22O4	279.16	479.00	POS
Dihydropinosylvin	C14H14O2	215.11	481.60	POS
Ephedrine	C10H15NO	166.12	222.80	POS
Ethyl gallate	C9H10O5	197.05	237.10	NEG
Etoposide	C29H32O13	589.19	284.30	POS
Eugenitin	C12H12O4	221.08	256.90	POS
Fraxidin	C11H10O5	221.05	282.20	NEG
Hypocrellin B	C30H26O10	527.13	271.20	NEG
Isoacoramone	C12H16O4	225.11	379.80	POS
Isorhamnetin	C16H12O7	315.05	368.40	NEG
Jineol	C9H7NO2	162.05	221.10	POS

KOBUSONE	C14H22O2	221.15	432.60	NEG
Monomethyl phthalate	C9H8O4	179.03	293.70	NEG
Morphine N-oxide	C17H19NO4	302.14	381.80	POS
N-Methylbenzamide	C8H9NO	136.08	75.80	POS
Nalidixic acid	C12H12N2O3	233.09	276.80	POS
Orsellinic acid	C8H8O4	167.03	203.40	NEG
Osmorhizole	C11H14O2	179.11	336.10	POS
Pechueloic Acid	C15H20O3	247.13	436.80	NEG
Piperidine	C5H11N	86.10	72.30	POS
Prexanthoperol	C20H26O3	315.20	433.90	POS
Pterosin A	C15H20O3	249.15	375.30	POS
Reynoutrin	C20H18O11	433.08	292.90	NEG
Salicylic acid ethyl ester	C9H10O3	165.06	313.80	NEG
salsolinol	C10H13NO2	180.10	346.00	POS
Schisandrol B	C23H28O7	434.22	403.20	POS
Seneciphylline	C18H23NO5	334.17	340.40	POS
Tuberonic acid	C12H18O4	225.11	272.10	NEG
1,2,4-Benzenetriol	C6H6O3	125.02	85.10	NEG
2-Pyrrolidinone	C4H7NO	86.06	45.90	POS
3-Methyl-4-phenyl-3-buten-2-one	C11H12O	161.10	457.70	POS
3-Methylindole	C9H9N	132.08	209.20	POS
Ambrosic acid	C15H20O4	263.13	402.20	NEG
Atractylenolide I	C15H18O2	231.14	357.50	POS
batatasin III	C15H16O3	243.10	376.30	NEG
Cantleyine	C11H13NO3	208.10	277.10	POS
Celereoin	C14H14O5	263.09	308.20	POS
colchicine	C22H25NO6	400.17	314.50	POS
DESMETHYLDIHYDROCAPSAICIN	C17H27NO3	294.21	458.10	POS
Erosone	C20H16O6	353.10	373.70	POS
Ethyl ferulate	C12H14O4	221.08	382.10	NEG
Ethyl p-coumarate	C11H12O3	191.07	343.70	NEG
Fraxin	C16H18O10	369.08	243.00	NEG
heptan-2-one	C7H14O	115.11	126.80	POS
Isoimperatorin	C16H14O4	271.10	327.90	POS

L-1,2,3,4-Tetrahydro-beta-carboline-3-carboxylic_acid	C12H12N2O2	217.10	247.10	POS
Leucodin	C15H18O3	247.13	375.50	POS
METHIONAL	C4H8OS	105.04	272.40	POS
methyl 4-hydroxy-3,5-dimethoxybenzoate	C10H12O5	213.08	38.30	POS
Mulberrofuran_A	C25H28O4	393.21	469.10	POS
O-Methylcorypalline	C12H17NO2	208.13	443.40	POS
Perivine	C20H22N2O3	361.15	286.90	POS
Phytocassane_E	C20H28O3	317.21	457.40	POS
Precocene_I	C12H14O2	191.11	403.60	POS
PSEUDO-ANISATIN	C15H22O6	297.13	320.20	NEG
Shihunine	C12H13NO2	204.10	440.50	POS
Sinapyl_aldehyde	C11H12O4	207.07	326.50	NEG
Swainsonine	C8H15NO3	172.10	259.90	NEG
Valerophenone	C11H14O	163.11	430.80	POS
Visnagin	C13H10O4	231.06	262.60	POS
(2R,3R,4S,5R)-2-(6-((E)-4-hydroxy-3-methylbut-2-enylamino)-9H-purin-9-yl)-5-(hydroxymethyl)tetrahydrofuran-3,4-diol	C15H21N5O5	352.16	261.60	POS
(R)-8-Acetoxycarvotanacetone	C12H18O3	211.13	448.80	POS
1,5-Dihydroxynaphthalene	C10H8O2	161.06	454.70	POS
2-Linoleoyl-1-palmitoyl-sn-glycero-3-phosphoethanolamine	C39H74NO8P	714.51	642.90	NEG
5-O-methylvisammioside	C22H28O10	453.17	343.80	POS
9-Hydroxycalabaxanthone	C24H24O6	407.15	449.80	NEG
Benzoylformic acid	C8H6O3	149.02	389.40	NEG
beta-Asarone	C12H16O3	209.12	323.10	POS
Corylin	C20H16O4	319.10	206.80	NEG
Gitogenin	C27H44O4	433.33	433.50	POS
Isosinensetin	C20H20O7	373.13	328.10	POS
Isoxanthohumol	C21H22O5	353.14	180.50	NEG
L-3-Aminoisobutyric acid	C4H9NO2	104.07	104.70	POS
L-Asarinin	C20H18O6	355.12	373.70	POS
leonurine	C14H21N3O5	334.14	252.20	POS
Methylophiopogonanone A	C19H18O6	341.10	328.30	NEG
Orientin	C21H20O11	445.08	250.20	NEG
Pancratistatin	C14H15NO8	326.09	328.50	POS
Tectorigenin	C16H12O6	301.07	257.70	POS

Xanthorrhizol	C15H22O	217.16	497.70	NEG
(S)-5,7-dihydroxy-8-(3-methylbut-2-en-1-yl)-2-phenylchroman-4-one	C20H20O4	325.14	392.30	POS
[(3aS,4S,5S,6E,10E,11aR)-6-formyl-5-methoxy-10-methyl-3-methylidene-2-oxo-3a,4,5,8,9,11a-hexahydrocyclodeca[b]furan-4-yl] 2-methylpropanoate	C20H26O6	385.16	390.50	POS
1-(2,4,6-Trimethoxyphenyl)-1,3-butanedione	C13H16O5	253.11	293.60	POS
1-Benzylpyrrolidine-3-carboxylic acid	C12H15NO2	206.12	307.90	POS
2-Methyl-3-hydroxybutyric acid	C5H10O3	117.06	106.70	NEG
5-(1-Hydroxyethyl)oxolan-2-one	C6H10O3	131.07	104.60	POS
Benzyl alcohol + Hex-Pen	C18H26O10	401.15	315.40	NEG
Bergamottin	C21H22O4	361.14	313.50	POS
D8'-Merulinic_acid_C	C24H38O3	375.29	436.90	POS
Dihydrocapsaicin	C18H29NO3	306.21	473.10	NEG
Hexose + C13H19O (isomer of 1061)	C19H30O7	415.20	362.00	NEG
hirsutanone	C19H20O5	327.12	284.90	NEG
meso-dihydroguaiaretic acid	C20H26O4	353.17	275.90	POS
Phenylethyl 2-Glucoside	C14H20O6	283.12	348.90	NEG
Precocene II	C13H16O3	221.12	407.60	POS
Procyanidin B1	C30H26O12	579.15	251.50	POS
Rehmannioside A	C21H32O15	547.16	439.30	POS
Salicylaldehyde	C7H6O2	123.04	75.80	POS
Tuberosin	C20H18O5	339.12	351.50	POS
(2S,3R,4S,5S,6R)-2-[4-(2-hydroxyethyl)phenoxy]-6-(hydroxymethyl)oxane-3,4,5-triol	C14H20O7	318.15	233.10	POS
(3S,3aR,4S,4aR,7aR,8R,9aR)-3,4a,8-Trimethyl-2,5-dioxo-2,3,3a,4,4a,5,7a,8,9,9a-decahydroazuleno[6,5-b]furan-4-yl (2Z)-2-methyl-2-butenoate	C20H26O5	369.17	249.70	POS
[(2R,3S,4S,5R,6R)-6-[(2S,3S,4S,5R)-3,4-dihydroxy-2,5-bis(hydroxymethyl)oxolan-2-yl]oxy-3,4,5-trihydroxyoxan-2-yl]methyl 4-hydroxybenzoate	C19H26O13	461.13	203.60	NEG
12(13)-Epoxy-9Z-octadecenoic acid	C18H32O3	297.24	454.70	POS
2,4,5-trimethoxybenzoic acid	C10H12O5	251.03	41.90	POS
4-(2,6,6-Trimethyl-1,3-cyclohexadien-1-yl)-2-butanone	C13H20O	193.16	378.50	POS
9(10)-Epoxy-12Z-octadecenoic acid	C18H32O3	297.24	438.30	POS
9Z,11E,13E-Octadecatrienoic acid	C18H30O2	279.23	456.00	POS
Acetoin	C4H8O2	87.05	426.60	NEG
Alpinetin Methyl Ether	C17H16O4	307.09	243.70	POS
Cincassiol_B	C20H32O8	401.22	395.10	POS

Perilloside_B	C16H24O7	329.16	271.70	POS
Piperonal	C8H6O3	151.04	263.60	POS
Prostaglandin B1	C20H32O4	337.24	393.20	POS
Pterostilbene	C16H16O3	257.11	41.20	POS
SINAPIC ACID	C11H12O5	223.06	359.50	NEG
(1r,3R,4s,5S)-4-{[(2E)-3-(3,4-Dihydroxyphenyl)-2-propenoyl]oxy}-1,3,5-trihydroxycyclohexanecarboxylic acid	C16H18O9	353.09	433.70	NEG
(2Z,6E,10E)-12-hydroxy-6,10-dimethyl-2-(4-methylpent-3-enyl)dodeca-2,6,10-trienoic acid [IIN-based on	C20H32O3	343.23	443.80	POS
(R)-1-((4,8-dimethoxyfuro[2,3-b]quinolin-7-yl)oxy)-3-methylbutane-2,3-diol	C18H21NO6	370.13	351.50	POS
[(9R,10R)-10-acetyloxy-8,8-dimethyl-2-oxo-9,10-dihydropyrano[2,3-f]chromen-9-yl] 3-methylbut-2-enoate [IIN-based on	C21H22O7	425.10	253.40	POS
[2,3-dihydroxy-1-(7-methoxy-2-oxochromen-6-yl)-3-methylbutyl] 3-methylbutanoate [IIN-based	C20H26O7	379.18	232.20	POS
12:4+3O fatty acyl hexoside	C18H28O9	387.17	342.80	NEG
2,4-Pentadienoic acid, 5-[(1R,3S,5R,8S)-3-(beta-D-glucopyranosyloxy)-8-hydroxy-1,5-dimethyl-6-oxabicyclo[3.2.1]oct-8-yl]-3-methyl-, (2Z,4E)-	C21H32O10	445.21	232.10	POS
4-[4-[hydroxy-(4-hydroxy-3-methoxyphenyl)methyl]-3-(hydroxymethyl)oxolan-2-yl]-2-methoxyphenol	C20H24O7	375.14	279.20	NEG
4-Hydroxyphenylpyruvic acid	C9H8O4	179.04	324.80	NEG
5-O-Methylembelin	C18H28O4	309.21	416.00	POS
alpha-Asarone	C12H16O3	209.12	402.10	POS
Anisodamine	C17H23NO4	306.17	424.00	POS
Eriodictyol-7-O-glucoside	C21H22O11	449.11	320.90	NEG
Erlose	C18H32O16	503.16	75.00	NEG
isomer of dihydrophaseic acid	C15H22O5	283.15	216.70	POS
isookanin-7-O-glucoside	C21H22O11	451.12	337.00	POS
Matsutakic_acid_A	C10H16O4	201.11	301.50	POS
Methyl Heptadecanoic acid	C18H36O2	283.26	475.20	NEG
PTERYXIN	C21H22O7	387.14	339.10	POS
(+)-trans-Chrysanthemic acid	C10H16O2	169.12	274.10	POS
1-Monolinolenin	C21H36O4	353.27	464.30	POS
1-Naphthalenecarboxylic acid, 5-[2-(2,5-dihydro-2-oxo-3-furanyl)ethyl]decahydro-1,4a-dimethyl-6-methylene-, methyl ester	C21H30O4	369.21	446.00	POS
10-Hydroxycamptothecin	C20H16N2O5	365.12	324.80	POS
2-[(2r)-9-hydroxy-7-oxo-2,3-dihydro-7h-furo[3,2-g]chromen-2-yl]propan-2-yl |A-d-glucopyranoside	C20H24O10	423.13	299.40	NEG
4'-O-Methylsigmoidin	C21H22O6	369.14	397.20	NEG
Eupatilin	C18H16O7	343.09	354.10	NEG
Flavone base + 4O, C-(dehydro-dHex)-dHex	C27H28O14	575.14	298.50	NEG
Geissoschizine methyl ether	C22H26N2O3	389.18	308.90	POS

Isobutyric acid	C4H8O2	89.06	77.70	POS
L-DOPA methyl ester	C10H13NO4	210.08	257.40	NEG
Maclurin	C13H10O6	261.04	206.80	NEG
neoandrographolide	C26H40O8	503.26	245.10	POS
Oregonin	C24H30O10	477.18	410.80	NEG
Pseudorhodomyrtoxin	C24H28O7	429.19	413.50	POS
(2R,3R)-2-(2,6-dihydroxyphenyl)-3,5,7-trihydroxy-2,3-dihydrochromen-4-one	C15H12O7	303.05	95.70	NEG
(E)-10-Oxo-8-decenoic_acid	C10H16O3	185.12	278.10	POS
1-Phenanthrenecarboxylic_acid,_7-ethenyl-1,2,3,4,4a,4b,5,6,7,9,10,10a-dodecahydro-9-hydroxy-1,4a,7-trimethyl-_	C20H30O3	341.21	216.50	POS
2,6-Di-tert-butyl-1,4-benzenediol	C14H22O2	223.17	399.70	POS
8-Desoxygartanin	C23H24O5	379.16	552.90	NEG
Aloesol	C13H14O4	235.10	363.40	POS
Arctiin	C27H34O11	579.21	288.30	NEG
Cinnamyl_acetate	C11H12O2	177.09	408.20	POS
cis-Aconitic acid	C6H6O6	173.01	121.80	NEG
Citronellyl_acetate	C12H22O2	199.17	330.20	POS
Dipropyl phthalate	C14H18O4	251.13	356.30	POS
Methyl 3,4,5-trimethoxycinnamate	C13H16O5	253.11	312.80	POS
Methyl_farnesoate	C16H26O2	251.20	428.70	POS
Paprazine	C17H17NO3	306.11	107.20	POS
Piperic Acid	C12H10O4	219.07	342.70	POS
Quercetin 7-rhamnoside	C21H20O11	449.10	289.90	POS
Rottlerin	C30H28O8	517.19	416.80	POS
Santonin	C15H18O3	245.12	403.30	NEG
Saponarin	C27H30O15	593.15	252.70	NEG
swertiamarin	C16H22O10	373.11	216.40	NEG
Tracheloside	C27H34O12	595.20	308.90	NEG
Triptonide	C20H22O6	359.15	277.50	POS
Xanthohumol	C21H22O5	377.14	245.90	POS
4-(4-Hydroxyphenyl)-2-butanone	C10H12O2	163.08	429.80	NEG
9-oxo-nonanoic_acid	C9H16O3	173.12	396.80	POS
Abieta-8(14),9(11),12-triene-7,18-diol	C20H30O2	325.22	481.60	POS
Nodakenin	C20H24O9	409.15	337.80	POS
Peperinic_acid	C10H14O3	183.10	264.80	POS

Pisiferal	C20H28O2	301.22	441.30	POS
(3beta,6beta)-Furanoeremophilane-3,6-diol_6-acetate	C17H24O4	293.17	438.50	POS
(Z)-2-Nonen-1-ol	C9H18O	143.14	453.00	POS
2-[1-hydroxy-1-(4-methoxyphenyl)propan-2-yl]oxy-6-(hydroxymethyl)oxane-3,4,5-triol	C16H24O8	343.14	284.60	NEG
2-Hydroxy-4-methoxybenzoic acid	C8H8O4	169.05	239.20	POS
2-Phenylethyl_3-methylbutanoate	C13H18O2	207.14	282.20	POS
3-Hexanone	C6H12O	83.09	128.60	POS
Curdione	C15H24O2	237.18	440.60	POS
Isoyatein	C22H24O7	401.16	397.60	POS
Mangiferin	C19H18O11	421.08	44.00	NEG
Methylophiopogonanone B	C19H20O5	329.14	55.80	POS
Moracin_L	C19H16O5	325.11	236.70	POS
nortrachelogenin	C20H22O7	392.17	384.50	POS
Sweroside	C16H22O9	359.14	234.60	POS
trans-Zeatin	C10H13N5O	218.10	183.40	NEG
(S)-4-(4-Methylphenyl)-2-pentanone	C12H16O	177.13	394.20	POS
alpha-santonin	C15H18O3	247.13	84.90	POS
Amidosulfonic_acid	H3NO3S	97.99	38.70	POS
Amygdalin	C20H27NO11	458.17	320.00	POS
Coixol	C8H7NO3	166.05	38.00	POS
decyl acetate	C12H24O2	223.17	421.60	POS
Desacetylvindoline	C23H30N2O5	415.22	232.10	POS
licoflavanone	C20H20O5	341.14	255.60	POS
magnolol	C18H18O2	289.12	372.60	POS
Methyl 2-{[6-O-(beta-D-xylopyranosyl)-beta-D-glucopyranosyl]oxy}benzoate	C19H26O12	464.18	251.80	POS
Tinnevellin glucoside	C20H24O9	407.13	341.80	NEG
trans-2-Hexenal	C6H10O	99.08	666.80	POS
Warfarin	C19H16O4	307.10	362.90	NEG
.beta.-Damascone	C13H20O	193.16	397.50	POS
(S)-Edulinine	C16H21NO4	292.15	347.20	POS
3'-Demethylnobiletin	C20H20O8	389.12	309.90	POS
5-hydroxy-2,2-dimethyl-10-(2-methylbut-3-en-2-yl)pyrano[3,2-g]chromen-8-one	C19H20O4	313.14	422.80	POS
Benzoic acid + 2O, O-Hex	C13H16O9	315.07	94.80	NEG
Chaulmoogric Acid	C18H32O2	298.27	424.20	POS

Dihydrocoumaroyl Hexoside	C15H20O8	327.11	238.20	NEG
N-Tris(hydroxymethyl)methylglycine	C6H13NO5	180.09	74.90	POS
Prenyl_caffeate	C14H16O4	249.11	379.90	POS
Vulgarole	C12H20O3	213.15	383.70	POS
(-)-Curcumol	C15H24O2	237.18	417.70	POS
12-Ketoporrigenin	C27H42O5	447.31	430.40	POS
2,3-Dihydroxy-1-(4-hydroxy-3-methoxyphenyl)-1-propanone	C10H12O5	213.07	43.30	POS
3,5,6,7,8,3',4'-Heptamethoxyflavone	C22H24O9	433.15	252.60	POS
Auxin_b	C18H30O4	311.22	423.20	POS
Casticin	C19H18O8	373.10	331.70	NEG
Cubebin	C20H20O6	357.13	387.20	POS
forsythoside B	C34H44O19	755.24	251.50	NEG
Gancaonin_C	C20H18O6	355.12	388.90	POS
Harrisonin	C27H32O10	517.20	308.30	POS
Kinetin	C10H9N5O	214.07	103.90	NEG
Lysionotin	C18H16O7	367.08	148.50	POS
plumieride	C21H26O12	469.13	306.20	NEG
Syringic acid	C9H10O5	221.04	46.70	POS
(2S)-6-[(2S)-5,7-dihydroxy-2-(4-hydroxyphenyl)-4-oxo-2,3-dihydrochromen-8-yl]-5,7-dihydroxy-2-(4-hydroxyphenyl)-2,3-dihydrochromen-4-one	C30H22O10	541.12	239.10	NEG
4,7,8-Trimethoxy-2-oxo-1,2-dihydro-3-quinolinecarbaldehyde	C13H13NO5	264.09	271.10	POS
6,7-Dihydrotabersonine	C21H26N2O2	361.19	295.10	POS
candidone	C22H24O4	351.16	419.10	NEG
Dexamethasone acetate	C24H31FO6	433.21	297.70	NEG
Octopamine, N-feruloyl-	C18H19NO5	330.14	260.60	POS
tabersonine	C21H24N2O2	337.19	139.10	POS
2,6-Dimethyl-8-hydroxy-2E,6E-octadienal	C10H16O2	169.12	261.20	POS
angolensin (r)	C16H16O4	295.10	249.70	POS
Gardneramine	C23H28N2O5	413.20	229.50	POS
Hypocrellin A	C30H26O10	545.15	282.60	NEG
Lotaustralin	C11H19NO6	260.11	228.50	NEG
(3R)-3-Hydroxy-L-proline	C5H9NO3	130.05	74.60	NEG
1-hydroxy-4-[(2S,3R,4S,5S,6R)-3,4,5-trihydroxy-6-(hydroxymethyl)oxan-2-yl]oxynaphthalene-2-carboxylic acid	C17H18O9	365.08	263.00	NEG
7-HYDROXY-8,4'-DIMETHOXYISOFLAVONE	C17H14O5	299.09	328.20	POS

Bullatine A	C22H33NO2	344.25	272.70	POS
Gnididin	C37H44O10	649.30	405.30	POS
Methyl gallate	C8H8O5	185.04	64.20	POS
PECTOLINARIN	C29H34O15	623.19	359.10	POS
Triptophenolide	C20H24O3	311.17	527.20	NEG
(1S,2R,4R,8S)-p-Menthane-2,8,9-triol_2-glucoside	C16H30O8	351.20	309.50	POS
(S)-Annocherine_A	C17H15NO4	298.11	357.40	POS
1'-Acetoxychavicol_acetate	C13H14O4	235.10	311.10	POS
Baccatin III	C31H38O11	625.21	384.40	POS
ilicic acid	C15H24O3	253.18	347.90	POS
Sarmentosin	C11H17NO7	276.11	79.30	POS
Withaferin A	C28H38O6	469.26	532.90	NEG
8-(2,3-dihydroxy-3-methylbutyl)-7-methoxychromen-2-one	C15H18O5	296.15	287.70	POS
Capsiate	C18H26O4	307.19	362.40	POS
Huperzine B	C16H20N2O	257.17	415.00	POS
Jasminoside B	C16H26O8	369.15	307.40	POS
Mesaconitine	C33H45NO11	632.30	400.60	POS
Rosin	C15H20O6	341.12	276.00	NEG
(1S,3R,4S,5R)-3,5-bis({[(2E)-3-(3,4-dihydroxyphenyl)prop-2-enoyl]oxy})-1,4-dihydroxycyclohexane-1-carboxylic acid	C25H24O12	539.11	417.10	POS
(3S,7R)-iso-jasmonic_acid	C12H18O3	211.13	291.40	POS
Artesunate	C19H28O8	383.17	303.80	NEG
BENZYL ALCOHOL	C7H8O	107.05	249.60	NEG
Epipinoresinol-4,4'-di-O-.beta.-D-glucopyranoside	C32H42O16	681.24	247.70	NEG
Harmaline	C13H14N2O	215.11	38.00	POS
Pipericine	C22H41NO	336.33	525.90	POS
Acoric acid	C15H24O4	267.16	447.90	NEG
Acridine	C13H9N	180.08	706.90	POS
Armillarilin	C24H30O7	431.21	434.60	POS
Corydine	C20H23NO4	342.18	231.90	POS
forskolin	C22H34O7	411.24	404.70	POS
Myricanol	C21H26O5	357.17	437.60	NEG
Paynantheine	C23H28N2O4	397.21	211.30	POS
valeramide	C5H11NO	102.09	211.90	POS
(+)-Isostearic_acid	C18H36O2	283.26	270.00	NEG

[1-acetyloxy-4-(3-hydroxy-3-methylpent-4-enyl)-3,4,8,8-tetramethyl-1,2,3,5,6,7-hexahydronaphthalen-2-yl]_acetate	C24H38O5	424.31	398.60	POS
Acoramone	C12H16O4	225.11	292.50	POS
Artemisinin	C15H22O5	305.13	52.60	POS
Auxin_a	C18H32O5	329.23	423.10	POS
Ginsenoside Rh2 (S-FORM)	C36H62O8	667.43	602.30	NEG
Harpagoside	C24H30O11	495.19	279.80	POS
Isocurcumenol	C15H22O2	257.15	505.20	POS
Isolupalbigenin	C25H26O5	405.18	232.90	NEG
Kaempferol 3-alpha-L-arabinopyranoside	C20H18O10	441.07	253.20	POS
Robustine	C12H9NO3	216.07	268.30	POS
Tricycloekasantal	C12H18O	179.14	338.20	POS
Usaramine	C18H25NO6	352.17	275.60	POS
Vomicine	C22H24N2O4	381.18	249.30	POS
(9Z,12Z,14E)-16-Hydroxy-9,12,14-octadecatrienoic_acid	C18H30O3	295.23	436.10	POS
14-Benzoylaconine	C32H45NO10	648.30	389.60	NEG
3-[3-[(2E)-3,7-dimethylocta-2,6-dienyl]-4-hydroxyphenyl]-7-hydroxychromen-4-one	C25H26O4	389.18	268.00	NEG
3-O-Caffeoylshikimic_acid	C16H16O8	337.09	48.90	POS
4-[2-(2,6-dimethoxy-4-prop-2-enylphenoxy)-1-hydroxypropyl]-2-methoxyphenol	C21H26O6	413.14	270.20	POS
Bruceine D	C20H26O9	409.15	298.10	NEG
Cryptotanshinone	C19H20O3	295.13	420.70	NEG
DEXCHLORPHENIRAMINE	C16H19ClN2	273.11	453.00	NEG
Engeletin	C21H22O10	435.13	345.90	POS
(5R,5aR,8aR,9R)-5-hydroxy-9-(3,4,5-trimethoxyphenyl)-5a,6,8a,9-tetrahydro-5H-[2]benzofuro[5,6-f][1,3]benzodioxol-8-one [IIN-based on	C22H22O8	397.13	384.10	POS
(7S,8R,8aS,14aR,14bS)-7,8-Dimethyl-5a,6,7,8,8a,14b-hexahydro-5H-benzo[kl]bis[1,3]dioxolo[4,5-b:4',5'-g]xanthen-5-one	C20H20O6	357.13	316.20	POS
Cadabicilone	C15H22O3	251.16	393.60	POS
Cyperine	C15H16O4	259.10	324.30	NEG
Eudesmin	C22H26O6	387.18	405.30	POS
Jasmolone	C11H16O2	181.12	373.60	POS
maesopsin	C15H12O6	287.05	61.50	NEG
Moracin_O	C19H18O5	327.12	290.30	POS
Moreollin	C35H42O8	591.29	267.60	POS
Nardosinone	C15H22O3	251.16	372.60	POS
Vismione_D	C25H30O5	411.22	458.50	POS

6,8-dihydroxy-2,2,4,4-tetramethyl-7-(3-methylbutanoyl)-9-(2-methylpropyl)-9H-xanthene-1,3-dione	C26H34O6	443.25	292.50	POS
DUBINIDINE	C15H17NO4	276.12	225.10	POS
Glabrolide	C30H44O4	467.31	388.10	NEG
Hexose + C13H17O3	C19H28O9	399.17	246.30	NEG
Icariside_II	C27H30O10	515.19	318.70	POS
Khelloside	C19H20O10	407.10	294.70	NEG
Reserpiline	C22H26N2O4	381.18	265.10	NEG
Yangambin	C24H30O8	469.19	369.50	POS
(+)-Abscisic acid	C15H20O4	265.14	389.30	POS
(1aS,10aR)-1a,5,9-Trimethyl-1a,3,6,10a-tetrahydrooxireno[4,5]cyclodeca[1,2-b]furan-10(2H)-one	C15H18O3	229.12	362.70	POS
2-amino-3-(5-hydroxy-1H-indol-3-yl)propanoic acid	C19H22N2O	221.09	228.50	POS
3-Methylhexahydropyrrolo[1,2-a]pyrazine-1,4-dione	C8H12N2O2	169.10	40.70	POS
Phorbol	C20H28O6	363.18	436.50	NEG
1-Naphthalenepentanoic acid, 5-carboxydecahydro-beta,5,8a-trimethyl-2-methylene-	C20H32O4	359.22	417.70	POS
Beiwutine	C33H45NO12	648.29	419.40	POS
Camptothecin	C20H16N2O4	347.10	439.90	NEG
Dehydrodiisoeugenol	C20H22O4	325.15	439.00	NEG
2,3',4,6-Tetrahydroxybenzophenone	C13H10O5	245.04	47.60	NEG
Isoflavanone base + 4O, 1Prenyl	C20H20O6	357.13	354.00	POS
Isopentenyladenine	C10H13N5	202.11	279.20	NEG
Obacunone	C26H30O7	477.20	256.50	POS
8-(2-hydroxy-3-methylbut-3-enyl)-7-methoxychromen-2-one	C15H16O4	278.14	224.70	POS
alpha-Methylene-gamma-butyrolactone	C5H6O2	99.04	100.00	POS
Damascenine	C10H13NO3	196.10	242.50	POS
Gomisin D	C28H34O10	553.21	274.10	POS
Myristicinaldehyde	C9H8O4	181.05	314.10	POS
S-Furanopetasitin	C24H32O5S	433.21	264.70	POS
arctigenin	C21H24O6	390.19	400.10	POS
asperuloside	C18H22O11	413.11	280.90	NEG
C18:4n-3,5,7,9	C18H28O2	277.22	411.40	POS
Cyasterone	C29H44O8	565.30	400.20	NEG
Isorhynchophylline	C22H28N2O4	383.19	233.20	NEG
peiminine	C27H43NO3	430.33	458.10	POS
Sinomenine	C19H23NO4	328.15	226.50	NEG

verproside	C22H26O13	497.13	256.30	NEG
2'-Hydroxy-3',4',6',3,4-pentamethoxychalcone	C20H22O7	375.14	387.20	POS
Berbamunine	C36H40N2O6	597.30	277.20	POS
Qunoline-8-methanol	C10H9NO	160.08	209.60	POS
Samaderin_A	C18H18O6	331.12	337.00	POS
SORBIC ACID	C6H8O2	113.06	102.90	POS
13,14-Dimethoxy-6,7-dimethyl-5,6,7,8-tetrahydro[1,3]benzodioxolo[5',6':3,4]cycloocta[1,2-f][1,3]benzodioxole	C22H24O6	402.19	391.40	POS
Amphetamine	C9H13N	158.09	44.60	POS
Coriandrone_A	C16H20O5	293.14	375.90	POS
Cratoxylone	C24H28O7	427.18	394.50	NEG
Curvularin	C16H20O5	291.12	462.20	NEG
Ginkgotoxin	C9H13NO3	184.09	43.00	POS
Rutarin	C20H24O10	463.11	209.20	POS
Zizybeoside_I	C19H28O11	433.17	249.60	POS
5a,6a-Epoxy-7E-megastigmene-3b,9e-diol_9-glucoside	C19H32O8	389.22	300.20	POS
gitoxigenin	C23H34O5	413.23	470.80	POS
Pogostone	C12H16O4	223.10	329.60	NEG
Rutacultin	C16H18O4	275.13	452.20	POS
1-Methoxy-3-carbaldehyde	C10H9NO2	174.06	207.70	NEG
6-methoxy-2-phenyl-4H-chromen-4-one	C16H12O3	275.07	49.00	POS
Acetyllycopsamine	C17H27NO6	342.19	353.20	POS
Azadirachtin	C35H44O16	721.28	249.70	POS
echinacoside	C35H46O20	785.25	247.20	NEG
Karakoline	C22H35NO4	378.26	458.90	POS
Lophophorine	C13H17NO3	236.13	394.70	POS
Rhodinyl Acetate	C12H22O2	197.15	474.50	NEG
(3S,4R,4aS)-3-[(2S,3R,4S,5S,6R)-6-[[(2R,3R,4R)-3,4-dihydroxy-4-(hydroxymethyl)oxolan-2-yl]oxymethyl]-3,4,5-trihydroxyoxan-2-yl]oxy-4-ethenyl-4,4a,5,6-tetrahydro-3H-pyrano[3,4-c]pyran-8-one	C21H30O13	489.16	238.60	NEG
Anofinic_acid	C12H12O3	205.09	424.60	POS
epiyangambin	C24H30O8	445.19	406.80	NEG
Lacinilene_C_7-methyl_ether	C16H20O3	261.15	402.30	POS
picein	C14H18O7	297.10	308.80	NEG
1-O-trans-cinnamoyl-beta-D-glucopyranose	C15H18O7	328.14	121.10	POS
Cyclo(-L-Ser-L-Tyr)	C12H14N2O4	249.09	244.80	NEG

Lobetyolin	C20H28O8	441.18	378.90	NEG
(2S)-5,7-Dimethoxy-3',4'-methylenedioxyflavanone	C18H16O6	329.10	353.60	POS
Benzoic acid + 1O, O-Hex	C13H16O8	299.08	213.70	NEG
Carvyl_acetate	C12H18O2	195.14	341.20	POS
Homoarecoline	C9H15NO2	170.12	280.40	POS
Isoleptospermone	C15H22O4	267.16	377.30	POS
(4aS,8aR,9R,10S,12bR)-10-((S)-Furan-3-yl(hexopyranosyloxy)methyl)-6,6,8a,10-tetramethyl-3,8-dioxodecahydro-3H,6H-spiro[naphtho[1',2':3,4]furo[3,2-c]pyran-9,2'-oxirane]-3'-carboxylic acid	C32H42O14	649.26	277.40	NEG
(5-benzoyloxy-1,2,6-trihydroxycyclohex-3-en-1-yl)methyl benzoate [IIN-based on	C21H20O7	786.28	263.80	POS
[1-(7-methoxy-2-oxochromen-8-yl)-3-methyl-2-oxobutyl] acetate [IIN-based on	C17H18O6	300.13	339.60	POS
Ethyl_hydrogen_fumarate	C6H8O4	145.05	137.70	POS
Grayanotoxin I	C22H36O7	411.24	409.80	NEG
Lupinine	C10H19NO	192.14	381.90	POS
mahanimbine	C23H25NO	354.18	289.40	POS
4-Amino-2-methylenebutanoic_acid	C5H9NO2	116.07	712.00	POS
Acetylpterosin_C	C16H20O4	277.14	435.60	POS
DIBOA + O-Hex	C14H17NO9	342.08	104.40	NEG
Ethyl_p-anisate	C10H12O3	181.09	273.40	POS
Methyl_jasmonate	C13H20O3	225.15	261.20	POS
Physalin_M	C28H32O9	513.22	238.20	POS
(3aR,5S,5aS,9aR)-5,8-dimethyl-1-methylidene-4,5,5a,6,9,9a-hexahydro-3aH-azuleno[6,5-b]furan-2,7-dione	C15H18O3	264.16	412.50	POS
(4R)-4-((1R,3S,5S,7R,9S,10S,12S,13R,14S,17R)-1,3,7,12-tetrahydroxy-10,13-dimethylhexadecahydro-1H-cyclopenta[a]phenanthren-17-yl)pentanoic acid	C24H40O6	407.28	411.00	POS
3'-O-methylbatatasin_III	C16H18O3	259.13	388.30	POS
5,10-dimethoxy-2,2-dimethylpyrano[3,2-g]chromen-8-one	C16H16O5	306.13	285.60	POS
Archangelicine	C24H26O7	425.16	386.50	NEG
CRUSTECDYSONE	C27H44O7	498.34	439.90	POS
Dihydroeuparin	C13H14O3	219.10	337.80	POS
evodiamine	C19H17N3O	304.15	363.10	POS
Laurenobiolide	C17H22O4	291.16	447.60	POS
picroside II	C23H28O13	511.14	360.40	NEG
Solasodine	C27H43NO2	414.34	500.70	POS
[8-[2-(3-methylbutanoyloxy)propan-2-yl]-2-oxo-8,9-dihydrofuro[2,3-h]chromen-9-yl] 3-methylbutanoate	C24H30O7	448.23	439.40	POS
3,7,11,15-tetramethyl-2E,6E,10E,14-hexadecatetraenal	C20H32O	289.25	405.70	POS

Chaetoglobosin_A	C32H36N2O5	529.28	222.00	POS
Deoxynivalenol	C15H20O6	297.13	323.80	POS
geniposide	C17H24O10	406.17	239.90	POS
Hymecromone	C10H8O3	194.08	308.00	POS
Ideain_chloride	C21H21O11	448.10	286.50	NEG
Juglone	C10H6O3	175.04	47.40	POS
Podophyllin Acetate	C24H24O9	455.13	394.90	NEG
Casimiroedine	C21H27N3O6	418.19	63.90	POS
Kanzonol_O	C22H22O6	383.15	396.40	POS
Rotenone	C23H22O6	413.20	383.60	POS
(2E)-3-(1,3-Benzodioxol-5-yl)-1-(pyrrolidin-1-yl)prop-2-en-1-one	C14H15NO3	246.11	329.60	POS
(2S,3R,5R,10R,13R,14S,17S)-2,3,14-trihydroxy-10,13-dimethyl-17-[(2R,3R)-2,3,6-trihydroxy-6-methylheptan-2-yl]-2,3,4,5,9,11,12,15,16,17-decahydro-1H-cyclopenta[a]phenanthren-6-one	C27H44O7	479.30	439.00	NEG
6,4'-Dimethoxyisoflavone-7-glucoside	C23H24O10	505.14	209.80	NEG
7-(2-hydroxypropan-2-yl)-1,4a-dimethyl-2,3,4,5,6,7,8,8a-octahydronaphthalen-1-ol	C15H28O2	239.20	540.50	NEG
bilobol	C21H34O2	341.25	539.70	POS
Galbanic acid	C24H30O5	397.20	467.00	NEG
PRENOL	C5H10O	87.08	71.10	POS
Pterosin_N	C14H18O3	235.13	382.60	POS
Tetrahydropentoxyline	C17H22N2O7	367.15	218.30	POS
Triamcinolone	C21H27FO6	393.17	445.50	NEG
2-Cyclohexen-1-one, 4-[(1E)-3-(.beta.-D-glucopyranosyloxy)-1-buten-1-yl]-4-hydroxy-3,5,5-trimethyl-, (4S)-	C19H30O8	385.19	304.20	NEG
7-[[(1S,4aS,6S,8aR)-6-hydroxy-5,5,8a-trimethyl-2-methylidene-3,4,4a,6,7,8-hexahydro-1H-naphthalen-1-yl]methoxy]chromen-2-one [IIN-based on	C24H30O4	402.19	411.20	POS
Amarogentin	C29H30O13	587.18	297.20	POS
COTARNINE	C12H11NO3	218.08	276.90	POS
Cytochalasin_B	C29H37NO5	480.28	396.80	POS
Dimethyl suberate	C10H18O4	203.13	277.60	POS
Glaucarubin	C25H36O10	479.23	362.60	POS
Sarmentoloside	C29H44O11	569.30	314.60	POS
Triethyl_citrate	C12H20O7	277.13	205.40	POS
Tschimganin	C18H24O4	305.17	355.80	POS
(9R,10R)-9-acetoxy-8,8-dimethyl-2-oxo-2,8,9,10-tetrahydropyrano[2,3-f]chromen-10-yl 3-methylbutanoate	C21H24O7	411.14	273.70	POS
[6]-Shogaol	C17H24O3	275.17	461.70	NEG

5-[3,4,5-trihydroxy-6-(hydroxymethyl)oxan-2-yl]oxy-9-(3,4,5-trimethoxyphenyl)-5a,6,8a,9-tetrahydro-5H-[2]benzofuro[6,5-f][1,3]benzodioxol-8-one	C28H32O13	621.18	277.10	NEG
Buxifoliadine A	C25H29NO4	406.20	284.70	NEG
Cichorioside B	C21H28O10	439.16	323.40	NEG
Mallotochromene	C24H26O8	443.17	421.50	POS
Napelline	C22H33NO3	360.25	458.10	POS
3-Genistein-8-C-glucoside	C21H20O10	450.14	288.40	POS
9-HOTrE	C18H30O3	293.21	534.60	NEG
Benzoic acid + 2O, O-Pen	C12H14O8	285.06	144.20	NEG
Duartin (-)	C18H20O6	331.12	277.60	NEG
Ginsenoyne_E	C17H22O2	259.17	430.80	POS
paulownin	C20H18O7	388.14	373.70	POS
Secobarbital	C12H18N2O3	239.14	109.10	POS
8-Gingerol	C19H30O4	321.21	468.60	NEG
Astringin	C20H22O9	405.12	356.80	NEG
Cassiaoccidentalin_C	C28H30O14	591.17	262.70	POS
Dihydroartemisinin	C15H24O5	283.15	282.80	NEG
Enhydrin	C23H28O10	465.18	276.50	POS
Lentiginosine	C8H15NO2	158.12	216.20	POS
salviaflaside	C24H26O13	521.13	239.10	NEG
Scropolioside D	C34H42O17	767.24	264.20	NEG
Terminaline	C23H41NO2	364.32	430.70	POS
Verbascoside	C29H36O15	623.20	265.40	NEG
(+)-Wikstromol	C20H22O7	373.13	396.40	NEG
3-Hydroxystigmast-5-en-7-one	C29H48O2	429.37	663.50	POS
3,4,5-Trimethoxyphenol	C9H12O4	183.07	281.60	NEG
4,5-Di-O-caffeoylquinic acid methyl ester	C26H26O12	529.13	305.30	NEG
Aloin	C21H22O9	436.16	304.40	POS
Corynoline	C21H21NO5	368.16	268.50	POS
Erythronolactone	C4H6O4	117.02	60.30	NEG
Kurarinone	C26H30O6	437.20	446.10	NEG
Limonexic_acid	C26H30O10	503.19	256.10	POS
N-[2-(4-Methoxyphenyl)ethyl]-3-methyl-2-butenamide	C14H19NO2	234.15	350.30	POS
Vinylphenol O-[L-Rhamnopyranosyl-(1-6)-.beta.-D-glucopyranoside	C20H28O10	427.16	302.50	NEG

(4S,5Z,6S)-4-(2-Methoxy-2-oxoethyl)-5-[2-[(Z)-3-phenylprop-2-enoyl]oxyethylidene]-6-[(2S,3R,4S,5S,6R)-3,4,5-trihydroxy-6-(hydroxymethyl)oxan-2-yl]oxy-4H-pyran-3-carboxylic acid	C26H30O13	549.16	281.40	NEG
[(2R,3S,4R,5R,6R)-6-[2-(3,4-dihydroxyphenyl)ethoxy]-4,5-dihydroxy-2-[[(2R,3R,4R,5R,6S)-3,4,5-trihydroxy-6-methyloxan-2-yl]oxymethyl]oxan-3-yl] (E)-3-(3,4-dihydroxyphenyl)prop-2-enoate	C29H36O15	642.24	261.00	POS
[(2R,3S,4S,5R,6S)-3,4,5-trihydroxy-6-(4-hydroxyphenoxy)oxan-2-yl]methyl (E)-3-(1-hydroxy-4-oxocyclohexa-2,5-dien-1-yl)prop-2-enoate	C21H22O10	452.16	329.50	POS
3-Furfuryl 2-pyrrolecarboxylate	C10H9NO3	192.07	105.90	POS
Abruquinone B	C20H22O8	389.12	286.40	NEG
Atisine	C22H33NO2	344.26	438.00	POS
BENZALDEHYDE	C7H6O	107.05	296.00	POS
Dibutyl sebacate	C18H34O4	315.25	456.00	POS
DIHYDROROBINETIN	C15H12O7	303.05	65.80	NEG
Phlorin	C12H16O8	287.08	236.70	NEG
Supinine	C15H25NO4	284.19	275.00	POS
Taxine_A	C35H47NO10	642.33	330.70	POS
(3aS,4S,7aR)-4-Hydroxy-5-[(2S)-5-hydroxy-2-pentanyl]-6-methyl-3-methylene-3a,4,7,7a-tetrahydro-1-benzofuran-2(3H)-one	C15H22O4	289.14	415.60	POS
[(1aS,1bS,2S,5aR,6S,6aS)-6-hydroxy-2-[(2S,3R,4S,5S,6R)-3,4,5-trihydroxy-6-(hydroxymethyl)oxan-2-yl]oxy-2,5a,6,6a-tetrahydro-1bH-oxireno[5,6]cyclopenta[1,3-c]pyran-1a-yl]methyl (E)-3-phenylprop-2-enoate	C24H28O11	491.16	45.60	NEG
2-Hydroxycyclohexyl 2-O-((2E)-3-(4-hydroxyphenyl)prop-2-enoyl)-.beta.-D-glucopyranoside	C21H28O9	423.17	359.80	NEG
3,8-dihydroxy-3,8-dimethyl-5-propan-2-ylidene-1,2,3a,4,7,8a-hexahydroazulen-6-one	C15H24O3	270.21	393.20	POS
Androsin	C15H20O8	327.11	334.60	NEG
Argentine	C23H26N4O3	407.21	341.20	POS
Camaldulenside	C26H32O11	521.20	249.20	POS
CAPSAICIN	C18H27NO3	306.21	372.00	POS
DIBENZOTHIOPHENE	C12H8S	185.04	41.90	POS
Estragole	C10H12O	147.08	355.40	NEG
Fortunellin	C28H32O14	637.18	262.10	NEG
Isomucronulatol 7-O-glucoside	C23H28O10	463.16	286.00	NEG
L-cis-4-(Hydroxymethyl)-2-pyrrolidinecarboxylic_acid	C6H11NO3	146.08	100.20	POS
Mulberroside A	C26H32O14	567.17	268.20	NEG
Protostemotinine	C23H29NO6	416.20	335.20	POS
S(8-8)S hexoside	C28H36O13	579.21	276.80	NEG
(2R,3S,4S,5R,6S)-2-(hydroxymethyl)-6-[4-[(E)-3-hydroxyprop-1-enyl]phenoxy]oxane-3,4,5-triol	C15H20O7	311.11	269.60	NEG
(2S,3R,4S,5S,6R)-2-[[(1S,4aR,7aS)-7-(hydroxymethyl)-1,4a,5,7a-tetrahydrocyclopenta[c]pyran-1-yl]oxy]-6-(hydroxymethyl)oxane-3,4,5-triol	C15H22O8	329.12	250.30	NEG

(4,7,7-trimethyl-3-bicyclo[2.2.1]heptanyl) (E)-3-(4-hydroxy-3-methoxyphenyl)prop-2-enoate	C20H26O4	348.22	455.10	POS
2,4-dihydroxyheptadec-16-enyl acetate	C19H36O4	327.25	517.80	NEG
5-[(2R,3S)-6-hydroxy-2-(4-hydroxyphenyl)-4-[(E)-2-(4-hydroxyphenyl)ethenyl]-2,3-dihydro-1-benzofuran-3-yl]benzene-1,3-diol	C28H22O6	472.18	322.50	POS
5-Methoxy-1,7-diphenyl-3-heptanone	C20H24O2	297.18	441.50	POS
aschantin	C22H24O7	399.14	385.40	NEG
Avocadyne Acetate	C19H34O4	325.24	503.50	NEG
beta-D-Glucopyranoside, (3Z)-3-hexen-1-yl	C12H22O6	280.17	265.50	POS
Deacetylasperuloside acid methyl ester	C17H24O11	403.10	359.80	NEG
Dihydrocucurbitacin F	C30H48O7	519.33	576.30	NEG
Eleganin	C22H26O9	435.17	303.40	POS
Evoxine	C18H21NO6	348.14	320.70	POS
gigantol	C16H18O4	275.13	413.60	POS
Hippeastrine	C17H17NO5	316.12	331.90	POS
ISOPALMITIC ACID	C16H32O2	274.27	428.30	POS
Palmidin_C	C30H22O7	495.14	356.10	POS
Picraquassioside A	C18H22O10	397.11	295.40	NEG
Piscerythramine	C26H29NO6	452.21	243.90	POS
Quillaic acid	C30H46O5	487.35	328.10	POS
Scopolamine	C17H21NO4	304.15	448.60	POS
Tuberostemonine	C22H33NO4	376.25	454.70	POS
4-[(2R,3R)-3-[(3,4-dimethoxyphenyl)methyl]-4-methoxy-2-(methoxymethyl)butyl]-1,2-dimethoxybenzene	C24H34O6	419.24	431.30	POS
Desoxyrhaponticin	C21H24O8	403.14	330.60	NEG
Evodol	C26H28O9	485.18	347.20	POS
skullcapflavone II	C19H18O8	373.09	203.20	NEG
[(1aS,1bS,2S,5aR,6S,6aS)-1a-(hydroxymethyl)-2-[(2S,3R,4S,5S,6R)-3,4,5-trihydroxy-6-(hydroxymethyl)oxan-2-yl]oxy-2,5a,6,6a-tetrahydro-1bH-oxireno[5,6]cyclopenta[1,3-c]pyran-6-yl] (E)-3-(3-hydroxy-4-methoxyphenyl)prop-2-enoate	C25H30O13	537.17	44.40	NEG
6-(1,1-DIMETHYLALLYL)-2-(1-HYDROXY-1-METHYLETHYL)-2,3-DIHYDRO-7H-FURO[3,2-G]CHROMEN-7-ONE	C19H22O4	353.11	333.10	POS
Eriojaposide_A	C24H38O11	503.25	221.50	POS
Salidroside	C14H20O7	301.12	323.00	POS
(2E,4E)-N-(2-methylpropyl)dodeca-2,4-dienamide	C16H29NO	290.19	223.90	POS
[8-[2-(3-methylbutanoyloxy)propan-2-yl]-2-oxo-8,9-dihydrofuro[2,3-h]chromen-9-yl] (Z)-2-methylbut-2-enoate	C24H28O7	467.15	311.60	POS
9-Methoxycamptothecin	C21H18N2O5	377.12	268.30	NEG
Ancistrocladine	C25H29NO4	408.21	284.40	POS
Corynanthin	C21H26N2O3	377.18	233.40	POS

Glomeratose A	C24H34O15	561.18	214.20	NEG
Ipriflavone	C18H16O3	281.11	100.70	POS
(1aS,3R,4R,5S,5aS,8aR,10R,10aS)-3-Hydroxy-3,10-dimethyl-6-methylidene-2,7-dioxododecahydrooxireno[7,8]cyclodeca[1,2-b]furan-4,5-diyl bis(2-methylprop-2-enoate)	C23H28O9	449.18	281.10	POS
1,7-bis(4-hydroxyphenyl)heptane-3,5-diol	C19H24O4	355.13	339.10	POS
Podocarpusflavone A	C31H20O10	551.10	301.10	NEG
trans-Epoxysuccinyl-L-leucylamido(4-guanidino)butane	C15H27N5O5	358.21	227.70	POS
1-Hydroxy-3,6,7-Trimethoxy-2,8-Diprenylxanthone	C26H30O6	461.20	267.70	POS
7-Formyldehydrothalicsimidine	C23H25NO6	412.18	239.00	POS
Calophyllin_B	C18H16O4	297.11	135.70	POS
Neolinustatin	C17H29NO11	446.17	283.50	POS
Pilosanol_A	C29H32O10	541.21	248.80	POS
Pteroside_Z	C21H30O7	395.21	458.10	POS
Aspecioside	C29H42O10	551.29	326.20	POS
beta-D-Glucopyranoside,_4-(hydroxymethyl)-2-methoxyphenyl	C14H20O8	334.15	322.60	POS
trans-Zeatin-riboside	C15H21N5O5	350.15	275.30	NEG
epicatechin gallate	C22H18O10	441.08	73.40	NEG
Icariside I	C27H30O11	529.17	283.60	NEG
mollugin	C17H16O4	307.10	184.50	POS
Procyanidin B2	C30H26O12	581.19	444.00	NEG
roemerine	C18H17NO2	302.12	262.70	POS
Scrorodioside	C32H40O16	679.23	124.60	NEG
Thalicpureine	C22H27NO5	386.20	239.40	POS
Ginkgolide C	C20H24O11	439.12	248.10	NEG
aucubin	C15H22O9	385.09	165.50	POS
Cedrin	C16H14O8	333.06	40.30	NEG
cirsimarin	C23H24O11	475.13	45.60	NEG
Limonin	C26H30O8	515.19	309.60	NEG
PAEDEROSIDE	C18H22O11S	464.12	309.90	POS
Randainol	C18H18O3	283.13	135.70	POS
Rhodojaponin II	C22H34O7	411.22	294.60	POS
Salsolidine	C12H17NO2	246.09	286.50	POS
(+)-Cannabidiol	C21H30O2	337.22	510.90	POS

5-hydroxy-9-(4-hydroxy-3,5-dimethoxyphenyl)-5a,6,8a,9-tetrahydro-5H-[2]benzofuro[5,6-f][1,3]benzodioxol-8-one [IIN-based on	C21H20O8	383.12	44.70	POS
Gancaonin_E	C25H28O6	425.20	269.30	POS
Verminoside	C24H28O13	525.17	73.70	POS
(6S)-4-Methoxy-6-(2-phenylethyl)-5,6-dihydro-2H-pyran-2-one	C14H16O3	255.10	46.30	POS
Isomucronulatol	C17H18O5	301.11	421.20	NEG
O-methylisopiline	C19H21NO3	312.13	111.10	POS
Oxypaeoniflorin	C23H28O12	495.14	398.30	NEG
Pinoresinol dimethyl ether	C22H26O6	387.19	43.30	POS
Rutaevin	C26H30O9	485.19	262.50	NEG
(2S,3S)-2-(3,4,5-trihydroxyphenyl)-3,4-dihydro-2H-chromene-3,5,7-triol	C15H14O7	305.13	143.90	NEG
5-O-methyllicoricidin	C27H34O5	477.21	264.20	POS
b-D-fructosyl-a-D-(6-O-(E))-feruloylglucoside	C21H28O12	473.16	283.50	POS
Bifurcose	C24H42O21	667.23	210.50	POS
Helenine	C15H20O2	255.14	481.60	POS
Tenuifoliside A	C31H38O17	681.21	95.40	NEG
[(1aS,1bS,2S,5aR,6S,6aS)-1a-(hydroxymethyl)-2-[(2S,3R,4S,5S,6R)-3,4,5-trihydroxy-6-(hydroxymethyl)oxan-2-yl]oxy-2,5a,6,6a-tetrahydro-1bH-oxireno[5,6]cyclopenta[1,3-c]pyran-6-yl] (E)-3-(4-hydroxy-3-methoxyphenyl)prop-2-enoate	C25H30O13	537.17	98.50	NEG
[4]-Gingerdiol_3,5-diacetate	C19H28O6	351.18	470.50	NEG
Narceine	C23H27NO8	446.17	280.60	POS
Suspensaside	C29H36O16	641.20	321.20	POS
(S)-3-(4-hydroxyphenyl)chroman-7-ol	C15H14O3	265.08	294.50	POS
1,4-Naphthoquinone	C10H6O2	159.03	38.00	POS
Benzoylmesaconine	C31H43NO10	590.30	306.60	POS
Epigallocatechin-3-Monogallate	C22H18O11	457.15	417.20	NEG
Propanoic acid, 2-methyl-, (3aS,4S,5S,6E,10E,11aR)-6-formyl-2,3,3a,4,5,8,9,11a-octahydro-5-methoxy-10-methyl-3-methylene-2-oxocyclodeca[b]furan-4-yl ester	C20H26O6	380.20	261.20	POS
4-[(3S,3aR,6S,6aR)-6-(3,4-dimethoxyphenyl)-1,3,3a,4,6,6a-hexahydrofuro[3,4-c]furan-3-yl]-2-methoxyphenol	C21H24O6	414.20	247.10	POS
ANETHOLE	C10H12O	171.08	436.40	POS
Gedunin	C28H34O7	481.23	300.70	NEG
Laurinterol	C15H19BrO	295.07	81.10	POS
Leiocarposide	C27H34O16	613.18	415.80	NEG
Phellamurin	C26H30O11	517.18	218.10	NEG
(6aR,12aR)-6a,12a-Dihydro-6H-[1,3]dioxolo[5,6][1]benzofuro[3,2-c]chromen-3-yl_6-O-(carboxyacetyl)-beta-D-glucopyranoside	C25H24O13	550.16	108.10	POS

1,4a-dimethyl-9-oxo-7-propan-2-yl-3,4,10,10a-tetrahydro-2H-phenanthrene-1-carboxylic acid	C20H26O3	332.22	235.10	POS
Beta-Peltatin	C22H22O8	432.17	49.30	POS
Pesticide3_Propoxur_C11H15NO3_Baygon	C11H15NO3	232.10	301.80	POS
Sauchinone	C20H20O6	357.13	240.70	POS
Agarotetrol	C17H18O6	319.12	259.00	POS
Marchantin J	C29H26O7	487.18	315.40	POS
Picropodophyllotoxin	C22H22O8	432.17	95.00	POS
Piperolactam A	C16H11NO3	264.06	314.30	NEG
12a-Hydroxypachyrrhizone	C20H14O8	383.08	236.50	POS
GRISEOFULVIN	C17H17ClO6	353.08	227.00	POS
Plumbagin	C11H8O3	189.05	42.20	POS
Rheic acid	C15H8O6	283.03	38.50	NEG
astilbin	C21H22O11	468.14	263.40	POS
Broussoflavan_A	C25H30O6	427.22	237.30	POS
Sulindac	C20H17FO3S	355.08	409.70	NEG
Cymarin	C30H44O9	549.30	303.60	POS

Name: The identification of substances through qualitative analysis using tandem mass spectrometry matching; MZ: The median mass-to-charge ratio represents the mass-to-charge ratio of the peak in all sample; RT: Retaining the median retention time signifies the retention time of the peak across all samples.
