# Supplementary material for: Dietary Angelica sinensis Enhances Sow Lactation and Piglet Development Through Gut Microbiota and Metabolism
Source: Vet Sci. 2025 Apr 15;12(4):370. doi: 10.3390/vetsci12040370 (PMC12030784; doi:10.3390/vetsci12040370)
Supplement: Supplementary file 1 [file vetsci-12-00370-s001.zip › Supplementary Table 2.pdf]

Supplementary Table 2. Differentially upregulated metabolites

Name	Formula	MZ	RT	type	P-VALUE	LOG_FOLD CHANGE
2',4',6'-Trihydroxyacetophenone	C8H8O4	149.02	304.00	NEG	0.005	11.507
Coniferaldehyde	C10H10O3	177.06	305.30	NEG	0.007	9.911
4-hydroxy-3-(3-methylbut-2-enyl)benzoic acid	C12H14O3	205.09	382.70	NEG	0.042	9.782
5-[6-(3-hydroxy-4-methoxyphenyl)-1,3,3a,4,6,6a-hexahydrofuro[3,4-c]furan-3-yl]-2-methoxyphenol	C20H22O6	357.13	404.30	NEG	0.011	7.880
Plantamajoside	C29H36O16	639.19	354.60	NEG	0.017	7.151
4-Chromanone	C9H8O2	149.06	303.00	POS	0.010	6.997
3-Butylidenephthalide	C12H12O2	189.09	312.60	POS	0.042	5.894
(2R,3R,4S,5S,6R)-2-benzyloxy-6-[[(2R,3R,4R)-3,4-dihydroxy-4-(hydroxymethyl)tetrahydrofuran-2-yl]oxymethyl]tetrahydropyran-3,4,5-triol	C18H26O10	401.14	239.70	NEG	0.022	4.996
(2R,3R,4S,5S,6R)-2-benzyloxy-6-[[(2S,3R,4S,5R)-3,4,5-trihydroxytetrahydropyran-2-yl]oxymethyl]tetrahydropyran-3,4,5-triol	C18H26O10	401.14	239.70	NEG	0.022	4.996
(2R,3R,4S,5S,6R)-2-benzyloxy-6-[[(2S,3R,4S,5S)-3,4,5-trihydroxytetrahydropyran-2-yl]oxymethyl]tetrahydropyran-3,4,5-triol	C18H26O10	401.14	239.70	NEG	0.022	4.996
3-Hydroxy-carbofuran	C12H15NO4	238.11	307.40	POS	0.000	4.791
Dillapiole	C12H14O4	223.10	353.60	POS	0.039	4.671
(E)-3-[4-[(2S,3R,4S,5S,6R)-3,4,5-trihydroxy-6-(hydroxymethyl)oxan-2-yl]oxyphenyl]prop-2-enoic acid	C15H18O8	344.13	236.00	POS	0.036	4.634
Ganolucidic_acid_A	C30H44O6	501.32	372.50	POS	0.018	3.359
Gemichalcone_C	C30H28O9	533.18	345.00	POS	0.016	3.348
Genipin-gentiobioside	C23H34O15	549.18	389.40	NEG	0.005	3.159
2'-Hydroxy-4',6'-dimethoxy-3'-methylacetophenone	C11H14O4	211.10	310.30	POS	0.001	3.123
(E)-Resveratroloside	C20H22O9	424.16	330.10	POS	0.001	3.033
4-[5-(4-hydroxy-3-methoxyphenyl)-3,4-dimethyloxolan-2-yl]-2-methoxyphenol	C20H24O5	383.13	296.10	POS	0.025	2.743
Protostemonine	C23H31NO6	418.22	352.70	POS	0.013	2.271
2,4-Dimethylcinnamic acid	C11H12O2	175.08	308.00	NEG	0.001	2.269
andrograpanin	C20H30O3	341.21	418.10	POS	0.009	2.250

6,7-Dihydroxy-4-phenylcoumarin	C15H10O4	253.05	288.80	NEG	0.010	2.126
4-Hydroxybenzyl alcohol	C7H8O2	123.05	248.30	NEG	0.038	2.117
Dimethyl (R)-(+)-malate	C6H10O5	163.06	79.80	POS	0.044	1.936
Mesembrinol	C17H25NO3	292.19	371.60	POS	0.004	1.811
7-hydroxy-3-(4-hydroxyphenyl)-5-methoxy-chromen-4-one	C16H12O5	285.08	335.70	POS	0.048	1.803
Artemetin	C20H20O8	387.11	312.00	NEG	0.040	1.803
(E)-Ethyl p-methoxycinnamate	C12H14O3	189.09	454.70	POS	0.007	1.739
Aloin_A	C21H22O9	417.12	316.80	NEG	0.006	1.691
7-methoxy-6-(1,2,3-trihydroxy-3-methylbutyl)chromen-2-one	C15H18O6	317.09	363.40	POS	0.009	1.632
5-hydroxy-3-(4-hydroxyphenyl)-7-[(2S,3R,4S,5S,6R)-3,4,5-trihydroxy-6-(hydroxymethyl)oxan-2-yl]oxychromen-4-one	C21H20O10	433.11	289.30	POS	0.031	1.631
Pinoresinol	C20H22O6	357.13	333.20	NEG	0.023	1.571
cis-Jasmone	C11H16O	165.13	409.80	POS	0.024	1.566
4-Hydroxy-3-methyl-9,10-dioxo-9,10-dihydroanthracen-2-yl 6-O-.beta.-D-xylopyranosyl-.beta.-D-glucopyranoside	C26H28O13	547.15	287.30	NEG	0.006	1.562
Dimethyl phthalate	C10H10O4	177.05	330.20	POS	0.040	1.547
rescinnamine	C35H42N2O9	633.28	308.30	NEG	0.049	1.512
1-(4-hydroxy-3,5-dimethoxy-phenyl)propan-1-one	C11H14O4	193.09	344.80	POS	0.031	1.498
Asperglaucide	C27H28N2O4	445.22	234.10	POS	0.027	1.452
(-)-Tylocrebrine	C24H27NO4	394.20	437.30	POS	0.036	1.419
Ginkgolide A	C20H24O9	426.18	327.20	POS	0.020	1.400
Arnebinol	C16H20O2	245.15	432.80	POS	0.009	1.294
Calealactone B	C21H26O9	421.15	359.30	NEG	0.038	1.252
(3S,4S)-3-hydroxy-4-[(4-hydroxy-3-methoxy-phenyl)methyl]-3-[[3-methoxy-4-[(2S,3R,4S,5S,6R)-3,4,5-trihydroxy-6-(hydroxymethyl)tetrahydropyran-2-yl]oxy-phenyl]methyl]tetrahydrofuran-2-one	C26H32O12	535.18	275.80	NEG	0.041	1.169
2,4-bis(3-methylbut-2-enyl)-6a,11a-dihydro-6H-benzofuro[3,2-c]chromene-3,9-diol	C25H28O4	391.19	422.50	NEG	0.010	1.044
Anacardic acid diene	C22H32O3	343.23	439.20	NEG	0.007	1.031
Apiosylrhododendrin	C21H32O11	459.19	362.40	NEG	0.047	1.030
Chalcone base + 3O, 1Prenyl	C20H20O4	323.13	394.00	NEG	0.001	1.025

3-[(2Z)-3,7-dimethylocta-2,6-dienyl]-2,4-dihydroxy-6-(2-phenylethyl)benzoic acid	C25H30O4	375.19	457.60	NEG	0.005	1.015
Alnuside A	C24H30O9	461.18	409.20	NEG	0.004	0.975
5,9-dimethyltetracyclo[11.2.1.01,10.04,9]hexadecane-5,14-dicarboxylic acid	C20H30O4	333.21	459.20	NEG	0.012	0.966
Bixin	C25H30O4	393.20	441.20	NEG	0.019	0.963
5-[2-(3-furyl)ethyl]-8-hydroxy-5,6,8a-trimethyl-3,4,4a,6,7,8-hexahydronaphthalene-1-carboxylic acid	C20H28O4	331.19	460.70	NEG	0.040	0.960
Marrubiin	C20H28O4	331.19	460.70	NEG	0.040	0.960
Alpha-Ergocryptine	C32H41N5O5	496.34	486.90	POS	0.020	0.949
2-[[3,4-dihydroxy-4-(hydroxymethyl)tetrahydrofuran-2-yl]oxymethyl]-6-(5-hydroxy-1,7,7-trimethyl-norbornan-2-yl)oxy-tetrahydropyran-3,4,5-triol	C21H36O11	463.22	311.20	NEG	0.037	0.913
Ophiopogonoside A	C21H38O8	419.26	457.70	POS	0.019	0.878
[(2S,3R,4S,5S,6R)-3,4,5-trihydroxy-6-(hydroxymethyl)oxan-2-yl] 4-prop-1-en-2-ylcyclohexene-1-carboxylate	C16H24O7	309.13	371.30	NEG	0.010	0.864
Humulone	C21H30O5	361.20	469.80	NEG	0.021	0.851
Gelsemicine	C20H26N2O4	359.20	457.70	POS	0.016	0.823
Anthranilic acid (Vitamin L1)	C7H7NO2	136.04	285.60	NEG	0.038	0.815
Brucine	C23H26N2O4	395.20	441.80	POS	0.009	0.801
Deoxyloganic acid (Not validated)	C16H24O9	359.14	290.90	NEG	0.043	0.775
Pentose-Hexose + C10H17	C21H36O10	447.22	400.20	NEG	0.005	0.750
Hydroxyacetone	C3H6O2	73.03	61.20	NEG	0.025	0.745
4-hydroxy-1H-indole-3-carbaldehyde	C9H7NO2	162.05	278.70	POS	0.006	0.708
Ferutinin	C22H30O4	357.21	422.60	NEG	0.011	0.697
Sebacic acid	C10H18O4	183.10	357.40	NEG	0.008	0.681
(R)-2',4',7-Trihydroxy-3',8-diprenylisoflavan	C25H30O4	395.22	447.10	POS	0.042	0.652
(2S,3R,4S,5S,6R)-2-[3-hydroxy-5-[(Z)-2-(4-hydroxyphenyl)ethenyl]phenoxy]-6-(hydroxymethyl)oxane-3,4,5-triol	C20H22O8	389.12	330.20	NEG	0.032	0.603
Piperonyl butoxide	C19H30O5	337.20	442.90	NEG	0.016	0.463
(-)-Camphoric acid	C10H16O4	199.10	295.70	NEG	0.018	0.461
2-(1-carboxyethyl)-5-methyl-cyclopentanecarboxylic acid	C10H16O4	199.10	295.70	NEG	0.018	0.461
Isoscopoletin	C10H8O4	191.03	286.10	NEG	0.015	0.313

Name: The identification of substances through qualitative analysis using tandem mass spectrometry matching; MZ: The median mass-to-charge ratio represents the mass-to-charge ratio of the peak in all sample; RT: Retaining the median retention time signifies the retention time of the peak across all samples.
