# Supplementary material for: Dietary Angelica sinensis Enhances Sow Lactation and Piglet Development Through Gut Microbiota and Metabolism
Source: Vet Sci. 2025 Apr 15;12(4):370. doi: 10.3390/vetsci12040370 (PMC12030784; doi:10.3390/vetsci12040370)
Supplement: Supplementary file 1 [file vetsci-12-00370-s001.zip › Supplementary Table 3.pdf]

Supplementary Table 3. Differentially down-regulated metabolites.

MS2 name	Formula	MZ	RT	Type	P-VALUE	LOG_FOLDCHANGE
H-D-Trp-OH	C11H12N2O2	203.08	207.60	NEG	0.003	-0.274
Pyrrolidine	C4H9N	72.08	48.70	POS	0.027	-0.410
DL-Tryptophan	C11H12N2O2	203.08	207.60	NEG	0.003	-0.274
Phenylalanine	C9H11NO2	166.09	108.10	POS	0.023	-0.243
14,16-dihydroxy-4-methyl-3-oxabicyclo[10.4.0]hexadeca-1(12),13,15-trien-2-one	C16H22O4	277.14	468.70	NEG	0.015	-1.078
Butylphthalide	C12H14O2	173.10	401.50	POS	0.006	-1.081
7,8-dimethoxychromen-2-one	C11H10O4	207.07	304.10	POS	0.045	-1.692
Ketoleucine	C6H10O3	129.06	248.10	NEG	0.035	-0.803
Isoquinoline	C9H7N	130.06	209.60	POS	0.000	-0.397
Tryptophan	C11H12N2O2	205.10	209.20	POS	0.001	-0.323
Phthalic acid mono-2-ethylhexyl ester	C16H22O4	277.14	468.70	NEG	0.015	-1.078
Neantine	C12H14O4	205.09	399.70	POS	0.007	-0.731
Scoparone	C11H10O4	207.07	304.10	POS	0.045	-1.692
Monobutyl phthalate	C12H14O4	205.09	399.70	POS	0.007	-0.731
Citropten	C11H10O4	207.07	304.10	POS	0.045	-1.692
D-Phenylalanine	C9H11NO2	166.09	108.10	POS	0.023	-0.243
trans-2-Ethoxy-5-(1-propenyl)phenol	C11H14O2	177.09	328.30	NEG	0.000	-9.131
3-Indoleacetonitrile	C10H8N2	157.08	209.60	POS	0.003	-0.348
3-Methylindole	C9H9N	132.08	209.20	POS	0.001	-1.304
L-3-Aminoisobutyric acid	C4H9NO2	104.07	104.70	POS	0.004	-1.877
9-oxo-nonanoic_acid	C9H16O3	173.12	396.80	POS	0.002	-1.225
Armillarilin	C24H30O7	431.21	434.60	POS	0.015	-1.899
Qunoline-8-methanol	C10H9NO	160.08	209.60	POS	0.002	-0.332
Rutarin	C20H24O10	463.11	209.20	POS	0.049	-0.356
1-Methoxy-3-carbaldehyde	C10H9NO2	174.06	207.70	NEG	0.047	-0.205
Archangelicine	C24H26O7	425.16	386.50	NEG	0.012	-2.809
7-[[(1S,4aS,6S,8aR)-6-hydroxy-5,5,8a-trimethyl-2-methylidene-	C24H30O4	402.19	411.20	POS	0.001	-2.971

3,4,4a,6,7,8-hexahydro-1H-naphthalen-1-yl]methoxy]chromen-2-one [IIN-based on						
Isomucronulatol 7-O-glucoside	C23H28O10	463.16	286.00	NEG	0.049	-1.461
Bifurcose	C24H42O21	667.23	210.50	POS	0.007	-0.400

Name: The identification of substances through qualitative analysis using tandem mass spectrometry matching; MZ: The median mass-to-charge ratio represents the mass-to-charge ratio of the peak in all sample; RT: Retaining the median retention time signifies the retention time of the peak across all samples.
